# Quantum coherence enhancement through control of metal–ligand covalency: modulating spin–orbit coupling in isostructural molecular qubits

**DOI:** 10.1039/d5sc09844k

**Published:** 2026-03-30

**Authors:** Subrata Ghosh, Paul H. Oyala, Maksym Fizer, Vsevolod D. Dergachev, Sergey A. Varganov, Natia L. Frank

**Affiliations:** a Department of Chemistry, University of Nevada 1644N. Virginia St. Reno NV 89557 USA nfrank@unr.edu; b Division of Chemistry and Chemical Engineering, California Institute of Technology Pasadena California 91125 USA

## Abstract

Manipulation of quantum systems for sensing and transduction rely on controlling the interactions between a quantum system and the many degrees of freedom of the bath. In molecular spin quantum systems, spin–orbit coupling serves as a conduit for energy dissipation *via* vibrational and phonon modes, which in turn are dictated by changes in oxidation state, metal–ligand covalency, and symmetry of the coordination sphere. The confluence of these factors complicate design strategies however for manipulation of spin qubits for quantum sensing and transduction strategies. Here, we report an investigation of the spin dynamics in isostructural *S* = 1/2 first-row transition metal complexes in which the spin–orbit coupling is varied between a *ls*-Co(ii)N_4_Phen (1-Co) and Cu(ii)N_4_Phen (1-Cu) complex. Based on free-ion spin–orbit coupling parameters (528 cm^−1^ for Co(ii) and 829 cm^−1^ for Cu(ii)), faster spin–lattice relaxation rates (1/*T*_1_) are initially expected for 1-Cu*vs.*1-Co. However, X-band pulsed EPR and AC susceptibility reveal that both complexes have nearly identical slow spin–lattice relaxation processes. Notably, decoherence (phase memory times, *T*_m_) at 60 K is longer for 1-Cu (0.63(1) µs) than for 1-Co (0.56(1) µs). Direct observation of d–d splittings, and determination of anisotropic *g*-values by EPR spectroscopy reveals an effective decrease in spin–orbit coupling for 1-Cu (*λ*′ = 400–435 cm^−1^) relative to 1-Co (*λ*′ = 370–400 cm^−1^) due to greater metal–ligand covalency in the Cu(ii) complex. Computational modelling of spin density distributions (DFT) and the excited state manifolds (CASSCF) support the differences in excited state energies and spin densities that dictate spin dynamics in these complexes. Two sets of nearly degenerate low-frequency modes were identified as possible vibrational relaxation channels *via* a two-phonon (Raman) process, consistent with contributions from spin–vibrational orbit interactions. This study provides fundamental insight into the role of metal–ligand covalency in modulating spin–orbit coupling contributions to spin–lattice relaxation and decoherence processes. Increased metal–ligand covalency reduces effective spin–orbit coupling, thereby increasing both spin–lattice and coherence time in molecular spin qubits, providing an important strategy for controlling quantum states and spin–vibrational energy transfer processes in molecular qubit platforms for quantum information processing.

## Abstract 2

Higher metal-ligand covalency in 1-Cu reduces effective spin-orbit coupling, extending the spin-relaxation and coherence times – a key strategy for tuning quantum coherence in molecular spin qubits.

## Graphical abstract

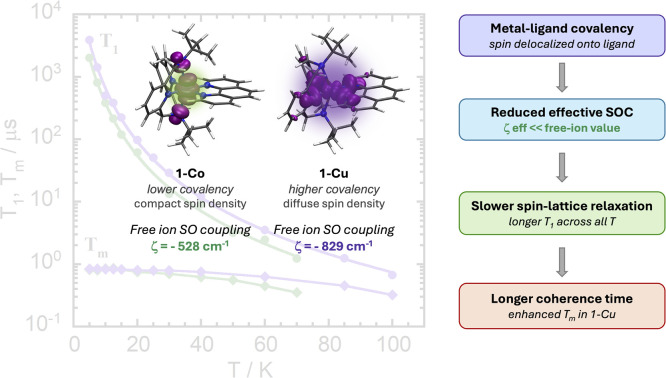

## Introduction

Effective control of quantum states in complex environments with many degrees of freedom is essential for quantum information processing protocols related to quantum computing, cryptography, communications, and sensing.^1–9^ Within spin-based quantum systems, the coupling of spin states to local and nonlocal vibrational (phonon) states *via* spin–orbit coupling is a major contribution to energy transfer processes between the quantum system and its environment. Spin–orbit coupling, an intra-atomic relativistic interaction that couples an electron-spin with its orbital momentum arising from local or bulk electric fields, can induce energy shifts and space inversion symmetry breaking. In nanomaterials, spin–orbit coupling arising from Dresselhaus^10^ and Rashba^11^ contributions induce spin polarization leading to spin Hall quantum effects,^12,13^ anomalous behavior in topological insulators,^14,15^ and chiral spin textures.^16,17^ In molecular and interfacial systems, spin–orbit coupling modulates intersystem crossing processes in the excited state, hyperfine (*A*-tensors) and *g*-tensors in spin qubits,^18,19^ transduction between optical and spin modalities for quantum communications and sensing strategies,^20–22^ chirality-induced spin selectivity (CISS) effects,^23,24^ and magnetic anisotropy in single-molecule and single-chain magnets.^25–29^ Attempts to quantify effective spin–orbit coupling strengths indirectly have proven difficult due to the confluence of adiabatic and nonadiabatic processes that contribute.

Understanding the unique role of spin–orbit coupling to spin relaxation processes in the absence of other structural factors is challenging. Spin–orbit coupled wave functions have been used to correlate the *T*_1_ anisotropy with the degree of spin–orbit coupling in isotropic *S* = 1/2 systems, Kramers ions with time-reversal symmetry, low spin–orbit coupling, and high-lying excited states.^30–36^ Temperature dependent spin–lattice relaxation times (*T*_1_) in 3d-transition and lanthanide metal complexes vary dramatically due to concomitant changes in oxidation state (IV *vs.* II), coordination number (six *vs.* four or five *vs.* four), symmetry, ligand rigidity, and metal–ligand covalency.^30–33,37–42^ Complications in precise molecular interpretation arise from the fact that both the temperature dependence of the spin–lattice relaxation time and *g*-anisotropy have contributions from spin–phonon coupling intensity, structural rigidity, and the energy of the first excited electronic state *via* second-order spin–orbit coupling terms.^18,33,40,43,44^ In addition, modulation of the *g*- and *A*-tensor *via* spin–phonon coupling suggests the importance of high symmetry modes to spin–lattice relaxation in high symmetry complexes.^37–42^

Recent studies highlight the importance of metal–ligand covalency in dictating nonadiabatic and excited state contributions to spin–phonon coupling. While studies of spin–phonon couplings have largely considered the direct coupling between phonons and the *M*_S_ sublevels, contributions from nonadiabatic terms and ligand field excited states are important.^45,46^ Recent analysis suggests two dominant contributions of spin–orbit coupling to spin–lattice relaxation: an adiabatic contribution that operates through direct modulation of the Zeeman splitting (*g*-tensor) and a nonadiabatic spin–vibrational orbit interaction that dominates at higher temperatures, represented as a Berry curvature on the ground-state potential energy surface of degenerate (non-Abelian) electronic states.^45^ In molecular systems, the nonadiabatic mechanism operates through modulation of the ligand field by lattice vibrations, which produces a fluctuating electric field that, in turn, modulates the orbital contribution. While there is no direct interaction with the electron spin, the spins feel the effect of modulation of the orbital motion through spin–orbit coupling, leading to spin–lattice relaxation. In addition, low-energy ligand field transitions may significantly increase spin–phonon coupling terms by introducing ground state orbital angular momentum through excited state spin–orbit coupling in which spin–phonon couplings derived from excited state coupling terms are sensitive to the absolute ligand field excited state energies.^46,47^

**Fig. 1:**
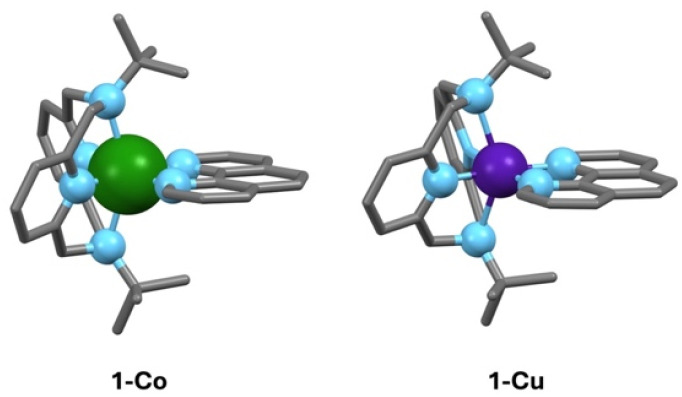
Isostructural (*S* = 1/2) metal complexes [*ls*-Co(ii)(^*t*^Bu-N4)(Phen)] (1-Co) and [Cu(ii)(^*t*^Bu-N4)(Phen)] (1-Cu) in which we modulate the spin–orbit coupling through changes in metal–ligand covalency. Color codes: carbon-grey, nitrogen- light blue, and cobalt-green, and copper-purple. Hydrogens are not shown for clarity.

Here, we demonstrate a strategy for directly modulating spin–orbit coupling through metal–ligand covalency, and quantify the effects on spin relaxation processes in two isostructural *S* = 1/2 mononuclear complexes, low-spin [Co(ii)(^*t*^Bu-N4)(Phen)] (1-Co) and [Cu(ii)(^*t*^Bu-N4)(Phen)] (1-Cu) (Fig. 1). The selection of cobalt(ii) and copper(ii) varies the metals' spin–orbit coupling constant, while maintaining ligand structures, coordination geometries, and oxidation state, which allows for comparative insight into the degree of metal–ligand covalencies in the complexes. The spin relaxation times were determined by dynamic magnetization measurements in the polycrystalline state and compared with spin dynamics characterized by pulsed X-band EPR, ENDOR, and Rabi nutation frequency measurements in dilute matrices. The direct observation of d–d splittings by electronic absorption spectroscopy, anisotropic *g*-factors by EPR, and electronic structure modelling (CASSCF) allows the direct calculation of the effective spin–orbit coupling for the two complexes. Despite copper having a much larger single-ion spin–orbit coupling parameter than *ls*-Co(ii), the increased metal–ligand covalency in Cu(ii) leads to a ∼50% reduction in spin–orbit coupling, making the effective spin–orbit coupling identical in the two complexes. The spin–lattice relaxation times (*T*_1_) are nearly identical, consistent with spin–orbit coupling as the dominant mechanism, with a shorter phase memory time (*T*_m_) in Co(ii) due to quantum tunnelling in restricted methyl groups. Spin–lattice relaxation in these complexes is dominated by Raman processes, with the latter involving significant contributions from spin–orbit interactions with nearly degenerate molecular modes as the major vibrational relaxation channel.

## Results and discussion

**Scheme 1:**
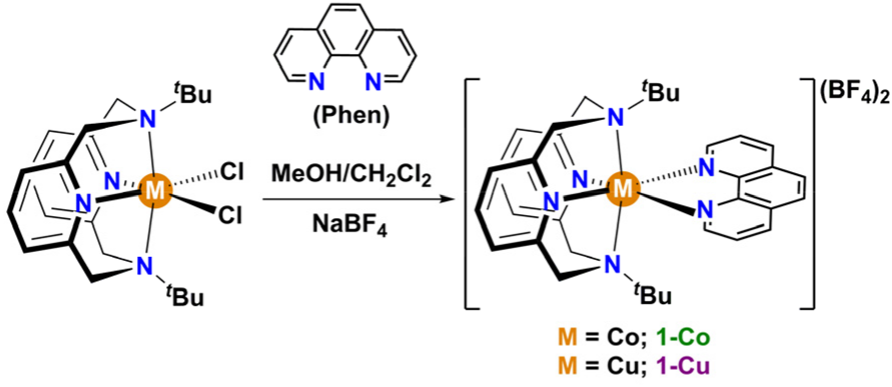
Preparation of isostructural complexes 1-Co and 1-Cu.

The preparation of isostructural *S* = 1/2 complexes required the utilization of a multidentate ligand that encouraged stabilization of the *ls*-Co(ii) state. The reaction of [M(^*t*^Bu-N4)Cl_2_] (M = Co(ii) and Cu(ii) and ^*t*^Bu-N4 = *N*,*N*′-di-*tert*-butyl-2,11diaza-[3,3](2,6)-pyridino-phane)^48,49^ with 1,10-phenanthroline was carried out in a (1 : 1) solvent mixture of methanol and dichloromethane followed by counteranion exchange with sodium tetrafluoroborate and crystallization to afford 1-Co and 1-Cu as orange-red and blue crystals in 57% and 80% yield, respectively (Scheme 1, and Fig. S2–26). Thermogravimetric analyses of 1-Co and 1-Cu (Fig. S1) reveal high thermal stability with no evidence of decomposition below 470 K, and notably no evidence for solvation in the lattice consistent with single crystal X-ray structure analyses (*vide infra*).

### Structural analysis

**Fig. 2:**
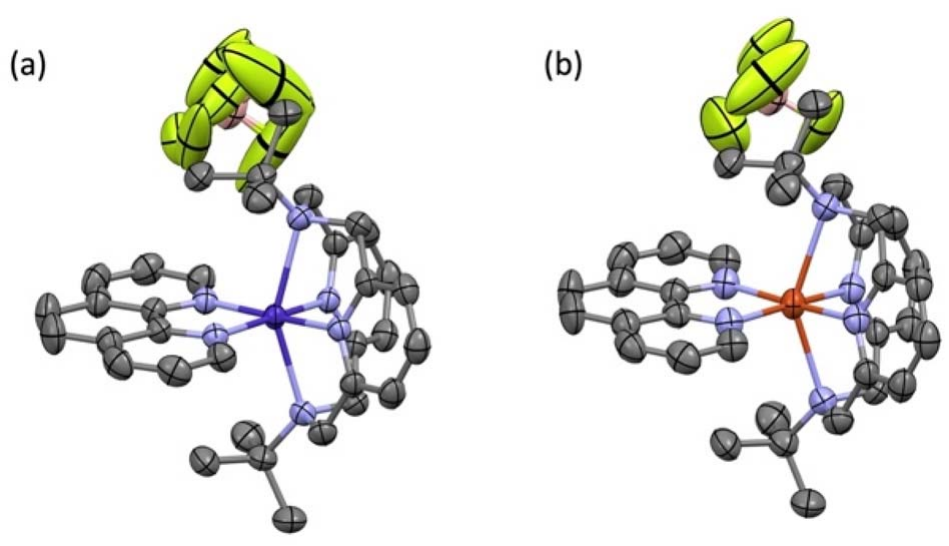
Structure of (a) [Co(^*t*^Bu-N4)phen]BF_4_ (1-Co) and (b) [Cu(^*t*^Bu-N4)phen]BF_4_ (1-Cu) from single crystal XRD at 100 K (75% probability). H atoms are removed for clarity. Color codes: Co, dark purple; Cu, orange, N, light purple; F, yellow-green; B, pink.

Single crystal X-ray diffraction (SC-XRD) of 1-Co and 1-Cu reveals axially distorted octahedral geometries, with identical degree of distortion and oxidation state, allowing direct correlation of spin dynamics with the nature of the central metal center in isostructural *S* = 1/2 complexes. X-ray diffraction analysis was performed on suitable single crystals of 1-Co at 296 K and 100 K, and of 1-Cu at 100 K (Tables S1–S3). Both complexes crystallize in the monoclinic space group *C*2_1_/*c* (*Z* = 8) and consist of mononuclear dicationic [M(^*t*^Bu-N4)(Phen)]^2+^ (M = Co; 1-Co and Cu; 1-Cu) complexes with two disordered BF_4_^−^ counterions (Fig. 2, S7 and S8). No solvent molecules are found in the crystal lattice of complexes 1-Co and 1-Cu, consistent with thermogravimetric analysis.

Structural analysis *via* SC-XRD at 100 K reveals molecular geometries consistent with *ls*-Co(ii) and Cu(ii) *S* = 1/2 complexes. The structure of the *ls*-Co(ii) complex was measured at two different temperatures (296 K and 100 K) in order to rule out spin transition processes quite common for *ls*-Co(ii) complexes.^50^ In this temperature range, no evidence of a significant structural distortion coincident with a spin transition to the high-spin state was found. The N_6_ coordination geometry in both complexes forms a distorted octahedral ligand field comprised of two axial amine nitrogen donors of the tetradentate macrocyclic ligand ^*t*^Bu-N4 and four N-heterocyclic donors in the equatorial plane. The average M–N bond distances of 2.084 Å and 2.157 Å for 1-Co and 1-Cu are in the expected range for the *ls*-Co(ii) and the Cu(ii) in a MN_6_ octahedral field, respectively.^51^ Desymmetrization of the octahedral ligand field *via* axial distortion is evident. The axial metal–nitrogen bond lengths are significantly longer (M–N1/N1′, 2.3581(14) Å 1-Co and 2.439(2) Å 1-Cu) than the equatorial metal–nitrogen bond lengths M–N2/N2′ bond (1.921(1) Å and 1.994(3) Å for 1-Co and 1-Cu) and M–N3/N3′ bond (1.973(1) Å and 2.038(2) Å for 1-Co and 1-Cu). The N1–M–N1′ angle is decreased (149.79(1)°, 1-Co and 147.72(13)°, 1-Cu) with decreased equatorial N3–M–N3′ bond angles of 82.81(8)° and 79.62(13)° for 1-Co and 1-Cu, respectively, consistent with axial distortion. The extent of distortion is significant for both complexes 1-Co and 1-Cu, as determined by continuous shape analysis of the MN_6_ coordinate geometry (CShM)^52^ (Table S4), with a slightly larger distortion geometry for 1-Cu (3.28) than 1-Co (3.12). The larger distortion in Cu *vs.* Co is reflected in the slightly higher octahedral distortion parameters (*∑*, *Θ*, and *ζ*) values in Cu *vs.* Co complex (Table S5). The extended lattice in the crystalline state is comprised of ribbons along the *b*-axis with large M⋯M intermolecular distances between two nearest [M(^*t*^Bu-N4)(Phen)]^2+^ units of 8.801(1) Å and 8.762(1) Å in 1-Co and 1-Cu, respectively. Based on SC-XRD analysis the complexes are expected to behave as paramagnets, with no magnetic exchange between complexes in the solid state, and only very weak dipolar interactions between metal centers. The spin dynamics in the solid state should therefore be dominated by molecular structure effects with contributions to decoherence from hyperfine and weak dipolar interactions.

### Electronic structure calculations and spectroscopy

**Fig. 3:**
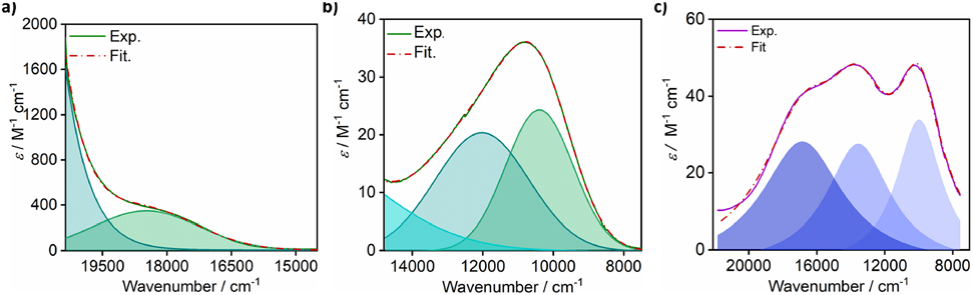
Electronic absorption spectra of d–d splittings with deconvolution for 1-Co (a and b) and 1-Cu (c) in 1 mM acetonitrile : toluene (1 : 1) at 300 K.

Solution state electronic absorption spectroscopy (EAS) on complexes 1-Co and 1-Cu reveal energies of the metal d–d transitions which can be used to experimentally determine the reduced spin–orbit coupling parameters directly (*vide infra*). The UV-vis-NIR spectra of complexes 1-Co and 1-Cu were measured in acetonitrile: toluene (1 : 1) at 300 K (Fig. 3, and S18–S20) and compared with predicted energy splitting from electronic structure calculations (Fig. S21). The electronic states involved in the transitions are labeled using the irreducible representations of the *D*_4h_ approximate point group of two complexes. The UV-vis-NIR spectrum of complex 1-Co exhibits two broad bands at 10 400 cm^−1^ (*ε* = 24 M^−1^ cm^−1^) and 12 020 cm^−1^ (*ε* = 20 M^−1^ cm^−1^), with a shoulder at ∼18 095 cm^−1^ (*ε* = 360 M^−1^ cm^−1^). Comparison to the calculated electronic structure allows assignment of the d–d transitions as ^2^A_1g_ → ^2^E_g_, ^2^A_1g_ → ^2^B_1g_, and ^2^A_1g_ → ^2^A_2g_, respectively, consistent with other *ls*-Co(ii) complexes.^53,54^ The high molar extinction coefficient of the higher energy band may be due to a tailing effect from the nearby metal-to-ligand charge transfer transition (MLCT).^53^ The UV-vis-NIR spectrum of complex 1-Cu exhibits three bands at 9980 cm^−1^ (*ε* = 34 M^−1^ cm^−1^), 13 555 cm^−1^(*ε* = 28 M^−1^ cm^−1^), and 16 850 cm^−1^ (*ε* = 28 M^−1^ cm^−1^). Comparison to the calculated electronic structure allows assignment of the d–d transitions as ^2^B_1g_ → ^2^A_1g_, ^2^B_1g_ → ^2^B_2g_, and ^2^B_1g_ → ^2^E_g_, respectively.

Interactions of the electron spin with an external magnetic field and nearby nuclear spins give rise to additional Zeeman and hyperfine terms, which can be described by an effective spin Hamiltonian (eqn (1)):

1 *Ĥ* = *µ*_B_*Ŝ****g****H* + *Ŝ****A****Î* + *λL̂*·*Ŝ*

where *µ*_B_ is the Bohr magneton, *Ŝ* is the electron spin operator, ***g*** is the *g*-tensor, *H* is the external magnetic field, ***A*** is the hyperfine tensor, *Î* is the nuclear spin operator, *λ* is the spin–orbit coupling constant, and *L̂* is the orbital angular momentum operator. In comparison to the spin Hamiltonian in the context of crystal field theory, experimental results can reveal a relativistic nephelauxetic effect that leads to an effective expansion of the metal–radial probability distribution function due to metal–ligand covalency^55–57^ The nephelauxetic effect leads to a reduction in the spin–orbit coupling constants relative to the free-ion value and is important to the computational modelling of *g*-values, hyperfine interactions, and zero-field splitting.^55,58^ The anisotropy in the covalency not only induces contributions of opposite sign of those induced by low-symmetry ligand field splitting but also leads to the appearance of charge transfer and ligand spin–orbit coupling contributions that are both found to be important. Three main factors contributing to the apparent reduction in the metal spin–orbit coupling constant are (a) the symmetry-restricted covalency that is directly related to the covalent mixing of the metal and ligand orbitals, (b) the central field covalency that is related to the change of the metal radial wave functions upon complex formation, and (c) the ligand spin–orbit coupling. Of these, (c) will usually be smallest unless the covalency is particular high and the ligand spin–orbit coupling constants are very large.^55^

**Fig. 4:**
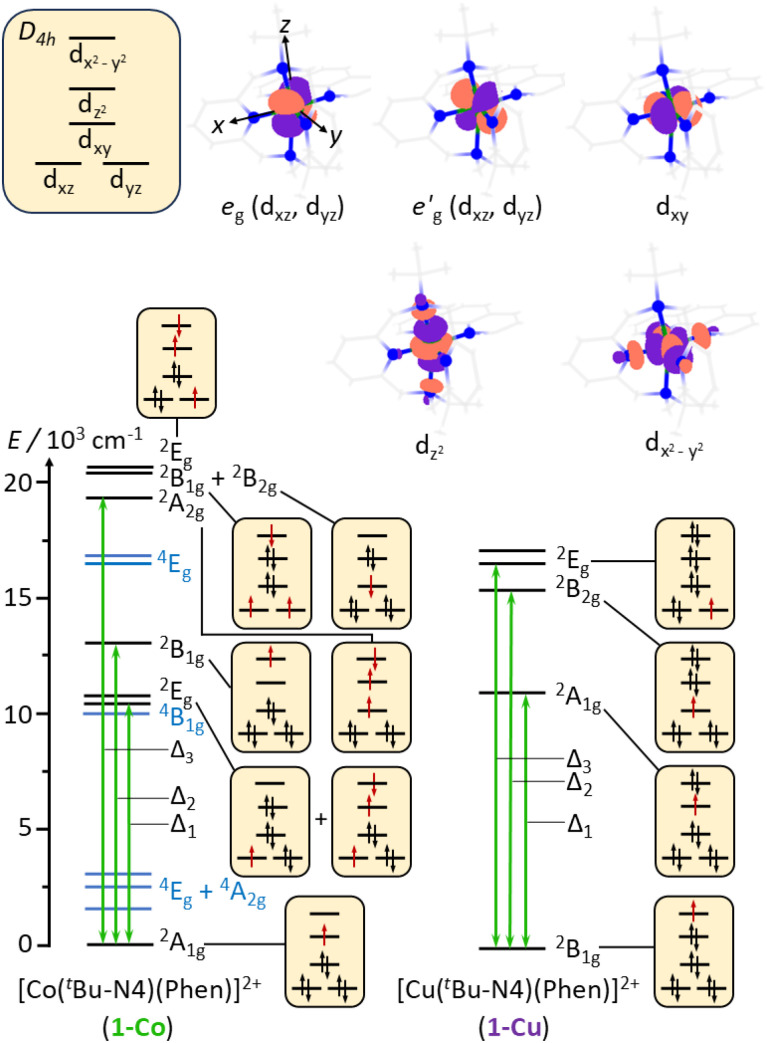
Splitting of the 3d orbitals in the approximate *D*_4h_ point group of the molecular fragments [Co(^*t*^Bu-N4)(Phen)]^2+^ of 1-Co and [Cu(^*t*^Bu-N4)(Phen)]^2+^of 1-Cu, calculated active space molecular orbitals, and CASSCF-NEVPT2 energy diagram of the low-lying electronic states with dominating electronic configurations of the doublet states (*S* = 1/2) (spin–orbit coupling not included). In configurations, unpaired electrons are red. The black labels indicate doublet states, and the blue labels indicate quartet states (*S* = 3/2).

In the isostructural complexes 1-Co and 1-Cu, the first coordination sphere (ligand field) of the ion is characterized by tetragonal distortion from the octahedral symmetry caused by elongation and bending of the M–N axial bonds. This tetragonal distortion results in the approximate *D*_4h_ point group symmetry of the ligand field. The relative order of the d_*z*^2^_ and d_*xy*_ orbitals is determined by the extent of the tetragonal distortion.^54^ In the present case, the d_*z*^2^_ orbital lies higher in energy than the d_*xy*_ orbital as predicted by the *ab initio* ligand field theory (AILFT) calculation (Table S16).^59^ Interaction of the d orbitals in the tetragonal ligand field stabilizes the doublet (*S* = 1/2) states (Fig. 4). Therefore, in 1-Co, which has the d^7^ configuration, the ground state is characterized by a singly occupied d_*z*^2^_ orbital and has the ^2^A_1g_ symmetry. In 1-Cu, with the d^9^ configuration, the highest-energy d_*x*^2^−*y*^2^_ orbital is singly occupied, and the ground state has the ^2^B_1g_ symmetry. For 1-Cu, the calculations predict the first excited (^2^A_1g_) state at 10 656 cm^−1^ followed by the ^2^B_2g_ state at 15 315 cm^−1^ and two nearly degenerate ^2^E_g_ states at 16 070 and 16 654 cm^−1^ (Fig. 4). In the 1-Co complex, there are low-lying excited ^2^E_g_ states at 10 815 and 10 952 cm^−1^, and the ^2^B_1g_ and ^2^A_2g_ states at 12 874 and 19 017 cm^−1^, respectively. Significant differences in the relativistic nephelauxetic effect are therefore expected for the two complexes, which in turn leads to changes in the effective spin–orbit coupling relative to the free-ion values.

The state average complete active space self-consistent field (SA-CASSCF) method together with the def2-TZVP basis set were used to model energies of the ground and excited states for both complexes. The active space included the d^7^ configuration of the metal ion for 1-Co and the d^9^ configuration for 1-Cu. For 1-Co, the ten lowest doublet states and all ten quartet states of the d^7^ configuration were included in state averaging. For 1-Cu, all five doublet states arising from the d^9^ configuration were included. The SA-CASSCF energies were corrected with the N-electron valence state second-order perturbation theory (NEVPT2). For 1-Cu, the calculations predict the first excited (^2^A_1g_) state at 10 656 cm^−1^ followed by the ^2^B_2g_ state at 15 315 cm^−1^ and two nearly degenerate ^2^E_g_ states at 16 070 and 16 654 cm^−1^(Fig. 4). In the 1-Co complex there are low-lying excited ^2^E_g_ states at 10 815 and 10 952 cm^−1^ and the ^2^B_1g_ and ^2^A_2g_ states at 12 874 and 19 017 cm^−1^, respectively.

**Fig. 5:**
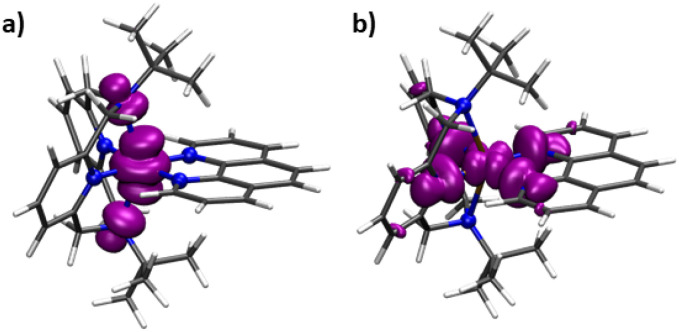
Calculated spin density isosurfaces for molecular fragments [Co(^*t*^Bu-N4)(Phen)]^2+^ of 1-Co (a) and [Cu(^*t*^Bu-N4)(Phen)]^2+^ of 1-Cu (b) at the B3PW91/EPR-II + CP(PPP) level of theory.

DFT calculations using the B3PW91 density functional and the CP(PPP) and EPR-II basis sets for metal ions and ligand atoms, correspondingly, were performed on 1-Co and 1-Cu to predict the spin density distribution. Single point calculations on the optimized geometries give singly occupied molecular orbital plots that are consistent with the magnetic orbitals of 1-Co and 1-Cu being dominated by the d_*z*^2^_ (1-Co) and the d_*x*^2^−*y*^2^_ (1-Cu) orbitals, with contributions from the equatorial nitrogens for 1-Cu, and axial nitrogens of the ^*t*^Bu-N4 ligand for 1-Co. Mulliken spin populations were found to be equal to 0.926 and 0.648 (metal ions) for 1-Co and 1-Cu, respectively (Fig. 5 and Table S23), suggesting greater spin delocalization onto the ligand for Cu(ii) compared to Co(ii), as expected. Mulliken spin populations on the axial amine (*N*_am_) and equatorial nitrogen atoms (*N*_Py_ and *N*_Phen_) are 0.052, −0.001, and −0.001 for 1-Co, and 0.000, 0.103, and 0.074 for 1-Cu, respectively.

**Fig. 6:**
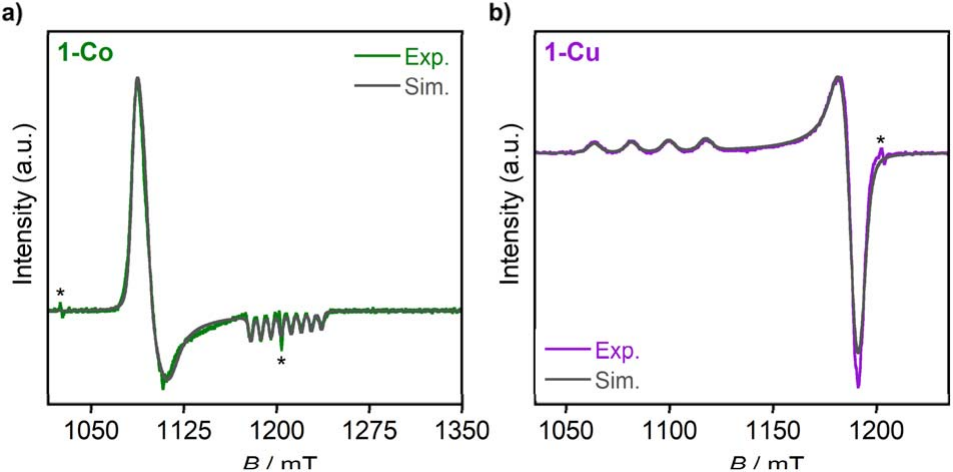
Q-band pseudomodulated EDFS spectra of 1-Co (a) and 1-Cu (b), with simulations (red). Asterisks above Q-band ESE-EPR spectra indicate the presence of background signals from Q-band resonator. Acquisition parameters: Q-band Pseudo modulated ESE-EPR: temperature = 18 K (1-Co), 24 K (1-Cu); MW frequency = 34.133 GHz (1-Co), 34.125 GHz (1-Cu); *τ* = 400 ns; MW π pulse length = 160 ns; shot rep. time = 1 ms, pseudomodulation amplitude = 2 mT.

In order to examine the electronic structures of the ground state, we performed a suite of continuous-wave (CW) EPR spectroscopy on frozen solutions (*ca.* 1 mM) of 1-Co and 1-Cu in a mixture of solvents (acetonitrile-d_3_ : toluene-d_8_; 1 : 1). X-band (9.6 GHz) CW-EPR and Q-band (34.1 GHz) spin echo-detected field swept (EDFS) spectra of each complex were acquired at 77 K (CW-EPR) and 18 K (Q-band EDFS, 1-Co) or 24 K (Q-band EDFS, 1-Cu), and are depicted with spectral simulations overlaid in Fig. 6 and S22. From these spectra, it can be observed that 1-Co exhibits an approximately axial EPR signal, with *g* values of *g*_*x*,*y*,*z*_ = [2.236, 2.217, 2.019] with *g*_*x*_, *g*_*y*_>*g*_*z*_, and *g*_iso_ of 2.157 (Table 1), consistent with an *S* = 1/2 ground state. An eight-line splitting pattern centered at *g*_*z*_ arises from the hyperfine interaction between the electron spin (Co(ii), *S* = 1/2) and the nuclear spin of cobalt (^59^Co, *I* = 7/2, 100% abundant) with a hyperfine coupling constant at this molecular orientation of *A*_*z*_(^59^Co) = 230.8 MHz, whereas there are no resolved hyperfine splittings observed at *g*_*x*_ or *g*_*y*_. The symmetry of the *g*-tensor, along with the largest hyperfine coupling being aligned with *g*_*z*_, is consistent with the presence of unpaired electron in the d_*z*^2^_ orbital with the electronic configuration (e_*g*_, 
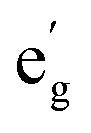
)^4^(d_*xy*_)^2^(d_*z*^2^_)^1^(d_*x*^2^−*y*^2^_)^0^. For 1-Co, the calculated principal values of the *g* tensor are *g*_*x*_ = 2.317, *g*_*y*_ = 2.310, and *g*_*z*_ = 2.078. The anisotropy of the tensor in the *xy* plane can be explained by the spin–orbit coupling between the ground ^2^A_1g_ state and the low-lying excited doublet ^2^E_g_ states (Tables S19 and S20). The small anisotropy of the *g* tensor in the *z* direction, observed experimentally, can be explained by the orbitally allowed but weak spin–orbit coupling to the excited doublet states of the A_2g_ symmetry (Table S17).^55^

**Table 1:** Combined experimental and NEVPT2/CASSCF predicted *g*-tensor parameters for 1-Co and 1-Cu

	*g*_*x*_, *g*_*y*_, *g*_*z*_	*g*_iso_	Δ*g* = *g*_iso_ − *g*_e_	|*g*_∥_ − *g*_⊥_|^a^
1-Co				
Experiment	2.236, 2.217, 2.019	2.157	0.1547	0.2075
def2-SVP	2.355, 2.350, 2.140	2.282	0.2797	0.2125
def2-TZVP	2.317, 2.310, 2.078	2.235	0.2327	0.2355

1-Cu				
Experiment	2.051, 2.056, 2.236	2.114	0.1117	0.1825
def2-SVP	2.081, 2.082, 2.362	2.175	0.1727	0.2805
def2-TZVP	2.081, 2.082, 2.367	2.177	0.1747	0.2855

a *g*_⊥_ = (*g*_*x*_ + *g*_*y*_)/2 and *g*_*z*_ = *g*_‖_.

Complex 1-Cu also exhibits an axial EPR signal, but with inverted symmetry, with *g* values of *g*_*x*,*y*,*z*_ = [2.051, 2.056, 2.236] *g*_*z*_ > *g*_*x*_, *g*_*y*_, and *g*_iso_ of 2.114, which are in the range that is observed for axially elongated copper(ii) systems.^60^ Here, *g*_*z*_ also exhibits resolved hyperfine splittings, with a quartet pattern and hyperfine coupling constant at this molecular orientation of *A*_*z*_(Cu) = 550 MHz, arising from the two naturally abundant *I* = 3/2 Cu nuclei (^63^Cu, 69.17% and ^65^Cu, 30.83%). The EPR results are also consistent with the splitting of the ^2^E_g_ ground state into the ^2^B_1g_ and ^2^A_1g_ states, and hence the presence of a significant amount of the tetragonal distortion in complexes 1-Co and 1-Cu. For 1-Cu, the calculated principal values of the *g* tensor are *g*_*x*_ = 2.081, *g*_*y*_ = 2.082, and *g*_*z*_ = 2.367. The large anisotropy of the tensor in the *z* direction can be explained by strong spin–orbit coupling between the ground ^2^B_1g_ and excited ^2^B_2g_ states (Table S18), while the anisotropy in the *xy* plane is induced by coupling to the two excited ^2^E_g_ states.

Increased metal–ligand covalency decreases the orbital angular momentum term L, leading to a decrease in spin–orbit coupling. The degree of reduction is conveniently quantified as the ratio *λ*′/*λ*, where *λ* is the free-ion spin–orbit coupling constant and *λ*′ the effective value in the complex. Using a combination of the *g*-values obtained from EPR and electronic energy levels directly obtained from the electronic absorption spectroscopy, the reduction of the spin–orbit coupling constant of complexes 1-Co and 1-Cu in the form of *λ*′/*λ* were determined.^60–62^ For the cobalt(ii) complex in a pseudo-*D*_4h_ ligand field and the ^2^A_1g_ ground state, calculation of the reduced spin–orbit coupling parameter *λ*′ was carried out using the perturbation relationships (eqn (2) and (3)), where *Δ*_1_ is the experimental energy separation between ground and excited states (*Δ*_1_ = Δ*E* (^2^A_1g_ → ^2^E_g_) = 10 400 cm^−1^), 
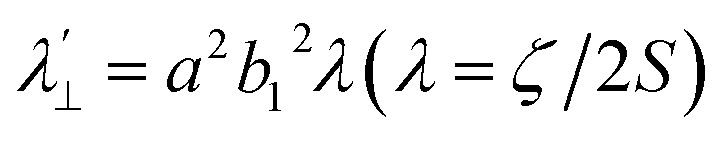
, where *ζ* is one-electron spin–orbit coupling constant (−528 cm^−1^), and *a*, and *b* are orbital coefficients of the d-orbitals of the ground and excited state. Importantly, because there is no low-lying excited orbital of the same symmetry along the *z*-axis, *g*_‖_ ≈ *g*_e_ = 2.0023 (eqn (3)), and hence only *g*_⊥_ provides a measure of *λ*′.2
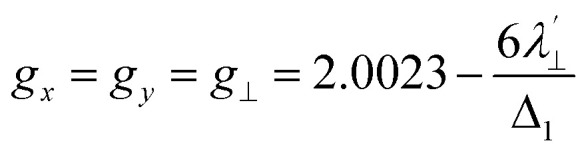

3 *g*_*z*_ = *g*_‖_ ∼ 2.0023

The EPR spectrum of the cobalt complex (1-Co) exhibits a pseudo-axial signal with *g*_*x*,*y*,*z*_ = [2.236, 2.217, 2.019] due to the non-degeneracy of the ^2^E_g_ and 
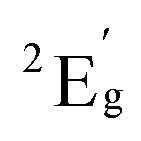
 states that are split by 137 cm^−1^ (Fig. 4, and Table S17). From the averaged perpendicular component *g*_⊥_ = (*g*_*x*_ + *g*_*y*_)/2 = 2.227, one obtains 
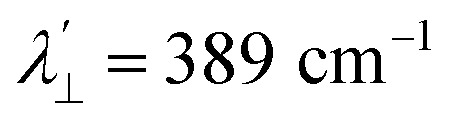
, corresponding to a 26% reduction relative to the free-ion constant 
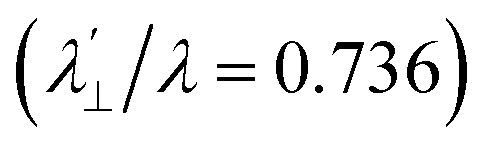
. If one considers the *x* and *y* components of the *g*-tensor independently, the reduced spin–orbit coupling constant 
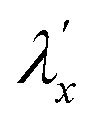
 and 
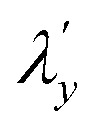
 estimated using eqn (4)–(6) with *Δ*_1′_ = *Δ*_1_ + 137 cm^−1^ = 10 537 cm^−1^ to give spin–orbit coupling constant of 23% for 
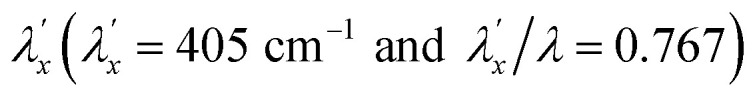
 and 29% for 
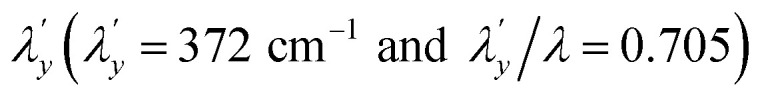
 relative to the free ion value (*λ* = – 528 cm^−1^), highlighting modest anisotropy in the spin–orbit coupling reduction.4
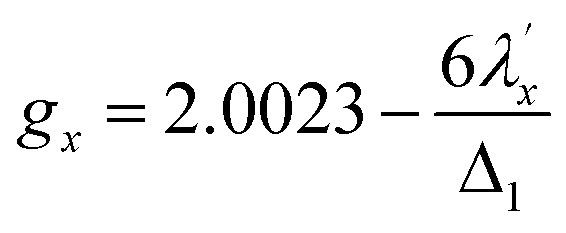
5
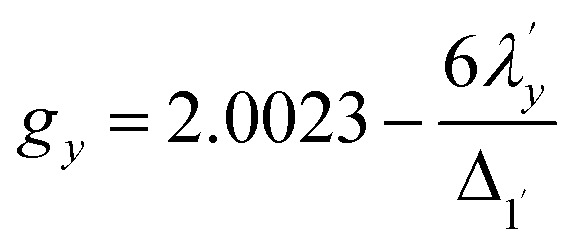

6 *g*_*z*_ ∼ 2.0023

For the copper(ii) complex in a pseudo-*D*_4h_ ligand field and d_*x*^2^−*y*^2^_ ground state, both axial and equatorial excitations are symmetry-allowed, so both *g*_‖_ and *g*_⊥_ deviate from *g*_e_. The perturbation treatment yields two distinct relationships (eqn (7) and (8)). So, we have applied the eqn (7) and (8) to calculate the reduced spin–orbit coupling (*λ*′) in which *Δ* is the energy separation between the ground and excited state (*Δ*_2_ = Δ*E* (^2^B_1g_ → ^2^B_2g_) = 13 555 cm^−1^; *Δ*_3_ = Δ*E* (^2^B_1g_ → ^2^E_g_) = 16 850 cm^−1^), 
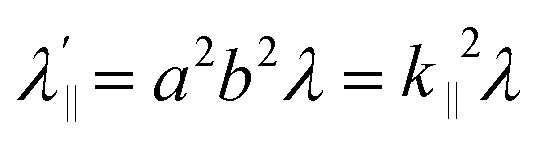
, 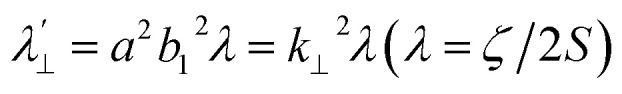, *a*, *b*, and *b*_1_ are orbital coefficients of the d-orbitals of the ground and excited states, and *k*_‖_ and *k*_⊥_ are the parallel and perpendicular components of the orbital reduction factor *k* (*k*^2^= (*k*_‖_^2^ + 2 *k*_⊥_^2^/3)) (Table S21).7
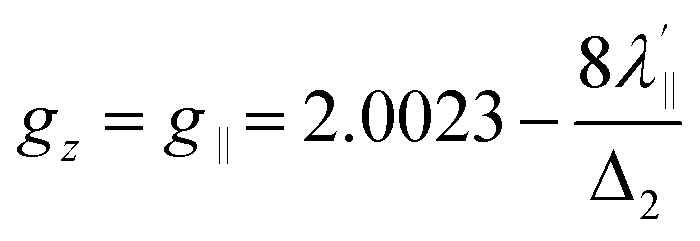
8
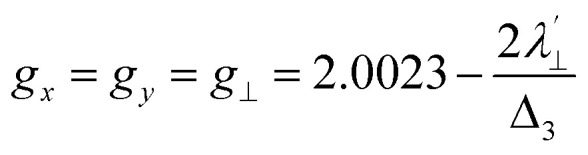
A more significant reduction of the spin–orbit coupling constant is observed in Cu(ii), in which a reduction of ∼47–52% 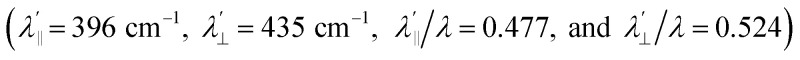 is estimated relative to the free ion value (*λ* = – 829 cm^−1^) with orbital reduction factor (*k*) of 0.713 for copper complex 1-Cu. Overall, the spectroscopic data suggests effective spin–orbit couplings for 1-Co of Co(ii) 
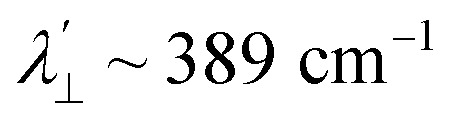
 and copper(ii) 
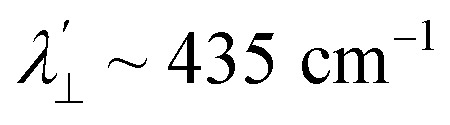
 and 
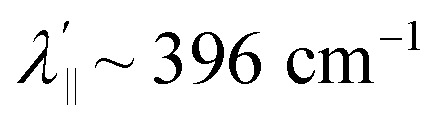
, respectively. These results suggest a greater reduction in the spin–orbit coupling relative to the free-ion value in 1-Cu due to stronger metal–ligand covalency as compared to 1-Co. The reduced spin–orbit coupling brings the effective spin–orbit coupling for both complexes to be roughly equivalent, suggesting that spin–lattice relaxation times should be quite similar for the two complexes, despite the difference in free-ion spin–orbit coupling typically used to predict spin–lattice relaxation times.

While the *g*-values and hyperfine *A*_*z*_ components of the Co and Cu hyperfine can be estimated from the previously discussed field swept CW- and EDFS EPR spectra, the smaller hyperfine tensor components *A*_*x*_ and *A*_*y*_ of the metal nuclei and all couplings from the six ^14^N nuclei of the nitrogenous ligands are concealed within the inhomogenously broadened linewidth of these spectra. In order to detect and determine these smaller hyperfine couplings quantitatively, we turned to Q-band electron nuclear double resonance (ENDOR) spectroscopy. Field-dependent Davies ENDOR spectra were collected on frozen solutions (*ca.* 1 mM) of 1-Co and 1-Cu the same mixture of solvents (acetonitrile-d_3_ : toluene-d_8_; 1 : 1) used for the X-band relaxation measurements, revealing signals from strongly coupled (*A* > 2 × *ν*_I_) ^14^N nuclei within the range from 5–30 MHz at all fields (Fig. S23 and S24). At many fields, broad, highly anisotropic signals are also observed from ^59^Co and ^63/65^Cu for 1-Co and 1-Cu, respectively, which are shown in wider ENDOR spectra. These spectra are well-simulated (in combination with the X-band CW-EPR and Q-band EDFS) by the parameters contained within Tables S24 and S25.

For 1-Co, a single class of hyperfine coupling to ^14^N is detected, with *A*_*x*,*y*,*z*_(^14^N_1_) = [21.6, 22.2, 31.9] MHz due to hyperfine coupling to the axial amine nitrogens. The four equatorial nitrogen ligands exhibit far less orbital overlap with the d_*z*^2^_ SOMO, and are predicted by DFT to be quite small (*ca.* 2 MHz) and unsuitable to detection by the Davies ENDOR technique used here. The additional ENDOR signals from ^59^Co (Fig. S23) allow for more quantitative evaluation of the smaller *A*_*x*_ and *A*_*y*_ principal components, thus providing an estimate for the full metal hyperfine tensor, as well as the NQI for this *I* = 7/2 nucleus. Best-fit simulations of the combined datasets provide A(^59^Co) = [−46.0, −82.7, 230.8], *e*^2^*Qq*/*h*(^59^Co) = −63.0 MHz and *η* = 0.11, in reasonable agreement with DFT predicted values (Tables S24 and S25), and consistent with predominant spin localization in a d_*z*^2^_ orbital, which would contribute a significant axial anisotropic hyperfine coupling component of the form [−2/5, −2/5, +4/5] × *ρ*(d_*z*^2^_).

The low-frequency region of the ENDOR of 1-Cu is considerably more complicated than that of 1-Co, with signals from at least two general classes of ^14^N nuclei evident in the range from 5 to 35 MHz, as well as extremely broad signals from ^63/65^Cu which overlap considerably at orientations near *g*_*x*_, *g*_*y*_ where the magnitude of this coupling is similar to that of the ^14^N nuclei (Fig. S24). The experimental data can be simulated with two distinct classes of ^14^N with different hyperfine coupling tensors *A*(^14^N_2_) = [46.3, 39.8, 35.8] MHz and *A*(^14^N_3_) = [35.9, 33.8, 32.1] MHz, both of which are significantly larger than *A*(^14^N_1_) measured for 1-Co. These values compare favorably with the DFT-predicted hyperfine and NQI parameters for the equatorial pyridine nitrogens (^14^N_2_, 
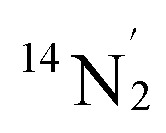
) of the N4 ligand and phenanthroline (^14^N_2_, 
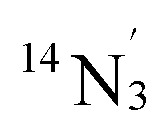
), respectively, therefore we assign these classes as described in Tables S24 and S25. In comparison to 1-Co, signals from the metal hyperfine in 1-Cu are rather poorly resolved due to extremely large ENDOR linewidths – reflective of the approximately doubled hyperfine anisotropy for this complex. Nonetheless, an estimate based on combined ENDOR and field-swept EPR spectral simulations provides an estimate of *A*(^63^Cu) = [14.7, 50.4, −550] MHz, *e*^2^*Qq*/*h*(^63^Cu) = 63.0 MHz and *η* = 0.10, which is in reasonable agreement with predicted values from DFT calculations (Table S24) and consistent with a d_*x*^2^−*y*^2^_ SOMO which would be expected to contribute a significant axial anisotropic hyperfine coupling component of the form [+2/5, +2/5, −4/5] × *ρ*(d_*x*^2^−*y*^2^_).

### Magnetization relaxation processes in the polycrystalline state

The magnetization at equilibrium and time-dependent magnetization *M*(*t*) at shorter timescales (0.1–10^4^ Hz) was probed *via* DC and AC susceptibility measurements in the polycrystalline state. The temperature dependent DC magnetization for 1-Co and 1-Cu were measured in the polycrystalline state from 2 to 300 K with external field of 1000 Oe (Fig. S11). Powder X-ray diffraction analyses were performed on polycrystalline samples of pure compounds (1-Co and 1-Cu) to ascertain their structural phase homogeneity, which is evident from the good agreement between experimental and simulated patterns (Fig. S9 and S10). The magnetic moment *χT* (emu K mol^−1^) is temperature independent consistent with paramagnetic behaviour. The moments of 0.45 and 0.46 cm^3^ mol^−1^ K for 1-Co and 1-Cu, respectively, are slightly higher than the spin-only values for octahedral *ls*-Co(ii) and a Cu(ii) (*S* = 1/2, *g* = 2.0; *χT* = 0.375 cm^3^ mol^−1^ K) due to *g*-anisotropy. The magnetic moment shows a slight decrease below 10 K for 1-Cu due to the weak dipolar interactions between the Cu(ii) centers (interatomic distance 8.762(1) Å) in the solid state. Magnetic moments measured by Evans' method in solution (acetonitrile-d_3_) at 300 K are 0.46 and 0.49 cm^3^ mol^−1^ K respectively, consistent with the values obtained from the solid state. Field dependent magnetization measurements were performed on 1-Co and 1-Cu from 0–7 T at 2, 3, 5, and 8 K (Fig. S12–S14). With increasing DC field (*H*), the magnetization reaches saturation magnetization of 1.08 and 1.15 *µ*_B_ at 2 K (7 T) for 1-Co and 1-Cu respectively, slightly higher than the spin-only value of 1.00 *µ*_B_. The magnetization saturation behavior at high magnetic field and the superposition in *M vs. H*/*T* curves are consistent with the absence of low-lying excited states. The *M vs. H*/*T* data were fit to a Brillouin function yielding *g*_iso_ values of 2.2053 and 2.2037 for 1-Co and 1-Cu respectively, consistent with a deviation from spin-only behavior due to *g*-anisotropy, in which the *g*-anisotropy is slightly higher in 1-Co than 1-Cu.

The differential susceptibility (d*χ*/d*H*) measured by AC magnetometry allows determination of the magnetization relaxation time (*τ*).^63^ Although both AC magnetometry and pulse EPR probe relaxation processes governed by spin–phonon coupling, the relaxation times extracted from these techniques correspond to distinct physical observables. The relaxation time *τ* obtained from AC susceptibility reflects the effective relaxation of the bulk magnetization and is determined by the slowest rate-limiting process governing ensemble magnetic dynamics; as such, *τ* may be influenced by collective effects, including dipolar interactions, spin diffusion, and phonon–bottleneck phenomena that impede efficient thermalization of resonant phonons. In contrast, pulse EPR directly measures the intrinsic spin–lattice relaxation time *T*_1_ of individual paramagnetic centers, which quantifies microscopic energy exchange between the spin system and the lattice. Consequently, *τ* and *T*_1_ are related but not equivalent quantities, and *τ* is typically one to two orders of magnitude longer than *T*_1_ in molecular magnetic systems where additional ensemble-level relaxation bottlenecks slow magnetization equilibration.^64–66^ When the magnetization relaxation rate is on the order of the AC field frequency (1/*τ* ∼ *ω*), *χ*′ in-phase and *χ*″ out-of-phase components to the AC susceptibility are frequency, field and temperature dependent, providing mechanistic and thermodynamic insight into the relaxation processes.

**Fig. 7:**
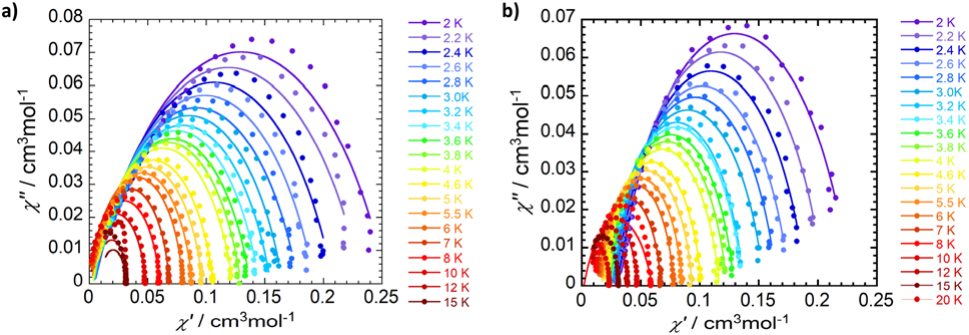
Experimental Cole–Cole plots with generalized debye fit for 1-Co (a) and 1-Cu (b) at 2500 Oe DC field at different temperatures.

**Fig. 8:**
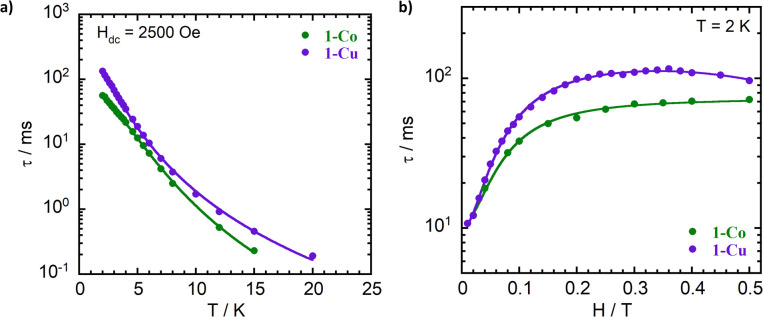
Dependence of the spin–lattice relaxation times (*τ*) on temperature (a) and magnetic field (b) for 1-Co (green) and 1-Cu (purple). The temperature-dependent data are fitted using the canonical Debye model (solid lines, left panel), while the magnetic field dependence is fitted using the Brons–Van Vleck model (solid lines, right panel).

Magnetization relaxation rates were evaluated through dynamic variable-frequency alternating-current (AC) susceptibility measurements in the polycrystalline state with an applied bias (DC) field. An Argand diagram plot of *χ*″ against *χ*′ allows determination of the relaxation time (*τ*) in which the maximum of *χ*″ which occurs at *ωτ* = 1. The temperature dependent relaxation times reveal a significant frequency dependence of both the in-phase (*χ*′) and out-of-phase (*χ*″) components from 2 to 20 K (Fig. 7, S15–17, and Tables S6–S9). The complexes exhibit slow magnetic relaxation in the measured temperature range (Fig. 8) with a faster relaxation time *τ* for 1-Co (*τ* = 56 ms at 2K) than for 1-Cu (*τ* = 130 ms at 2 K). The temperature dependent relaxation time exhibits a change in slope with increasing temperature due to a change in phonon distribution. With increasing temperature, the population of vibrational modes that participate in two-phonon processes increases, leading to a greater contribution from Raman relaxation pathways. The relaxation rate (*τ*^−1^) was fit to the generalized Debye model^49^ including direct and Raman processes (*τ*^−1^ = *aT* + *bT*^*n*^), in which the coefficients *a*, *b* and *n* correspond to the contributions from the resonant direct process, the Raman contribution, and order of the temperature dependence, respectively (Tables S6 and S8). Best fit parameters suggest contributions from both direct and Raman processes for 1-Co (*a* = 7.9(1) s^−1^ K^−1^; *b* = 0.04(1) s^−1^ K^−*n*^, and *n* = 4.27(5), and 1-Cu*a* = 2.8(1) s^−1^ K^−1^; *b* = 0.13(1) s^−1^ K^−*n*^, and *n* = 3.59(5)). In intermediate temperature ranges, the Raman mechanism dominates in which the system dynamics is restricted to the lowest Kramers doublet, and excited states contribute *via* nonresonant first- and second-order Raman processes.

The Raman exponents (*n*) for both complexes are lower (*n* ∼ 3) than that expected for second-order coupling of the acoustic modes of the phonon spectrum in a crystalline solid (*n* = 9), as has been observed for other *ls*-Co(ii) (*S* = 1/2) and Cu(ii) (*S* = 1/2) complexes.^31,67–70^ The lower Raman exponents can be attributed to either the involvement of both acoustic and optical phonons to the Raman process^71^ or the dominant role of local vibrational modes. A vibrational density of states less than predicted by the Debye model can lead to exponential temperature dependence, contributing to anomalously low Raman exponents.^72,73^ The latter interpretation is consistent with our suggestion that the Raman mechanism in these systems is dominated by pairs of nearly degenerate low energy modes (*vide infra*). The relaxation time was investigated as a function of the applied static magnetic field (100–5000 Oe) at constant frequency and temperature (2 K) (Fig. 8, Tables S7, and S9). The relaxation time (*τ*) increases with increasing external field with a plateau around ∼30 mT, followed by a decrease at higher fields. The non-monotonic behavior of *τ vs. H* is consistent with opposing contributions from spin–phonon and spin–spin interactions. Larger external fields can lead to a more efficient direct mechanism due to increased Zeeman splitting and a higher phonon-density at energies matching the separation between the *m*_s_ = ± 1/2 levels. In this case, contributions from field independent Raman-I contributions which are proportional to (*T*^*n*^) and Raman-II contributions proportional to *B*^2^*T*^m^ are expected for a Kramer's spin system.^33,74^ On the other hand, increasing external field can also suppress the mixing of energy levels due to spin–spin (dipolar) and spin–nuclei (hyperfine) interactions which facilitate relaxation.

Use of the Gorter–Van Vleck–Hebel–Slichter (G.V.H.S.) formalism leads to a dependence of relaxation time on field 1/*τ* ∝ (*B*^2^ + *µB*_hyp_^2^ + 1/2*µ*′*B*_dip_^2^)/(*B*^2^ + *B*_hyp_^2^ + 1/2*B*_dip_^2^) in which the additional Brons–Van Vleck prefactor accounts for the effect of the internal fields due to dipolar *B*_dip_ and hyperfine interactions *B*_hyp_^2^.^75,76^ To account for these two contributions, the *B* dependence of the relaxation rate can be fit by the Brons–Van Vleck model^76,77^ (eqn (9)) in which *c* is the contribution from the direct process, *d* ∼ *B*^2^ represents the zero-field relaxation rate due to quantum tunneling, and the ratio *e*/*f* is proportional to the contributions of *µ* and *µ*′, the relative contribution of dipolar and hyperfine internal fields to the relaxation.9
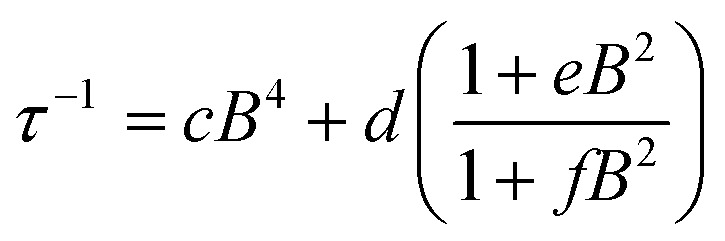


The field dependence of the relaxation for the two complexes were well reproduced by the Brons–Van Vleck model to give (*c* = 0, *d* = 94(4) s^−1^; *e* = 77(6) T^−2^; and *f* = 539(55) T^−2^ (*e*/*f* = 0.14)) for 1-Co, and *c* = 36(4) T^−4^ s^−1^; *d* = 102(2) s^−1^; *e* = 59(2) T^−2^; and *f* = 810(29) T^−2^ (*e*/*f* = 0.07) for 1-Cu. In 1-Co, the field dependent relaxation was well fit without a direct process (c) term, suggesting that two phonon processes dominate over one-phonon processes in this complex, in the solid state. The lower *e*/*f* ratio for 1-Cu indicates weaker contributions to relaxation from hyperfine and dipolar interactions relative to the cobalt complex, which provides insight into analysis of the decoherence time (*vide infra*).

### Spin dynamics in dilute matrices

Spin–lattice relaxation occurs through spin–vibrational coupling, and can be measured through an inversion recovery sequence, which in principle leads to magnetization relaxation along the *z* axis as a function of time following the Bloch equation (*M*_*z*_ = *M*_o_ (1 − exp(−*t*/*T*_1_))). Often the magnetization relaxation does not follow a simple Bloch equation due to spectral diffusion processes that include contributions from diffusion of an anisotropic paramagnetic center, electron–electron exchange, electron–nuclear cross relaxation and nuclear spin flip-flops.^78^ The spectral diffusion contributions can be accommodated by inclusion of the *β*-stretch parameter. The temperature dependence of the spin–lattice relaxation rates (*T*_1_) were determined by X-band inversion recovery experiments at temperatures 5–70 K for 1-Co and 5–100 K for 1-Cu, respectively (Fig. S25–S29, and Tables S10–S14). The resulting inversion recovery traces were fit with a stretched monoexponential equation (eqn (10)) where *I* is the echo intensity, *τ* is the delay between the initial pulse and the echo detection, and *β*_1_ is the stretch factor.10
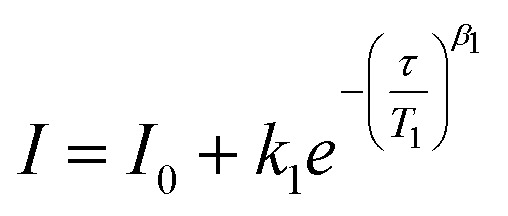


**Fig. 9:**
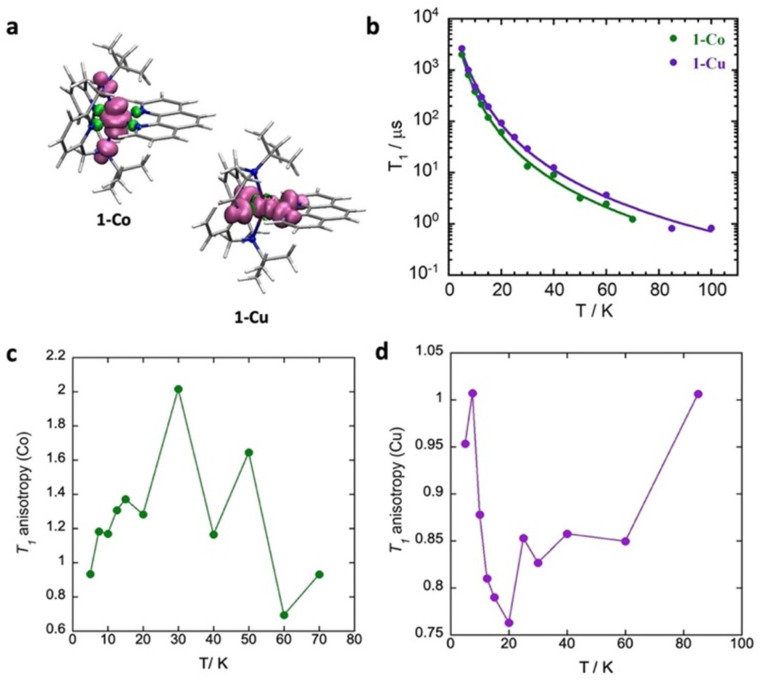
(a) Total spin density calculated using the B3PW91/EPR-II + CP(PPP) method (see SI for details), (b) temperature dependence of spin–lattice relaxation (*T*_1_) for complex 1-Co at 341.5 mT (*g*_‖_) and 1-Cu at 340.28 mT (*g*_⊥_) in acetonitrile-d3 : toluene-d8 (1 : 1), full lines are the best fits of the models with the combination of direct and Raman processes, (c) *T*_1_ anisotropy for 1-Co and (d) *T*_1_ anisotropy for 1-Cu in a dilute solvent matrix.

As illustrated in Fig. 9, the spin–lattice relaxation times (*T*_1_) for 1-Co are lower than those for 1-Cu across the entire measured temperature range. This observation is consistent with the temperature dependence of the spin–lattice relaxation time (*τ*) in polycrystalline samples. The relaxation times decrease with increasing temperature from 2.63(1) ms (1-Cu) and 2.01(5) ms (1-Co) at 5 K to 1.13(3) µs (1-Cu) and 0.83(1) µs (1-Co) at 100 K and 70 K, respectively. While various analytical functions are commonly employed to describe the temperature dependence of *T*_1_,^79^*T*_1_ was modeled with a combination of a direct process and Raman I processes based on the Debye model (eqn (11)), where *a* is the one-phonon direct process contribution, *b* is the two-phonon Raman contribution, and *n* is the order of the temperature dependence for the Raman I process.^80^ Due to the low-temperature measurement range (5–100 K), high lying excited states and errors arising from over-parameterization, the Orbach process, Debye transport integral function and local mode contributions were excluded from the *T*_1_ model.^79,81^

11 *T*_1_^−1^ = *aT* + *bT*^*n*^

The temperature dependent spin relaxation behavior was well-reproduced with best-fit parameters of *a* = 46(21) s^−1^ K^−1^; *b* = 2.2(7) s^−1^ K^−*n*^, and *n* = 3.00(10) for 1-Co, and *a* = 24(8) s^−1^ K^−1^; *b* = 1.18(2) s^−1^ K^−*n*^, and *n* = 3.03(5) for 1-Cu, in which both direct and Raman I processes contribute to relaxation. The Raman exponents (*n*) for 1-Co and 1-Cu are lower than that expected for a perfect Kramer's doublet (*n* = 9), due to the contributions of both acoustic and optical phonons to relaxation processes.^67^ Both direct and Raman coefficients are a factor of two larger for the cobalt complex, consistent with faster relaxation in cobalt as arising from both processes, within the Debye model.

Recent investigations highlight the role of localized vibrational states as critical to spin–lattice relaxation processes, rather than phonon distributions as described by the Debye model.^18,30,32,38,41,43,46,47,82–86^ In general, the higher the spin–orbit coupling, the stronger the spin–phonon coupling, and the faster the spin–lattice relaxation. The average magnitude of the spin–phonon coupling correlates with the *g*-anisotropy, which is evidenced by a static g-shift (from the free electron value *g*_e_ = 2.0023) and the energy of the first excited electronic state *via* second order spin–orbit coupling.

In general, the larger the spin–phonon coupling, the larger the *g*-anisotropy. Despite the larger free-ion spin–orbit coupling for Cu(ii) over *ls*-Co(ii), comparison of the *g*-anisotropy, (defined as Δ*g* = *g*_iso_ − *g*_e_ and |*g*_∥_ − *g*_⊥_| in which *g*_⊥_ = (*g*_*x*_ + *g*_*y*_)/2 and *g*_*z*_ = *g*_∥_) reveals larger *g*-anisotropy for 1-Co (Δ*g* = 0.1547) over 1-Cu (Δ*g* = 0.1117), consistent with faster spin–lattice relaxation in the *ls*-Co(ii) complex. The higher *g*-anisotropy suggests greater effective spin–orbit coupling for cobalt, relative to copper, due to a reduction in the Cu(ii) spin–orbit coupling by the nephelauxetic effect induced by metal ligand covalency.

Determination of the *T*_1_ anisotropy, defined as (1/*T*_1_(⊥))/(1/*T*_1_(‖)) provides insight into the roles of hyperfine interactions, spin–orbit coupling and spectral diffusion in spin–lattice relaxation processes.^30,79,87–89^ Modulation of the hyperfine interaction has been proposed as a mechanism for spin–lattice relaxation by the Raman process and anisotropy of the hyperfine and Zeeman tensors. The *T*_1_ inversion recovery measurements were carried out with respect to the external field (Fig. 9, S30, and S31) approximately corresponding to perpendicular (*g*_*x*_, *g*_*y*_) molecular orientations (313.4 mT for 1-Co and 340.28 mT for 1-Cu) and parallel molecular orientations (*g*_*z*_) (341.5 mT for 1-Co, and 312.70 mT for 1-Cu). For each of these paramagnetic centres the local symmetry is approximately axial, and symmetry arguments suggest that there are more vibrational modes with contributions in the equatorial plane then along the symmetry axis, consistent with the observed orientation dependence of the relaxation rates.

The temperature dependent *T*_1_ anisotropy (Fig. 9c and d) is roughly a factor of two larger for the *ls*-Co(ii) complex, in which *T*_1_ > 1 suggests faster relaxation along (*g*_⊥_). As the hyperfine interaction is smaller in the *xy* plane (∼35 MHz) the anisotropy of the hyperfine interaction does not appear to be the principal source of the orientation dependence of *T*_1_ for *ls*-Co(ii) complex. In the Cu(ii) complex, on the other hand, relaxation is faster along the *z*-axis (*g*_‖_) consistent with the hyperfine anisotropy (*A*_*z*_ = 230 MHz).

To a first approximation, the unpaired electron in Cu(ii) occupies the d_*x*^2^−*y*^2^_ atomic-like orbital, while in the *ls*-Co(ii) complex, the spin density is dominated by the d_*z*^2^_ atomic-like orbital. In both cases, *g*-anisotropy is due to spin–orbit coupling. When the magnetic field is along the *z*-axis (B_‖_), spin–orbit coupling adds a contribution from d_*xy*_ to the first-order wavefunction. In the perpendicular plane the spin–orbit coupling adds a contribution from d_*xy*_, d_*yz*_ to the first-order wavefunction, but in this case, the added contribution has the opposite sign, leading to a negative contribution. Since spin–lattice relaxation requires a change from *m*_s_ +1/2 to *m*_s_ −1/2, vibrations in the *xy*-plane that modulate the spin–orbit coupling may have more impact on relaxation than vibrations along the *z*-axis.^30,87^ Ignoring contributions from spectral diffusion, there is a greater contribution of spin–orbit coupling to spin–lattice relaxation in *ls*-Co(ii) cobalt relative to Cu(ii).

### Decoherence time

**Fig. 10:**
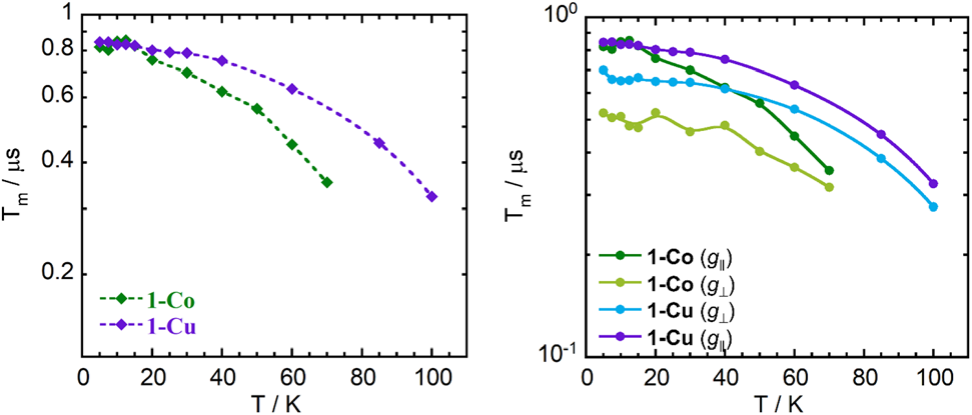
Temperature dependence of phase memory time (*T*_m_) for complexes 1-Co and 1-Cu (left), and at different magnetic fields (*g*_‖_ and *g*_⊥_) (right) in acetonitrile-d_3_ : toluene-d_8_ (1 : 1).

The spin-echo dephasing time, *T*_m_, encompasses all processes leading to electron spin decoherence including *T*_2_, the temporal evolution of magnetization in the *xy*-plane with dephasing and energy loss *via* dipolar interactions, electron–electron exchange, electron–nuclear cross relaxation, nuclear spin flip-flops, librational/rotational motion of nuclei and nuclear spin diffusion. Multi-pulse approaches are typically employed using the Hahn Echo sequence (π/2–*τ*–π–*τ*) in which the *τ* time between the pulses is varied. The echo amplitude is expected to decay exponentially as a function of delay time *τ*, (exp(−2*τ*/*T*_m_)) to give the phase memory time *T*_m_. If the system follows Bloch behavior, then *T*_m_ = *T*_2_. However other contributions to the decay can arise from vibrational motion of the paramagnetic species and nuclear spin diffusion, requiring a stretched exponential fit or a fit to bi- or multi-phasic kinetics. Hahn-echo decay measurements were performed on the *ls*-Co(ii) and Cu(ii) complex in order to determine the temperature dependence of the phase memory time (*T*_m_) (Fig. 10 and S32–S35, and Tables S10–S13). The resulting decay traces were fit with a stretched monoexponential equation (eqn (12)) where *I* is the echo intensity, 2*τ* is the delay between the initial pulse and the echo detection, and *β*_m_ is the stretch factor.12
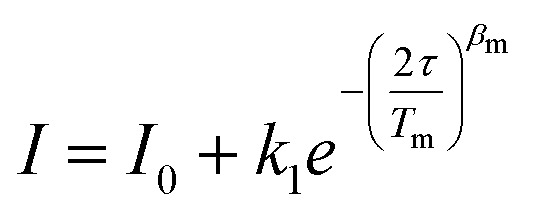


The phase memory time *T*_m_ shows very little temperature dependence in the low temperature range (5–20 K), with 1-Co exhibiting slightly longer phase memory time (*T*_m_ ∼0.83 µs), than 1-Cu (*T*_m_ ∼0.80 µs) at perpendicular molecular orientations (Fig. 10). Decoherence and dephasing increase with increasing temperature as expected, with a stronger temperature dependence observed in 1-Co relative to 1-Cu. Additionally, the determination of the field dependence of *T*_m_ at a constant temperature reveals larger *T*_m_-anisotropy for 1-Co than 1-Cu.

A comparison of the phase memory times (*T*_m_) for the 1-Co and 1-Cu complexes reveals that, although these complexes are isostructural, the copper complex exhibits a significantly longer coherence time than the cobalt complex. This disparity in *T*_m_ becomes increasingly pronounced at higher temperatures. The primary factors contributing to decoherence and homogeneous broadening are spectral diffusion, which includes electron–electron dipolar interactions, pulse-induced instantaneous diffusion, Heisenberg exchange, electron–nuclear cross relaxation driven by hyperfine interaction, and librational or rotational motion of nuclei, as well as spatial diffusion, which involves nuclear dipolar interactions leading to macroscopic polarization transfer.^79,90–92^

Given that both complexes are isostructural and share the same concentration and spin environment within the solvent matrix, nuclear diffusion is expected to dominate in both cases. Instantaneous diffusion arising from electron–electron dipolar coupling should remain constant between the two systems due to broad resonances resulting from hyperfine coupling and g A^−1^-anisotropy. Hyperfine couplings determined by ENDOR reveal substantial hyperfine coupling in both the copper complex (*A*_*iso*_ for ^63^Cu is 116 MHz) and the cobalt complex (*A*_*iso*_ for ^59^Co is 34 MHz), with relatively large superhyperfine coupling to ligand nitrogen nuclei for Cu (*A*_*iso*_(*N*) approximately 35–45 MHz for heteroaromatics) and Co (*A*_*iso*_(*N*) approximately 24 MHz for axial amines). Although the hyperfine coupling to N6 core nitrogens is significant and varies between the two complexes, the average M–N bond distances of 2.084 Å for 1-Co and 2.157 Å for 1-Cu indicate that the nitrogen nuclei reside within the ‘spin-diffusion barrier’ (3–10 Å).^90,93–96^ These nuclei are ‘hypershifted’ due to strong electron–nuclear hyperfine interactions and may undergo efficient polarization transfer from nuclei in the bulk, although some evidence exists for diffusion within the spin-diffusion barrier due to multinuclear–electron flip-flop processes and dipolar field anisotropy.^97–101^

The Hahn echo decay curves were analyzed using a stretched exponential model, where the exponential factor *β* provides insight into the rate of the process (*W*) contributing to dephasing relative to *τ*, the time between pulses.^79^ However, the concentration dependence of *β* may indicate more complex underlying mechanisms.^102^ In this study, since the Hahn echo decay curves were obtained under identical concentration conditions, the relative rates of dephasing *versus T*_m_ for the two complexes were extracted to elucidate the origins of the observed differences in decoherence rates. For the copper complex, the exponent *β* ranges from approximately 0.8 to 1.1 (Table S13), while for the cobalt complex, *β* is lower, ranging from approximately 0.5 to 0.7 (Table S12). This finding is consistent with a faster dephasing process in the cobalt complex.

Dephasing may result from the averaging of hyperfine coupling to the three hydrogens of methyl groups under restricted rotational conditions, which significantly influences the temperature dependence of *T*_m_.^103–107^ Quantum tunneling between different rotational orientations of methyl groups generates fluctuating local magnetic fields (spectral noise) due to anisotropic hyperfine interactions, thereby contributing to spectral diffusion (homogeneous broadening) and phase memory loss. Methyl groups with high rotational barriers, such as those in *t*-butyl groups, can couple more strongly to electron spin levels, amplifying this effect. Analysis of the hyperfine coupling constants shows that *A*_*iso*_(*N*) (amine) is approximately 22–24 MHz in the cobalt complex, compared to approximately 1 MHz in the copper complex. The lower spin density on the Cu–N (*t*-butyl) methyl groups results in weaker contributions from rotational averaging than in the cobalt complex. The more pronounced temperature dependence of the 1-Co spin coherence time (*T*_m_) aligns with the averaging of electron–nuclear couplings due to hindered rotation of alkyl groups, which is the primary factor underlying the differences in phase memory decay between the two complexes. A plot of log(1/*T*_m_) *versus* log *T* (Fig. S38) demonstrates the expected temperature-dependent fluctuation due to methyl group hindered rotation in Co(ii),^79^ whereas the Cu(ii) complex exhibits relatively constant behavior with temperature, supporting the significant role of nuclear spin diffusion. In conclusion, the phase memory time of both complexes is primarily governed by nuclear spin diffusion, with an additional contribution from hindered methyl group rotation in the Co(ii) complex due to higher spin density on the axial nitrogens and *t*-butyl methyl groups relative to the Cu(ii) complex.

**Fig. 11:**
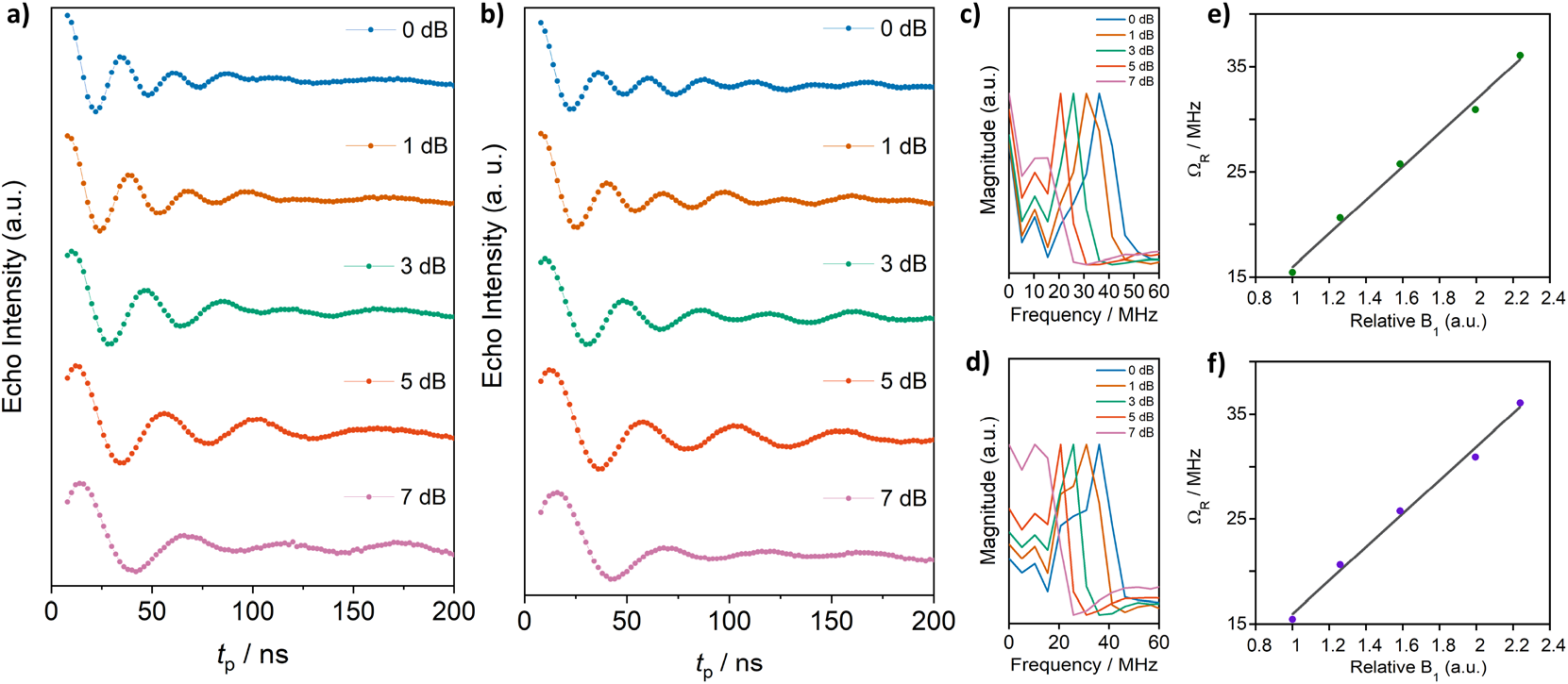
Rabi oscillations for complexes 1-Co (a) and 1-Cu (b) in acetonitrile-d_3_ : toluene-d_8_ (1 : 1) were recorded at 20 K for different microwave attenuations (0–7 dB) at 314.5 mT for 1-Co and at 340.28 mT for 1-Cu respectively. Fourier transform of the Rabi oscillations for complexes 1-Co (c) and 1-Cu (d). Linear dependence of the Rabi frequency (*Ω*_R_) as a function of the relative microwave attenuation *B*_1_ for 1-Co (e) and 1-Cu (f).

Additional insight into the coherent dynamics of the system can be gained by analyzing the frequency components of Rabi oscillations (Fig. 11). Rabi oscillations are periodic modulations in the population of a two-level quantum system interacting with a time-varying field^108^ in which the frequency of the Rabi oscillations is determined by the strength of the interaction between the quantum system and field. For spin qubits, application of microwave pulses of duration *t*_p_ and field B_1_ are applied leading to rotation of the spins on the Bloch sphere by an increasing nutation angle that depends on *t*_p_ and B_1_. In the slow spin–spin relaxation window, 1/*T*_2_ ≪ *ω*_1_ oscillation of the magnetization along the *z*-axis occurs with varying *t*_p_ to give Rabi oscillations (Rabi nutation frequency *ω*_1_ = *µ*_B_g*B*_1_/*ℏ*) demonstrating that the qubit can be placed in an arbitrary superposition state on the Bloch sphere.

Nutation studies were carried out with a variable length microwave pulse (nutation pulse or tipping pulse (*t*_p_)) applied to complexes 1-Co and 1-Cu at 20 K using multiple attenuation (0–7 dB) (Fig. 11, S39 and S40). Complexes 1-Co and 1-Cu show a linear dependence of the Rabi frequency, *Ω*_R_, on the microwave attenuation, consistent with the source of behavior as Rabi oscillations. Increased microwave attenuation leads to greater population transfer to the excited state resulting in larger amplitude and lower frequency Rabi oscillations. At 20 K, the inverse Rabi frequencies are 22–42 ns for complexes 1-Co and 1-Cu at 0–7 dB attenuation respectively. Fourier transform of the Rabi oscillations leads to analysis of the contributions to dephasing in each of the metal complexes. In the magnitude *vs.* frequency analysis (Fig. 11), there are clear contributions to dephasing from the hydrogen Larmour frequency (12.77 MHz at 300 mT), and hyperfine coupling to nitrogens of the tetradentate ligand.

## Discussion

Prediction of the spin–lattice relaxation rates for the two isostructural complexes 1-Co and 1-Cu based on first principles would suggest that the significantly higher free-ion spin–orbit coupling parameter of copper (829 *vs.* 528 cm^−1^) leads to faster spin–lattice relaxation *via* spin–vibration coupling. However, we find experimentally that the spin–lattice relaxation times are quite similar. Within the crystal field bonding model, this can be rationalized by increased metal–ligand bonding in copper *vs.* cobalt, which serves to decrease the effective spin–orbit coupling parameters, such that the reduced orbital parameter is effectively the same in the two complexes, leading to equivalent *T*_1_. This can be experimentally supported by the direct observation of d–d transitions and *g*-anisotropy, which allows direct estimation of the orbital reduction parameters. Here, it's important to note that the complexes are *S* = 1/2 where second-order spin–orbit coupling leads to *g*-anisotropy. Overall, enhanced metal–ligand covalency leads to reduction of the effective spin–orbit coupling parameter in 1-Cu relative to 1-Co, through a nephelauxetic effect, and in turn, enhanced spin–lattice relaxation times.

Metal–ligand covalency also has significant effects on decoherence times, here measured as phase memory times (*T*_m_). Major contributions in *T*_m_ values in 3d metal complexes arise from spin–spin interactions which include hyperfine interactions, dipolar interactions (relevant to the bath), and magnetic exchange (not present here). We find that 1-Cu has a longer coherence time than 1-Co, the difference of which increases at higher temperature. First principles would suggest shorter decoherence with increased hyperfine coupling,^79^ but we find that the coherence time in the copper complex is longer despite higher metal-based hyperfine coupling. The field dependent behavior of the relaxation supports this, in which the Brons–Van Vleck ratio (*e*/*f*) is smaller for copper (1-Cu) than cobalt (1-Co), suggesting lesser contributions from *B*_dip_ and *B*_hyp_ to spin relaxation. Analysis of the shape of the echo decay curves and FT Rabi oscillations suggest significant contributions from nuclear diffusion and restricted rotation of alkyl group in 1-Co as the source of the difference in decay times. Whereas in 1-Cu, the electron spin is more delocalized on the heteoaromatic rings in the *xy*-plane, in 1-Co, the spin is more heavily delocalized on the axial amine nitrogens, in which alkyl group hindered rotation dominates phase memory decay.

**Fig. 12:**
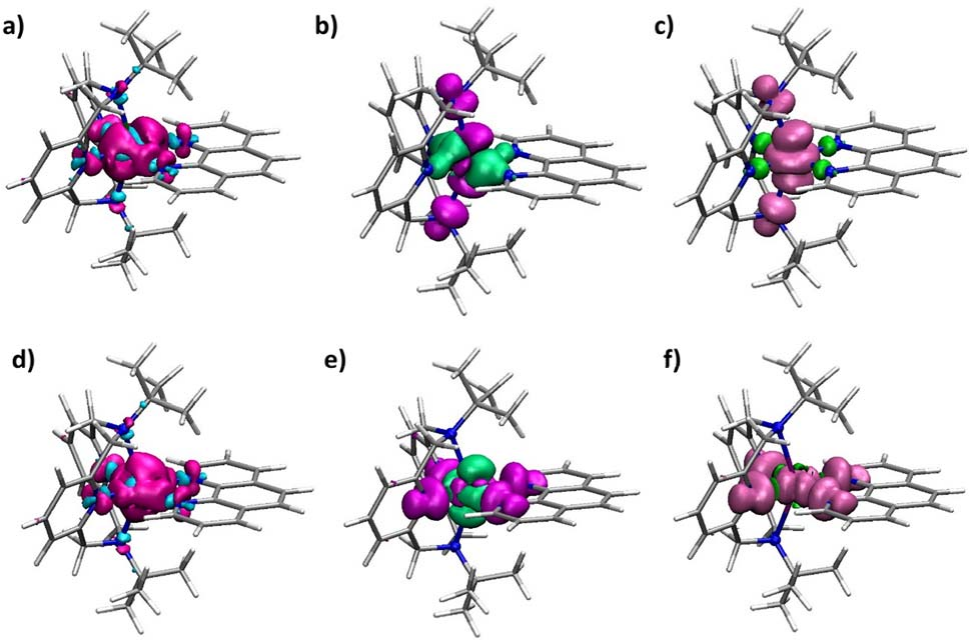
Electron density difference in 1-Co (a) and 1-Cu (d), spin density difference in 1-Co (b) and 1-Cu (e), and total spin density in 1-Co (c) and 1-Cu (f). Red, magenta, and pink correspond to increasing values (positive), and cyan and green correspond to decreasing values (negative).

In order to understand the contributions to spin relaxation in greater depth, electron density differences, spin density differences, and total spin densities were analyzed (Fig. 12). Electron density differences were calculated as the difference between the electron densities of the complex and the electron densities of the separate central atoms and the ligand. The results reveal an increase in electron density around the central metal-ion from the nitrogen atoms, consistent with the ligands functioning as N-donors to the central Co/Cu ion. Analysis of the spin densities suggests a considerable transfer of the spin density from the central metal ion to the axial nitrogens, consistent with the nature of the magnetic orbital on Co(ii) as the d_*z*^2^_ orbital. In the case of Cu(ii), consistent with the magnetic orbital as the d_*x*^2^−*y*^2^_, greater transfer occurs within the equatorial plane.

Modulations of the spin–orbit coupling dominate the mechanisms of spin–lattice relaxation, particularly, the Raman process and local mode process.^32^ Spin delocalization *via* metal–ligand bonding significantly reduces the effective spin–orbit coupling reducing the contribution of the Raman process, and in turn leading to slower spin–lattice relaxation. Further, the DFT calculations predict similar low-frequency vibrational modes for 1-Co and 1-Cu (Tables S26 and S27). The lowest frequency modes of 38.49 (1-Co) and 34.05 (1-Cu) cm^−1^ are shown in Fig. S41. The presence of similar low-frequency modes for both complexes is consistent with the same order of magnitude spin–lattice relaxation rates.

The magnetic relaxation in 1-Co and 1-Cu by means of the Raman process can be explained as follows. In the zero field, the electronic states of the odd-electron system 1, mixed by the spin–orbit coupling, form a manifold of spin–orbit Kramer doublets, *i.e.*, effective spin-1/2 states. In the applied external magnetic field, each doublet state is split into two components with different orientations along the field.

**Fig. 13:**
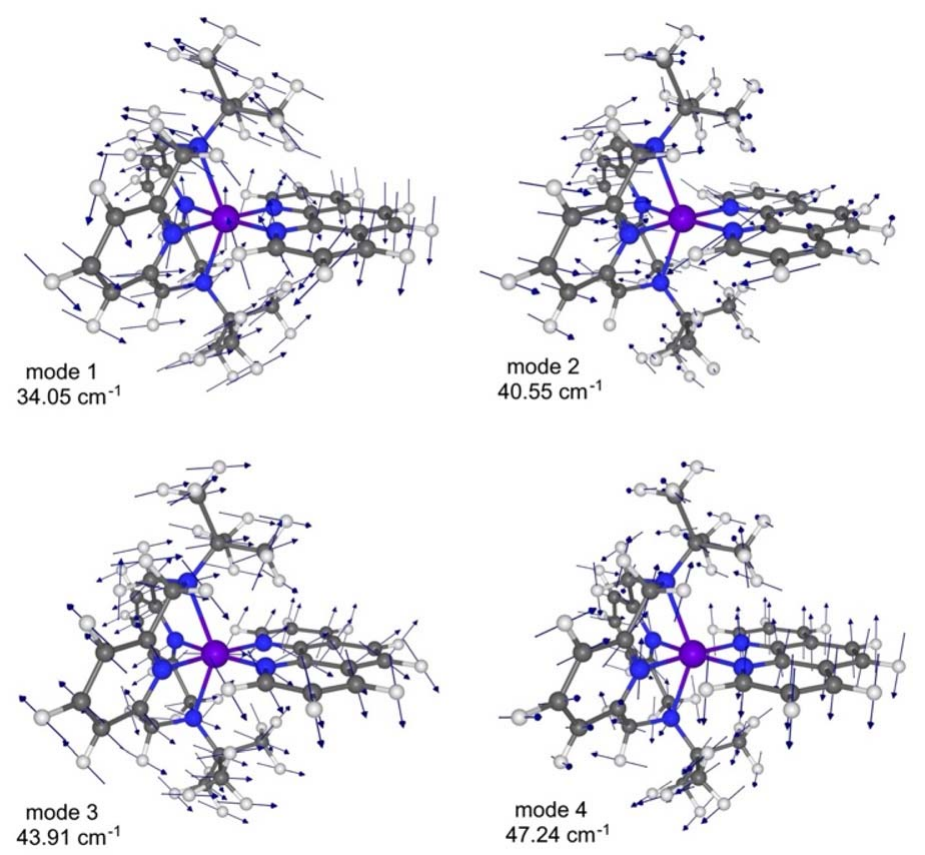
The lowest frequency vibrational mode (mode 1), and quasi-degenerate vibrational modes (modes 2, 3, and 4) for 1-Cu proposed as candidate modes responsible for Raman relaxation.

The traditional Raman mechanism (Van Vleck relaxation) requires scattering of a phonon between two different, nearly degenerate vibronic states to satisfy energy conservation, with a T^9^ temperature dependence. Alternatively, as is often observed for *S* = 1/2 spin systems, a two-phonon Raman process driven by an anharmonic localized molecular mode can occur, with a temperature dependence described by a power law (T^3^–T^5^).^73,109^ The magnitude of the Zeeman splitting between components of the ground spin state in 1-Co and 1-Cu is estimated from constructing and diagonalizing the Zeeman Hamiltonian, where the experimental *g*_*x*_, *g*_*y*_, and *g*_*z*_ values and the field of 5000 Oe were used in the calculations. The average splitting of 0.50 cm^−1^ was calculated at different orientations of the magnetic field relative to the principal axes of the molecular *g*-tensor, *i.e.*, using different direction cosines (Table S28). Therefore, the nearly degenerate vibrations, *i.e.*, pairs of vibrations with Δ*ω* = 0.50 cm^−1^ give the largest contribution to the Raman relaxation. In the temperature range of 20–100 K, where the Raman process either dominates or has significant contribution to the magnetic relaxation, the candidate vibrations can be either acoustic or optical phonons of the lattice, or the low-frequency molecular vibrations. Taking into account both the finite linewidth of the vibrational transitions and the above temperature range, we identify a total of six candidate molecular vibrations below 100 cm^−1^ (Fig. 13 and Table S15).

The decomposition analysis of these vibrations is given in Table S26 (1-Co) and Table S27 (1-Cu). Animations of the vibrations can be found as gif files in the SI. The first three vibrations are degenerate within 6 cm^−1^ and have the harmonic frequencies of 47, 49, and 51 cm^−1^ for 1-Co, and 41, 44, and 47 cm^−1^ for 1-Cu. These vibrations correspond to the N(Py)–Co/Cu–N(Phen) and N(Py)–Co/Cu–N(Py) angles bending, and to twisting of the N(Phen)–Co/Cu–N(Phen) angle. The second three vibrations are degenerate within 2 cm^−1^, and have the frequencies of 71, 73, and 73 cm^−1^ for 1-Co, and 69, 70, and 71 cm^−1^ for 1-Cu. One of these vibrations corresponds to a bending of the N(amino)–Co/Cu–N(amino) angle which causes a significant displacement of the *tert*-butyl groups. The other two vibrations correspond to symmetrically equivalent rotations of the *tert*-butyl groups around the N–C bonds and are accompanied by the slight rotation of CoN_6_ center octahedron. The above discussion exemplifies the strong magneto-structural correlation in the spin-1/2 systems. Ideally, in the high symmetry *O*_h_ and *T*_h_ point groups, the Raman relaxation would be suppressed by avoiding the splitting between the degenerate vibrations of the *E* and *T* symmetries. In practice, when the splitting is induced by distortion from the high symmetry, the interconnection between the Raman relaxation rate, molecular symmetry, and the magnitude of the Zeeman splitting can be studied systematically *via* a rational ligand design. The results here suggest that the dominant contributions to the Raman I process of spin relaxation in *ls*-Co(ii) and Cu(ii) complexes are consistent with reduced spin–orbit coupling due to metal–ligand covalency. As spin relaxation processes have both adiabatic (modulation of the *g*-value) and nonadiabatic contributions, our analysis is consistent with Raman process arising from spin–vibrational coupling of nearly degenerate vibrational modes, which typically operates in mid-range temperatures (15 < *T* < 60K). This analysis is consistent with recent results predicted by Shuskhkov, *et al.* for nonadiabatic contributions to spin relaxation processes in isolated doublet states.^45^ Lastly, if one considers two-phonon Raman contributions to *T*_1_ and *T*_2_, there is a contribution to 
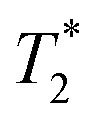
 which vanishes with one-phonon processes in which the two-phonon process creates magnetic noise and spectral diffusion, causing dephasing. Thus, the two-phonon Raman process can induce both *T*_1_ and *T*_2_ relaxation, giving *T*_2_ ∼ *T*_1_, or *T*_2_ ∼ 0.8 *T*_1_.^109^ As we have identified Raman processes through local vibrational modes as a probable contributor, this process may also contribute to the observed difference in *T*_m_ between the 1-Co and 1-Cu complexes.

## Conclusions

We investigated the spin dynamics of two isostructural complexes in which the spin–orbit coupling is modulated between a *ls*-Co(ii) (*S* = 1/2) and Cu(ii) (*S* = 1/2) complex. Structural analysis though SC-XRD reveals isostructural geometries in the two complexes. Electronic structure analysis through electronic absorption spectroscopy and computation suggests a decrease in effective spin–orbit coupling due to metal–ligand covalency in the Cu(ii) relative to the Co(ii) complex. Analysis of spin dynamics through AC susceptibility measurements (in a polycrystalline state) and pulsed EPR (in a solvent matrix) indicate that the two complexes exhibit similar rates of spin–lattice relaxation (*T*_1_) as a function of temperature. Here, spin–lattice relaxation are dominated by Raman-I processes, in which the dominant pathway for relaxation is nonadiabatic coupling through nearly degenerate low-frequency modes of the MN_6_ core. The contribution of the Raman(I) process is proportional to the spin–orbit coupling parameter, suggesting the importance of metal–ligand covalency. The decoherence (*T*_2_) times for both complexes are relatively prolonged, with cobalt showing a shorter decoherence time and greater temperature dependence compared to copper. Metal–ligand covalency induces directional spin-delocalization into the ligands, inducing spin-polarization on the rigid heteroaromatic ligands for Cu(ii), and *tert*-butyl amine ligands for *ls*-Co(ii). The findings provide fundamental insights into the impact of quasi-degenerate vibrational modes and metal–ligand covalency on spin dynamics, and suggest that increasing metal–ligand covalency is an important strategy for reducing effective spin–orbit coupling, spin–lattice relaxation and decoherence rates in molecular qubit platforms for quantum information science.

## Author contributions

Conceptualization (NLF, SG), funding acquisition (NLF, SAV), experimental investigation (SG), EPR (PHO and SG), computations (SAV, MF, VDD), methodology (NLF, SG, PHO, SAV), project administration (NLF), software (SG, VDD, MF), supervision (NLF), writing of the original draft (NLF, SG), all coauthors contributed to review and editing of the manuscript.

## Conflicts of interest

There are no conflicts to declare.

## Supplementary Material

SC-017-D5SC09844K-s001

SC-017-D5SC09844K-s002

## Data Availability

Supplementary information (SI): experimental details, crystal structure investigations, physical methods, magnetic and spectroscopic studies, and theoretical calculations have been reported in the SI (PDF). See DOI: https://doi.org/10.1039/d5sc09844k. CCDC 2320669–2320671 contain the supplementary crystallographic data for this paper.^110*a–c*^
