# Supplementary material for: Quantum coherence enhancement through control of metal–ligand covalency: modulating spin–orbit coupling in isostructural molecular qubits
Source: Chem Sci. 2026 Mar 30;17(20):10029–46. doi: 10.1039/d5sc09844k (PMC13054892; doi:10.1039/d5sc09844k)
Supplement: SC-017-D5SC09844K-s001 [file SC-017-D5SC09844K-s001.pdf]

Supplementary Information (SI) for Chemical Science.
This journal is © The Royal Society of Chemistry 2026

Supplementary Information

Quantum Coherence Enhancement Through Control of Metal-Ligand Covalency: Modulating Spin-Orbit Coupling in Isostructural Complexes

Subrata Ghosh,[1] Paul H. Oyala,[2] Maksym Fizer,[1] Vsevolod D. Dergachev,[1] Sergey A. Varganov,[1] and Natia L. Frank[1,*]

[1]Department of Chemistry, University of Nevada, Reno, 1644 N. Virginia St., NV 89557, USA.

[2]Division of Chemistry and Chemical Engineering, California Institute of Technology, Pasadena, California 91125, USA.

KEYWORDS. Quantum information science, molecular qubit, spin, transition-metal, copper, cobalt, spin-orbit coupling, spin-lattice relaxation, decoherence.

Table of Contents:

1. Experimental section S9
 1.1 Materials and physical measurements S9
 1.2 Synthesis of 1-Co and 1-Cu S10
 1.3 X-ray crystallography S11
 1.4 Magnetic measurements S11
 1.5 EPR measurements S13
 1.6 Electronic structure calculations....................S18
2. Data....................S22
 2.1 Characterization of 1-Co and 1-Cu S22
 2.2 X-ray crystallography S26
 2.3 Magnetic measurements S32
 2.4 Electronic absorption spectroscopy S42
 2.5 EPR measurements S44
 2.6. Electronic structure calculations....................S65
3. References....................S81

List of Figures and Tables

2.1 Characterization of 1-Co and 1-Cu

Figure S1 TGA plots for **1-Co** and **1-Cu**.

Figure S2 IR spectra of complexes **1-Co** and **1-Cu** at 300 K.

Figure S3 ESI mass spectrum of **1-Co** in acetonitrile: toluene (1:1) at 300 K.

Figure S4 Isotope distribution for $[Co(^{t}Bu\text{-}N4)(Phen)]^{2+}$ (experimental, top; and calculated, bottom) in **1-Co** in acetonitrile: toluene (1:1) at 300 K.

Figure S5 ESI mass spectrum of **1-Cu** in acetonitrile: toluene (1:1) at 300 K.

Figure S6 Isotope distribution for $[Cu(^{t}Bu\text{-}N4)(Phen)]^{2+}$ (experimental, top; and calculated, bottom) in **1-Cu** in acetonitrile: toluene (1:1) at 300 K.

2.2 XRD

Figure S7 Top: Single crystal X-ray structure of complex cation in **1-Co** (a) and **1-Cu** (b) at 100 K. Hydrogen atoms and counter anions are omitted for clarity (Co, green; Cu, purple; C, gray; N, blue). Bottom: Unit cell packing diagram of **1-Co** (c) and **1-Cu** (d). Hydrogen atoms are omitted for clarity. (Co, green; Cu, purple; C, gray; N, blue; B, light pink; F, light-green).

Figure S8 Unit cell packing diagram of **1-Co** at 296 K. Hydrogen atoms are omitted for clarity. (Co: green, C: grey, N: blue; B: light-pink; F: light-green.

Table S1 X-ray crystallography data for complexes **1-Co** and **1-Cu**.

Table S2 Selected bond distances (Å) and bond angles in **1-Co** at 100 and 296 K.

Table S3 Selected bond distances (Å) and bond angles in **1-Cu**.

Table S4 CShM analysis data for complexes **1-Co** and **1-Cu**.

Table S5 Octahedral distortion parameters for complexes **1-Co** and **1-Cu**.

Figure S9 Comparison of the 300 K experimental PXRD pattern and simulation (296 K) for **1-Co**.

Figure S10 Comparison of the 300 K experimental PXRD pattern and the simulation (100 K) for **1-Cu**.

2.3 Magnetic measurements

Figure S11 Temperature dependence of χT for **1-Co** and **1-Cu** at 1000 Oe

Figure S12 Field dependence of the magnetization as M vs H plots for **1-Co** (left) and **1-Cu** (right) at 100 K. The solid lines are the best fit.

Figure S13 Top: Field dependence of the magnetization as *M vs H* (left) and *M vs H/T* (right) plots for **1-Co** at 2, 3, 5, and 8 K. The solid lines are a guide for the eyes. Bottom: Brillouin function fit (red line) to the *M vs H/T* at 2 K.

Figure S14 Top: Field dependence of the magnetization as *M vs H* (left) and *M vs H/T* (right) plots for **1-Cu** at at 2, 3, 5, and 8 K. The solid lines are a guide for the eyes. Bottom: Brillouin function fit (red line) to the *M vs H/T* at 2 K.

Figure S15 Frequency dependence of the real (χ', left) and imaginary (χ'', right) components of the AC susceptibility at different AC frequencies from 1 - 1000 Hz under 2500 Oe external DC field, with a 3 Oe AC field for **1-Co** (a and b) **1-Cu** (c and d) at different temperatures. Full lines correspond to fit with generalized Debye model.

Figure S16 Top: Frequency dependence of the real (χ', left) and imaginary (χ'', right) components of the ac susceptibility at different ac frequencies from 1 - 1000 Hz different external dc field from 0 – 6000 Oe, respectively with a 3 Oe ac field for **1-Co** at 2 K. Full lines correspond to fit with Generalized Debye model. Bottom: The Cole-Cole plot with Generalized Debye fit.

Figure S17 Frequency dependence of the real (c', top) and imaginary (c'', middle) components of the ac susceptibility at different ac frequencies from 1 - 1000 Hz different external dc field from 100 – 6000 Oe, respectively with a 3 Oe ac field for **1-Cu** at 2 K. Full lines correspond to fit with Generalized Debye model. Bottom: The Cole-Cole plot with Generalized Debye fit.

Table S6 Fitting parameters used in the generalized Debye model for variable temperature AC susceptibility data collect of **1-Co** at 2500 Oe.

Table S7 Fitting parameters used in the generalized Debye model for variable field AC susceptibility data collect of **1-Co** at 2 K.

Table S8 Fitting parameters used in the generalized Debye model for variable temperature AC susceptibility data collect of **1-Cu** at 2500 Oe.

Table S9 Fitting parameters used in the generalized Debye model for variable field AC susceptibility data collect of **1-Cu** at 2 K.

2.4 Electronic absorption spectroscopy

Figure S18 UV-vis spectra of **1-Co** and **1-Cu** in acetonitrile: toluene (1:1) at 300 K.

Figure S19 UV-vis-NIR spectrum of **1-Co** in 1 mM acetonitrile: toluene (1:1) solution at 300 K (a) along with the deconvoluted spectra (b and c).

Figure S20 UV-vis-NIR spectrum of **1-Cu** in 1 mM acetonitrile: toluene (1:1) solution at 300 K (a) along with deconvoluted spectra (b).

Figure S21 The NEVPT2\CASSCF calculated absorption spectrum of **1-Co** (left) and **1-Cu** (right) arising from electronic transitions $^2A_{1g} \rightarrow {}^2E_g$ (10815 cm^{-1}), $^2A_{1g} \rightarrow {}^2B_{1g}$ (12874 cm^{-1}), and $^2A_{1g} \rightarrow {}^2A_{2g}$ (19017 cm^{-1}) for **1-Co** and from $^2B_{1g} \rightarrow {}^2A_{1g}$ (10656 cm^{-1}), $^2B_{1g} \rightarrow {}^2B_{2g}$ (15315 cm^{-1}), and $^2B_{1g} \rightarrow {}^2E_g$ (16654 cm^{-1}) for **1-Cu**.

2.5 EPR measurements

Figure S22 X-band CW-EPR spectra of **1-Co** (left) and **1-Cu** (right), with simulations overlaid in red. Acquisition parameters: X-band CW-EPR: temperature = 77 K (LN$_2$), MW frequency = 9.390 GHz, MW power = 550 µW, modulation amplitude = 0.8 mT, conversion time = 10 ms.

Figure S23 Q-band Davies ENDOR spectra of **1-Co,** with total simulations overlaid in red, and individual nuclear contributions plotted beneath in cyan (^{59}Co) and green (^{14}N$_1$). Asterisks indicate signals arising from 3rd harmonic of intense ^{59}Co signals at higher frequency – these are absent in the low frequency ENDOR because a low-pass RF filter with a cut-off frequency of 35 MHz was used for these spectra. Sharp, non-simulated signals centered around c.a. 50-52 MHz are from weakly coupled ^{1}H nuclei of ligand. Acquisition parameters: Low Freq. ENDOR: τ = 400 ns, MW π pulse length = 160 ns, RF π pulse length = 40 µs. Wide ENDOR spectra: τ = 240 ns, MW π pulse length = 160 ns, RF π pulse length = 40 µs. Both ENDOR data sets: temperature = 7.5 K, MW frequency = 34.133 GHz, T_{RF} delay = 2 µs, shot rep. time = 20 ms.

Figure S24 Q-band Davies ENDOR spectra of **1-Cu** with total simulations overlaid in red, and individual nuclear contributions plotted beneath in cyan ($^{63/65}$Cu), green (^{14}N$_2$), and blue (^{14}N$_3$). Unsimulated signals centered around c.a. 50-52 MHz in wider ENDOR spectra are from weakly coupled ^{1}H nuclei of ligand. Acquisition parameters: Low Freq. ENDOR: τ = 400 ns, MW π pulse length = 160 ns, RF π pulse length = 40 µs. Wide ENDOR spectra: τ = 240 ns, MW π pulse length = 160 ns, RF π pulse length = 40 µs. Both ENDOR data sets: temperature = 10 K, MW frequency = 34.125 GHz, T_{RF} delay = 2 µs, shot rep. time = 20 ms.

Figure S25 Echo-detected field-swept spectra recorded at the X-band (9.714 GHz) for frozen solutions (ca. 1 mM) of **1-Co** (left) and **1-Cu** (right) in acetonitrile-d$_3$: toluene-d$_8$ (1:1) at 10 K and 20 K respectively.

Figure S26. Variable-temperature inversion recovery curves for frozen solution (ca. 1 mM) of **1-Co** in acetonitrile-d$_3$: toluene-d$_8$ (1:1) measured at 313.4 mT. Red solid lines represent the best fits to the exponential decays using a stretched monoexponential equation.

Figure S27 Variable-temperature inversion recovery curves for frozen solution (ca. 1 mM) of **1-Co** in acetonitrile-d$_3$: toluene-d$_8$ (1:1) measured at 341.5 mT. Red solid lines represent the best fits to the exponential decays using a stretched monoexponential equation.

Figure S28 Variable-temperature inversion recovery curves for frozen solution (ca. 1 mM) of **1-Cu** in acetonitrile-d_3: toluene-d_8 (1:1) measured at 312.7 mT. Red solid lines represent the best fits to the exponential decays using a stretched monoexponential equation.

Figure S29 Variable-temperature inversion recovery curves for frozen solution (ca. 1 mM) of **1-Cu** in acetonitrile-d_3: toluene-d_8 (1:1) measured at 340.28 mT. Red solid lines represent the best fits to the exponential decays using a stretched monoexponential equation.

Figure S30 Temperature dependence of electron spin relaxation time: spin–lattice relaxation (T_1) for complex **1-Co** at 313.4 mT and 341.5 mT in acetonitrile-d_3: toluene-d_8 (1:1). Full lines are the best fits of the models with the combination of direct and Raman processes.

Figure S31 Temperature dependence of electron spin relaxation time: spin–lattice relaxation (T_1) for complex **1-Cu** at 312.7 mT and 340.28 mT in acetonitrile-d_3: toluene-d_8 (1:1). Full lines are the best fits of the models with the combination of direct and Raman processes.

Figure S32 Variable-temperature Hahn echo decay curves for frozen solution (ca. 1 mM) of **1-Co** in acetonitrile-d_3: toluene-d_8 (1:1) measured at 313.4 mT. Red solid lines represent the best fits to the exponential decays using a stretched monoexponential equation.

Figure S33 Variable-temperature Hahn echo decay curves for frozen solution (ca. 1 mM) of **1-Co** in acetonitrile-d_3: toluene-d_8 (1:1) measured at 341.5 mT. Red solid lines represent the best fits to the exponential decays using a stretched monoexponential equation.

Figure S34 Variable-temperature Hahn echo decay curves for frozen solution (ca. 1 mM) of **1-Cu** in acetonitrile-d_3: toluene-d_8 (1:1) measured at 312.7 mT. Red solid lines represent the best fits to the exponential decays using a stretched monoexponential equation.

Figure S35 Variable-temperature Hahn echo decay curves for frozen solution (ca. 1 mM) of **1-Cu** in acetonitrile-d_3: toluene-d_8 (1:1) measured at 340.28 mT. Red solid lines represent the best fits to the exponential decays using a stretched monoexponential equation.

Table S10 Temperature-dependent T_1 and T_m data collected at selected field positions at X-band for **1-Co** in acetonitrile-d_3: toluene-d_8 (1:1).

Table S11 Temperature-dependent T_1 and T_m data collected at selected field positions at X-band for complex **1-Cu** in acetonitrile-d_3: toluene-d_8 (1:1).

Table S12 Temperature-dependent β_1 and β_m data collected at selected field positions at X-band for complex **1-Co** in acetonitrile-d_3: toluene-d_8 (1:1).

Table S13 Temperature-dependent β_1 and β_m data collected at selected field positions at X-band for complex **1-Cu** in acetonitrile-d_3: toluene-d_8 (1:1).

Table S14 Best-Fit Parameters to Reproduce the Temperature Dependence of the Spin−Lattice Relaxation Rate for **1-Co** and **1-Cu**.

Figure S36 (a) Total spin density (calculated using the B3PW91/EPR-II+CP(PPP) method (*see* ESI for details), (b) Temperature dependence of spin–lattice relaxation (T_1) for complex **1-Co** at 341.5 mT ($g_{\|}$) and **1-Cu** at 340.28 mT ($g_{\perp}$) in acetonitrile-d_3: toluene-d_8 (1:1), full lines are the best fits of the models with the combination of direct and Raman processes, (c) T_1 anisotropy for **1-Co** and (d) T_1 anisotropy for **1-Cu** in a dilute solvent matrix.

Figure S37 Temperature dependence of spin–lattice relaxation. Log–log plots of the spin–lattice relaxation rate ($1/T_1$) versus temperature for complexes **1-Co** (blue) and **1-Cu** (maroon) measured at the indicated magnetic fields in acetonitrile-d_3:toluene-d_8 (1:1).

Figure S38 Temperature dependence of phase memory time. Log–log plots of the phase memory relaxation rate ($1/T_m$) as a function of temperature for complexes **1-Co** (blue) and **1-Cu** (maroon) measured at the indicated magnetic fields in acetonitrile-d_3:toluene-d_8 (1:1). a) Field-dependent measurements at multiple magnetic field positions. b) Representative data at selected fields.

Figure S39 Top: Rabi oscillations for complex **1-Co** in acetonitrile-d_3: toluene-d_8 (1:1) were recorded at 20 K for different microwave attenuations (0 – 7 dB) at 341.5 mT. Bottom: Fourier transform of the Rabi oscillations (left) and linear dependence of the Rabi frequency (Ω_R) as a function of the relative microwave attenuation B_1. The relative microwave attenuation is calculated relative to the weakest microwave power examined (7 dB).

Figure S40 Top: Rabi oscillations for complex **1-Cu** in acetonitrile-d_3: toluene-d_8 (1:1) were recorded at 20 K for different microwave attenuations (0 – 7 dB) at 312.7 mT. Bottom: Fourier transform of the Rabi oscillations (left) and linear dependence of the Rabi frequency (Ω_R) as a function of the relative microwave attenuation B_1. The relative microwave attenuation is calculated relative to the weakest microwave power examined (7 dB).

2.6 Electronic structure calculations

Figure S41 The lowest frequency vibrational modes: (a) **1-Co** (38.49 cm^{-1}), (b) **1-Cu** (34.05 cm^{-1}). The arrows are showing relative displacements of atoms.

Table S15 Selected vibrational modes.

Table S16 Energies (cm^{-1}) and symmetry (Γ) of the ligand field one-electron eigenfunctions (orbitals) calculated by the *ab initio* ligand field theory (AILFT)[28] using the CASSCF (7,5) calculations for **1-Co** and the CASSCF(9,5) calculations for **1-Cu**. The orbital symmetry is given in the approximate D_{4h} symmetry of the complex.

Table S17 Dominant configurations, symmetries, and NEVPT2 energies (cm^{-1}) of the thirteen lowest-energy electronic states of **1-Co** which arise from the d^7 electronic configuration of cobalt (II), oscillator strengths for the transitions from the ground doublet state, and reduced SOC matrix elements (cm^{-1}) between

the ground state and each of the excited states. Configurations are written as particular occupations of the five active space orbitals. In a short notation, only a singly occupied and unoccupied orbitals are shown. The active space orbitals are composed dominantly of the atomic d orbitals of Co. The symmetries of the states are given in the approximate D_{4h} symmetry of the complex. The e_g and e'_g orbitals correspond to two linear combinations of the d_{xz} and d_{yz} orbitals. Note that the actual C_1 symmetry results in breaking of the degeneracy of the E states.

Table S18 Dominant configurations, symmetries, and NEVPT2 energies (cm^{-1}) of the electronic states of **1-Cu,** which arise from the d^9 electronic configuration of copper (II), oscillator strengths for the transitions from the ground doublet state, and reduced SOC matrix elements (cm^{-1}) between the ground state and each of the excited states. Configurations are written as particular occupations of the five active space orbitals. In a short notation, only a singly occupied orbital is shown. The active space orbitals are composed dominantly of the atomic d orbitals of Cu. The e_g and e'_g orbitals correspond to two linear combinations of the d_{xz} and d_{yz} orbitals.

Table S19 Symmetry of the orbital part of the spin-orbit coupling between the ground electronic state of **1-Co** (**1-Cu**) and various excited states in the approximate D_{4h} point group. Symbols Γ, Γ^L, and Γ' denote symmetry of the ground state, the angular momentum operator $L = [(L_x, L_y), L_z]$, and an excited state. The orbital part (matrix element) is zero if the symmetry product does not contain the totally symmetric irreducible representation, a_{1g}. The a_{1g} representation is highlighted by an underscore when present in the symmetry product.

Table S20 Principal components of the *g*-tensor of **1-Co** calculated using different number of the excited states included in the state-averaging. The shifts Δ*g* are reported relative to the free-electron *g*-value. The cases correspond to cumulative inclusion of the excited states. Each case is different from the previous by inclusion of the next nearest excited state or a group of close-lying states (see Table S16). Case 1 corresponds to 3 quartet states, case 2 – to 4 quartets, case 3 – to 3 doublets and 4 quartets, case 4 – to 4 doublets and 4 quartets, case 5 – to 4 doublets and 6 quartets, case 6 – to 5 doublets and 6 quartets, case 7 – to 7 doubles and 6 quartets, and case 8 – to all 40 doublet and 10 quartet states that arise from the d^7 configuration of Co(II).

Table S21 Principal components of the *g*-tensor of **1-Cu** calculated using different number of the excited states included in the state-averaging. The shifts Δ*g* are reported relative to the free-electron *g*-value. The cases correspond to inclusion of the first excited state (A_{1g}), two first excited states (A_{1g} and B_{2g}), and all the excited states (A_{1g}, B_{2g}, and E_g).

Table S22 Hyperfine coupling constants A_{xx}, A_{yy}, and A_{zz} (MHz) of metals in **1-Co** and **1-Cu** cations calculated at various levels of theory. Geometries of **1-Co** and **1-Cu** cations were preoptimized at the *w*B97M-D3BJ/def2-SVP level of theory.

Table S23 DFT calculated Mulliken spin density of the unpaired electron spin on the metal and the directly bound donor nitrogen atoms in complexes **1-Co** and **1-Cu**.

Table S24 Combined experimental and DFT predicted hyperfine parameters for **1-Co** and **1-Cu.**

Table S25 Combined experimental and DFT predicted nuclear quadrupole coupling parameters for **1-Co** and **1-Cu.**

Table S26 The first twenty vibrational modes with frequencies (ν, cm^{-1}), intensities (I, km × mol^{-1}), and types of vibrations [bond stretching (BOND), angle bending (ANG), torsion angle vibrations (TOR)] calculated for **1-Co**.

Table S27 The first twenty vibrational modes with frequencies (ν, cm^{-1}), intensities (I, km × mol^{-1}), and types of vibrations [bond stretching (BOND), angle bending (ANG), torsion angle vibrations (TOR)] calculated for **1-Cu**.

Table S28 Magnitude of the Zeeman splitting (in cm^{-1}) calculated for **1-Co** and **1-Cu** at different orientations of the magnetic field of 5000 Oe (0.5 T) relative to the principal axes of the molecular g-tensor.

1. Experimental section

1.1 Materials and physical measurements

All manipulations were carried out under an argon atmosphere using standard Schlenk techniques unless otherwise stated. Solvents were dried by standard methods and were freshly distilled before to use. All chemicals were used as purchased from the chemical sources without further purification. The macrocyclic ligand *N*,*N*'-di-tert-butyl-2,11-diaza[3,3](2,6) pyridinophane (^{t}Bu-N4**)** and [M(^{t}Bu-N4)Cl_2] (M = Co(II) and Cu(II)) were synthesized according to the literature procedure.[1-2] The crystals of both complexes were removed from the mother liquor and dried on a filter paper to remove any adhering solvent molecules, prior to measurement. The elemental analyses for C, H, and N were performed with a Eurovector EA 3000 (Pavia, Italy) elemental analyzer, using acetanilide and benzoic acid calibration standards. ATR-IR spectra of solids were recorded in the range of 4000–400 cm^{-1} on a Thermo Nexus 470 FT-IR. For each spectrum an atmospheric background was subtracted. Complexes **1-Co** and **1-Cu** are cationic, with BF_4^- as counter anions. The stretching vibrations of BF_4^- are found at around in the range between 1100 - 1050 cm^{-1} in both complexes (Figure S4). Powder X-ray diffraction (PXRD) measurements were carried out on a Bruker D8 diffractometer at 40 kV and 40 mA, under Cu-K*a* radiation (λ = 1.54059 Å) at 300 K. PXRD data analyses were done using DIFFRAC.EVA software. PXRD patterns were compared with the simulated patterns obtained from the single-crystal X-ray diffraction (SC-XRD) data using Mercury 4.2.0.[6] Thermogravimetric analysis (TGA) was done on a Q50 TGA analyzer with a heating rate of 10 K min^{-1} under a nitrogen atmosphere. UV/vis/NIR spectra were carried out on a Shimadzu UV-2550 spectrometer in solution in quartz cuvettes with a path length of 1 cm. UV-vis-NIR spectrum of **1-Cu** was deconvoluted with Lorentz model using Origin. Electrospray Ionization Time-of-Flight mass spectrometry (ESI-TOF MS) in a mixture of acetonitrile and toluene (1:1) at 300 K were consistent with [Co(^{t}Bu-N4)(Phen)]$^{2+}$ for **1-Co** and

[Cu(^{t}Bu-N4)(Phen)]$^{2+}$ for **1-Cu** in solutions used for both Evan's method measurements, electronic absorption spectroscopy, and pulsed EPR measurements.

1.2 Synthesis of 1-Co and 1-Cu

[Co(^{t}Bu-N4)(Phen)](BF_4)$_2$ (**1-Co**). Under an argon atmosphere, to a solution of [Co(^{t}Bu-N4)Cl_2] (25 mg, 0.05 mmol) in a mixture of methanol and dichloromethane (1/1) (7 mL) was added a solution of phen (10 mg, 0.05 mmol) in the same mixture of solvents (7 mL). After stirring for 4 h, a solution of $NaBF_4$ (11 mg, 0.1 mmol) in the same solvent mixture (4 mL) was added to the above reaction mixture. After stirring for an additional 2 h, the resulting reaction mixture was filtered, and diethyl ether was slowly diffused into the filtrate to obtain **1-Co** as red crystals in 55% yield. Data for **1-Co**, $C_{34}H_{40}B_2F_8N_6Co$ (M.W.: 765.27 g mol^{-1}) as follows. Anal. Calcd: C, 53.36; H, 5.27; N, 10.98. Found: C, 53.06; H, 5.26; N, 10.70. ATR-IR (only intense bands): *n* (cm^{-1}) 1600, 1522, 1469, 1426, 1224, 1168, 1036, 1013, 937, 907, 841, 799, 781, 768, 726, 710. ESI-TOF (MS) *m/z* for [Co(^{t}Bu-N4)(Phen)]$^{2+}$, Calcd. 295.6317; found: 295.6322.

[Cu(^{t}Bu-N4)(Phen)](BF_4)$_2$ (**1-Cu**). Under an argon atmosphere, to a solution of [Cu(^{t}Bu-N4)Cl_2] (25 mg, 0.05 mmol) in a mixture of methanol and dichloromethane (1/1) (7 mL) was added a solution of phen (10 mg, 0.05 mmol) in the same mixture of solvents (7 mL). After stirring for 4 h, a solution of $NaBF_4$ (11 mg, 0.1 mmol) in the same solvent mixture (4 mL) was added to the above reaction mixture. After stirring for an additional 2 h, the resulting reaction mixture was filtered, and diethyl ether was slowly diffused into the filtrate to obtain **1-Cu** as pale-blue crystals in 76% yield. Data for **1-Cu**, $C_{34}H_{40}B_2CuF_8N_6$ (M.W.: 769.89 g mol^{-1}) are as follows. Anal. Calcd: C, 53.04; H, 5.24; N, 10.92. Found: C, 53.06; H, 4.84; N, 10.80. ATR-IR (only intense bands): *n* (cm^{-1}) 2981, 1599, 1523, 1465, 1428, 1403, 1295, 1263, 1224, 1147, 1047, 959, 937, 922, 800, 778, 769, 735, 721, 710. ESI-TOF (MS) m/z for [Cu(^{t}Bu-N4)(Phen)]$^{2+}$, Calcd. 297.6301; found: 297.6300.

1.3 X-ray crystallography

Single crystal X-ray structure analysis of complexes **1-Co** and **1-Cu** were collected with a Bruker SMART APEX CCD diffractometer equipped with graphite monochromated MoKa radiation (l = 0.71073 Å). The single crystal was mounted on a crystal mounting loop with the help of Paratone oil purchased from MiTeGen at 296 K and then slowly cooled down to 100 K with 4 K/min ramping rate using a liquid nitrogen gas stream cooling device, followed by data collection at respective temperatures. Data integration and reduction were carried out using SAINT software, and empirical absorption corrections were performed using the SADABS program.[3] Structures were solved using direct methods and refined with a full-matrix least-squares method on F^2 using SHELXL-2018 included in Olex2 version 1.5.[5] The packing diagrams were made using Mercury 4.2.0.[6] All other non-hydrogen atoms of complexes **1-Co** and **1-Cu** were refined anisotropically and hydrogen atoms were labeled to ideal positions and refined isotropically using a riding model. CCDC 2320669, 2320670, and 2320671 contain the supplementary crystallographic data for this paper. These data can be obtained free of charge from The Cambridge Crystallographic Data Centre via www.ccdc.cam.ac.uk/data_request/cif.

1.4 Magnetic measurements

DC magnetic measurements were carried out with a Quantum Design MPMS3 SQUID magnetometer from 2 – 300 K under an external dc applied fields in the range of -7 T to 7 T. A polycrystalline sample of **1-Co** (18.10 mg) and **1-Cu** (14.17 mg) was measured in a AG1 capsule. The temperature dependence of magnetization was measured with an external dc field of 1000 Oe. The isothermal magnetization was studied at 2, 3, 5, and 8 K. *M vs H* data recorded at 100 K to determine the presence of ferromagnetic impurities led to linear behaviour consistent with no ferromagnetic impurities. The magnetic susceptibility was corrected for with diamagnetic

susceptibility of both the sample holder (point-by-point experimental) and the sample ($\chi_{\rm dia} = -\frac{MW}{2} \times 10^{-6}$ cm^3 mol^{-1})[4]. M vs H/T data at 2 K were fitted with Brillouin function

$$M = NgJm_B B_j(x)$$

$$B_j(x) = \frac{2S+1}{2S}\coth\left(\frac{2S+1}{2S}x\right) - \frac{1}{2S}\coth\left(\frac{x}{2S}\right);\ x = \frac{gJm_B B}{k_B T}$$

AC susceptibility measurements were carried out with an oscillating AC field of 3 Oe with a frequency between 0.1 to 1000 Hz. Collected sets of χ' and χ'' at each temperature were fitted using the formulas for extended one-set Debye model[5]

$$\chi'(\omega) = \chi_S + (\chi_T - \chi_S)\frac{1 + (\omega\tau)^{(1-\alpha)}\sin\left(\frac{\pi\alpha}{2}\right)}{1 + 2(\omega\tau)^{(1-\alpha)}\sin\left(\frac{\pi\alpha}{2}\right) + (\omega\tau)^{(2-2\alpha)}}$$

$$\chi''(\omega) = (\chi_T - \chi_S)\frac{(\omega\tau)^{(1-\alpha)}\cos\left(\frac{\pi\alpha}{2}\right)}{1 + 2(\omega\tau)^{(1-\alpha)}\sin\left(\frac{\pi\alpha}{2}\right) + (\omega\tau)^{(2-2\alpha)}}$$

where τ is the relaxation time, w is the angular frequency of the AC field and χ_T and χ_s are the isothermal and adiabatic susceptibilities, respectively. The α parameter (0 < α ≤ 1) describes the width of the distribution of relaxation times, with α = 0 corresponding to an infinitely sharp distribution.

The spin−lattice relaxation rate (τ^{-1}) from field dependent AC susceptibility data was fitted with the following model:[6-8]

$$\tau^{-1} = \tau^{-1}(Zeeman) + \tau^{-1}(Internal)$$

The first term signifies the direct mechanism between the two states split by the Zeeman energy, which is expected to vanish in zero field as a result of the Kramer's theorem. In principle, a pure and isolated S = 1/2 should not be able to relax in zero field. The second term takes into account a sort of internal field whose origin can be either intramolecular (i.e. hyperfine interactions) or intermolecular (i.e. due to dipolar or exchange interactions). So, the latter is responsible for the efficient relaxation in zero field and presents. Spin-spin and spin-nuclei interactions promote rapid relaxation at low fields. These interactions are suppressed by increasing the field due to the lower influence of the hyperfine and spin-spin coupling (vide infra). However, the larger the energy separation of the two levels, the higher the phonon density with an energy corresponding to this difference, leading to a more efficient spin−phonon direct mechanism of relaxation.

The relative microwave power (B_1) was calculated as:

$$B_1 = \sqrt{\frac{10^{-(0.1*A)}}{10^{-(0.1*Z)}}}$$

where A is the microwave power attenuation in dB and Z is the lowest microwave power attenuation in dB (7 dB).

Powder X-ray diffraction (PXRD) patterns obtained for complexes **1-Co** and **1-Cu** showed excellent agreement with the simulated patterns obtained from the single-crystal X-ray diffraction (SC-XRD) data (Figures S1 and S2), indicating crystalline phase purities in both co mplexes. Magnetic susceptibilities of solutions were measured using the Evans method.[9] NMR spectra of a solution of **1-Co** and **1-Cu** in acetonitrile-d_3 in a coaxial NMR tube were measured using NMR spectrometer (500 MHz) by Bruker.

1.5 EPR measurements

Continuous wave (CW) X-band EPR measurements at 6 K were carried out with a Bruker EMXPlus EPR spectrometer using Bruker Xenon software (ver. 1.2) at University of Nevada Reno. Remaining X-band and Q-band EPR experiments presented in this study were acquired at the Caltech EPR facility. X-band CW-EPR spectra were acquired on a Bruker (Billerica, MA) EMX spectrometer using Bruker Xenon software (ver. 1.2). Pulse EPR and electron nuclear double resonance (ENDOR) experiments were acquired using a Bruker ELEXSYS E580 pulsed EPR spectrometer using a Bruker MD4 pulsed ENDOR probe for X-band experiments or D2 pulsed ENDOR resonator for Q-band experiments. Temperature control was achieved using an Oxford Instruments CF-935 helium flow cryostat and a Mercury ITC temperature controller. Spectra were simulated using EasySpin (ver. 5.2.36)[10] with Matlab R2022b. Solid polycrystalline complexes **1-Co** and **1-Cu** were dissolved in acetonitrile-d_3: toluene-d_8 (1:1) (c.a. 1 mM), transferred to X-band or Q-band tubes, respectively, and rapidly cooled in liquid nitrogen to form a frozen glass. Pulse electron spin-echo-detected EPR field-swept spectra were recorded using the two-pulse "Hahn-echo" sequence (π/2−τ−π−echo). T_m measurements were performed using the same Hahn echo sequence (π/2−τ−π− echo) at fixed magnetic fields, with τ varied at regular intervals to measure the decay in echo intensity. T_1 measurements were performed using the inversion recovery pulse sequence (π−T−π/2−τ−π−echo), where T is a variable delay and τ is a fixed delay of 120 ns. Inversion recovery and echo decay experiments were fit using stretched-exponential eqs. 1 and 2, respectively.

$$I = I_0 + k_1 e^{-\left(\frac{t}{T_1}\right)^{b_1}} \quad (1)$$

$$I = I_0 + k_1 e^{-\left(\frac{2t}{T_m}\right)^{b_m}} \qquad (2)$$

Nutation experiments were performed by applying a tipping pulse (τp) to generate a superposition state, followed by followed by a Hahn-echo detection sequence (τ_p-T-π/2-τ-π-τ-echo), where τ_p was incremented in 2 ns steps with T = 600 ns, and π /2 and π pulse lengths of 8 ns and 16 ns, respectively. Acquisition parameters for Q-band Davies ENDOR: pulse sequence π-t_{RF}-π_{RF}-t_{RF}-π/2-τ–π–τ-echo; where t_{RF} = RF pulse delay, π_{RF} = RF pulse length. Specific values for these pulse and delay lengths are included in the captions of each ENDOR figure. The frequency of the RF pulse was randomly sampled to minimize nuclear spin saturation.

Q-band electron nuclear double resonance (ENDOR) spectroscopy. While the *g*-values and hyperfine A_z components of the Co and Cu hyperfine can be estimated from the previously discussed field swept CW- and EDFS EPR spectra, the smaller hyperfine tensor components A_x and A_y of the metal nuclei and all couplings from the 6 ^{14}N nuclei of the nitrogenous ligands are concealed within the inhomogenously broadened linewidth of these spectra. In order to detect and determine these smaller hyperfine couplings quantitatively, we turned to Q-band electron nuclear double resonance (ENDOR) spectroscopy. Field-dependent Davies ENDOR spectra were collected on frozen solutions (ca. 1 mM) of **1-Co** and **1-Cu** the same mixture of solvents (acetonitrile-d_3: toluene-d_8; 1:1) used for the X-band relaxation measurements, revealing signals from strongly coupled ($A > 2*\nu_I$) ^{14}N nuclei within the range from 5-30 MHz at all fields (Figures S16 and S17, left). At many fields, broad, highly anisotropic signals are also observed from ^{59}Co and $^{63/65}$Cu for **1-Co** and **1-Cu**, respectively, which are shown in wider ENDOR spectra. These spectra are well-simulated (in combination with the X-band CW-EPR and Q-band EDFS) by the

parameters contained within Tables S10 and S11. The EDFS spectra were pseudomodulated using the "fieldmod" function in Easyspin, which operates using the pseudo- field modulation algorithm reported by Hyde et al.[11] It essentially generates a filtered derivative, approximating the field modulation used for CW-EPR.

For **1-Co**, a single class of hyperfine coupling to ^{14}N is detected, with $A_{x,y,z}(^{14}N_1)$ = [21.6, 22.2, 31.9] MHz. For all nuclei with $I > 1/2$, the nuclear spin sublevels are additionally split by the nuclear quadrupole interaction (NQI), which arises from the interaction of the electric quadrupole of the nucleus with inhomogenous electric field gradients (efg) induced by charge density in p-orbitals, typically parameterized by the axially symmetric quadrupole coupling constant (e^2Qq_{zz}/h) and the EFG asymmetry parameter (η) which accounts for rhombicity in the electric field gradient. For this class of $^{14}N_1$ nucleus, the experimental data are well simulated using NQI parameters $e^2Qq/h(^{14}N_1)$ = -4.00 MHz and η = 0.15. The four equatorial nitrogen ligands exhibit far less orbital overlap with the dz^2 SOMO, and are predicted by DFT to be quite small (c.a. 2 MHz) and unsuitable to detection by the Davies ENDOR technique used here. Mims ENDOR or HYSCORE would likely be necessary to quantitatively measure these smaller couplings, and these weak couplings with little M-N covalency are not of critical importance to the relaxation behaviors examined in this comparison. The additional ENDOR signals from ^{59}Co (Figure S16, right) allow for more quantitative evaluation of the smaller A_x and A_y principal components, thus providing an estimate for the full metal hyperfine tensor, as well as the NQI for this I = 7/2 nucleus. Best-fit simulations of the combined datasets provide $A(^{59}Co)$ = [-46.0, -82.7, 230.8], $e^2Qq/h(^{59}Co)$ = -63.0 MHz and η = 0.11, in reasonable agreement with DFT predicted values (Tables S10 and S11), and consistent with predominant spin localization in a $d_z{}^2$ orbital, which would contribute a significant axial anisotropic hyperfine coupling component of the form [-2/5, -2/5, +4/5] $*\rho(d_z{}^2)$.

The low-frequency region of the ENDOR of **1-Cu** is considerably more complicated than that of **1-Co**, with signals from at least two general classes of ^{14}N nuclei evident in the range from 5-35 MHz, as well as *extremely* broad signals from $^{63/65}Cu$ which overlap considerably at orientations near g_x, g_y where the magnitude of this coupling is similar to that of the ^{14}N nuclei (Figure S17). The experimental data can be simulated with two distinct classes of ^{14}N with different hyperfine couplings tensors A($^{14}N_2$) = [46.3, 39.8, 35.8] MHz and A($^{14}N_3$) = [35.9, 33.8, 32.1] MHz, both of which are significantly larger than A($^{14}N_1$) measure for **1-Co.** The NQI parameters for these two classes of nitrogen are e^2Qq/h($^{14}N_2$) = -2.61 MHz and η($^{14}N_2$) = 0.27; e^2Qq/h($^{14}N_3$) = -2.67 MHz and η($^{14}N_3$) = 0.32. These NQI parameters differ considerably from those of the single class of ^{14}N detected for **1-Co**, with significantly lower magnitudes of e^2Qq/h (-2.16 and -2.67 MHz for **1-Cu** vs. -4.00 MHz for **1-Co**) and much higher EFG asymmetry (η = 0.27 and 0.32 for **1-Cu** vs. 0.15 for **1-Co**), and are broadly in line with those previously reported (e^2Qq/h = - 3.0 MHz, η = 0.2) for pyridine nitrogen ligands to a relevant Cu complex, determined using similar pulse EPR methods.[12] These values compare favorably with the DFT-predicted hyperfine and NQI parameters for the equatorial pyridine nitrogens ($^{14}N_2$, $^{14}N_2$’) of the N4 ligand and phenanthroline ($^{14}N_2$, $^{14}N_3$’), respectively, therefore we assign these classes as described in Tables S10 and S11.

In comparison to **1-Co**, signals from the metal hyperfine in **1-Cu** are rather poorly resolved due to extremely large ENDOR linewidths – reflective of the approximately doubled hyperfine anisotropy for this complex. Nonetheless, an estimate based on combined ENDOR and field-swept EPR spectral simulations provides an estimate of A(^{63}Cu) = [14.7, 50.4, -550] MHz, e^2Qq/h(^{63}Cu) = 63.0 MHz and η = 0.10, which is in reasonable agreement with predicted values from DFT calculations (Tables S10 and S11) and consistent with a $d_{x^2-y^2}$ SOMO which would be expected to contribute a significant axial anisotropic hyperfine coupling component of the form [+2/5, +2/5, -

4/5]*$\rho(d_{x^2-y^2})$. In addition to the principal hyperfine and NQI tensor components, the relative orientations of the coordinate frames of each of these tensors relative to the g-tensor frame (and by extension molecular frame) must be considered, particularly in the case of the nitrogen nuclei, though introduction of an additional 6 rotation vectors for each nucleus adds considerably to the parameter space one must consider in these relatively low-resolution spectra with overlapping lineshapes. As such, these additional simulation parameters likely do not represent a unique set of parameters which may satisfactorily reproduce the experimental data.

1.6 Electronic structure calculations

The electronic properties of **1-Co** and **1-Cu** were calculated in ORCA.[13] The TightSCF option was used in all calculations. The cation geometries were first extracted from the X-ray diffraction analysis and then the positions of the hydrogen atoms were optimized using the PBE density functional and the def2-SVP basis set.[14-15] The D3BJ model[16] was used to calculate the dispersion correction to the DFT energy. The Coulomb two-electron integrals (J) were calculated using the resolution of the identity (RI) approximation[17] together with the def2/J auxiliary basis set.[18]The hyperfine tensors for the metal ions were calculated in the EPRNMR module of ORCA using the hybrid B3PW91 density functional.[19-20]Co and Cu atoms were modeled using the CP(PPP) basis set,[21]whereas the EPR-II basis set was assigned to all non-metal atoms.[15] Vibrational frequencies of **1-Co** and **1-Cu** were calculated using the gas-phase optimized geometries and the □B97M-D3BJ/def2-SVP method.[22] The absence of imaginary modes testifies that the optimized geometries correspond to local minima. The first twenty vibrational modes calculated for **1-Co** and **1-Cu** are presented in Tables S6 and S7, respectively. Vibrational modes decomposition was done according to the vibrational mode automatic relevance determination approach.[23] All the frequencies are unscaled. Figures S39 and S40 present vibrational vectors of the lowest frequency modes of 38.49

and 34.05 cm^{-1} for **1-Co** and **1-Cu**, respectively. The RIJCOSX[24] algorithm was used to approximate both the Coulomb (RI-J) and Hartree-Fock exchange (COSX) integrals. The auxiliary basis set for RI-J was automatically constructed using the AutoAux algorithm.[25]The electronic absorption spectra and g-tensor were calculated as follows. First, the state average complete active space self-consistent field (SA-CASSCF) method together with the def2-TZVP basis set were used to calculate energies and wavefunctions of the ground and excited states. The RI-JK approximation designed for the orbital gradient and Hessian was used to speed up the CASSCF calculations. The active space included the d^7 configuration for the cobalt (II) complex and d^9 – for copper (II). The second order Douglas-Kroll-Hess (DKH2) Hamiltonian[14] was used to account for the scalar relativistic correction to the electronic energies. Second, the N-electron valence state second order perturbation theory (NEVPT2) was used to obtain accurate electronic absorption spectra. Third, the CASSCF states wavefunctions and spin-orbit mean-field (SOMF)[15] operator were used to construct and diagonalize the spin-orbit Hamiltonian matrix (with the NEVPT2 energies as the diagonal elements). Finally, the g-tensor was calculated for the ground spin-orbit Kramers' state. For **1-Cu**, SA-CASSCF included all the five doublet states arising from the d^9 configuration of copper (II). For **1-Co**, two separate calculations were performed. In the calculation of the NEVPT2 spectrum, state averaging included ten doublet and ten quartet states. In the calculation of the g-tensor, state averaging included only the ground doublet and first three quartet states which corresponded to the low-energy part of the spectrum.

In addition, we want to highlight the importance of using a proper level of theory for obtaining reasonable values of HFCC. Table S6 summarizes various methods used for the HFCC calculations. First, comparing the values from Table S5 and Table S6 (Case 1), we can conclude that there is only a negligible difference in the HFCC values associated with geometry change.

The use of the much larger aug-cc-pVTZ-J basis set does not have a strong effect on the magnitude of HFCC (Table S6, case 2). A larger change in the HFCC magnitude (but not in the anisotropy) is observed when using relativistic Hamiltonians with appropriate basis sets (Table S6, cases 3 and 4). As using the def2-TZVP basis set results in inferior HFCC values for Co/Cu atoms, it cannot be recommended for HFCC calculations in d-metals (Table S6, case 5). *w*B97M-D3bj functional in combination with the EPR-II{H, C, N}+CP(PPP){Co/Cu} basis set does reproduce A-tensor anisotropy in **1-Cu** complex, but it fails in the case of **1-Co** complex. In contrast, the PBE0 functional performs very similarly to B3PW91 (case 3 vs. case 4), and therefore, both functionals are recommended for the HFCC calculations.[26]

Calculation of the Zeeman splitting between components of the ground spin state in 1-Co and 1-Cu. We estimate the magnitude of the Zeeman splitting between components of the ground spin state in **1-Co** (**1-Cu**) by constructing and diagonalizing the Zeeman Hamiltonian under assumption of the complex oriented along the g-tensor principal axes. We calculate the splitting magnitude at different orientations of the magnetic field relative to the principal axes of the molecular g-tensor. The two components $|\psi_0\rangle$ and $|\psi_1\rangle$ of the ground spin state are degenerate in the absence of the external magnetic field. The splitting magnitude was estimated in

Simulation 1. Magnetic field is pointing along the *z* axis of the g-tensor. The wavefunctions $|\psi_0\rangle$ and $|\psi_1\rangle$ of the two components are taken to be $|\psi_0\rangle = |\alpha\rangle = |S = 1/2, M_S = 1/2\rangle$ and $|\psi_1\rangle = |\beta\rangle = |S = 1/2, M_S =- 1/2\rangle$.

Simulation 2. The field is not aligned with the *z* axis (arbitrary orientation; direction cosines are l_x = 0.616, l_y = -0.492, and l_z = 0.615). The wavefunctions of two components are those of Simulation 1.

Simulation 3. The field is not aligned with the *z* axis (different arbitrary orientation: direction cosines are l_x = -0.817, l_y = -0.531, and l_z = 0.225). The wavefunctions of two components are those of Simulation 1.

Simulation 4. The field is pointing along the *z* axis. The wavefunctions of two spin states are spin-mixed. This simulation pertains to **1-Co** only. The mixing coefficients are taken from the *ab initio* spin-orbit coupling calculations over the ground doublet and three lowest quartet states. The only one quartet state contributes to the spin-mixed wavefunctions of the ground spin-orbit state:

$|\psi_0\rangle = c_1^0|S = 3/2, M_S = 3/2\rangle + c_2^0|S = 1/2, M_S = 1/2\rangle$ and

$|\psi_1\rangle = c_1^1|S = 3/2, M_S = -3/2\rangle + c_2^1|S = 1/2, M_S = -1/2\rangle$,

where $c_1^0 =$ 0.006423 - 0.161481i, $c_2^0 =$ 0.974094 + 0.038195i, $c_1^1 =$ 0.161609i, and $c_2^1 =$ 0.974843.

Simulation 5. The field is not along the *z* axis (direction cosines are l_x = -0.817, l_y = -0.531, and l_z = 0.225). The ground state wavefunction is spin-mixed (**1-Co**).

The Zeeman Hamiltonian has the following form given the orientation of molecule along the principal axes of the g-tensor:

$$\hat{H} = \frac{\mu_B}{\hbar}\begin{pmatrix}|B|l_x & |B|l_y & |B|l_z\end{pmatrix}\begin{pmatrix} g_{xx} & 0 & 0 \\ 0 & g_{yy} & 0 \\ 0 & 0 & g_{zz}\end{pmatrix}\begin{pmatrix}\hat{S}_x \\ \hat{S}_y \\ \hat{S}_z\end{pmatrix} =$$

$$= \frac{\mu_B}{\hbar}|B|\left(g_{xx}l_x\hat{S}_x + g_{yy}l_y\hat{S}_y + g_{zz}l_z\hat{S}_z\right),$$

where μ_B is the Bohr magneton, $\hbar$ is the reduced Planck constant, g_{xx}, g_{yy}, and g_{zz} are principal g values, and $\hat{S}_{x,y,z}$ are the spin operators. The experimental g_{xx}, g_{yy}, and g_{zz} values and $|B|$ = 5000 Oe (0.5 T) are used in the simulations.

The Zeeman Hamiltonian in the bases of two spin states $|\psi_0\rangle$ and $|\psi_1\rangle$ takes the form:

$$H = \begin{pmatrix} \langle\psi_0\left|\hat{H}\right|\psi_0\rangle & \langle\psi_0\left|\hat{H}\right|\psi_1\rangle \\ \langle\psi_1\left|\hat{H}\right|\psi_0\rangle & \langle\psi_1\left|\hat{H}\right|\psi_1\rangle \end{pmatrix}.$$

Diagonalization of H yields energies E_2 and E_1 of the two Zeeman states, with the energy difference between them defining the magnitude of the splitting.

The results of Simulations 1-5 are summarized in Table S24.

2. Data

2.1 Characterization of 1-Co and 1-Cu

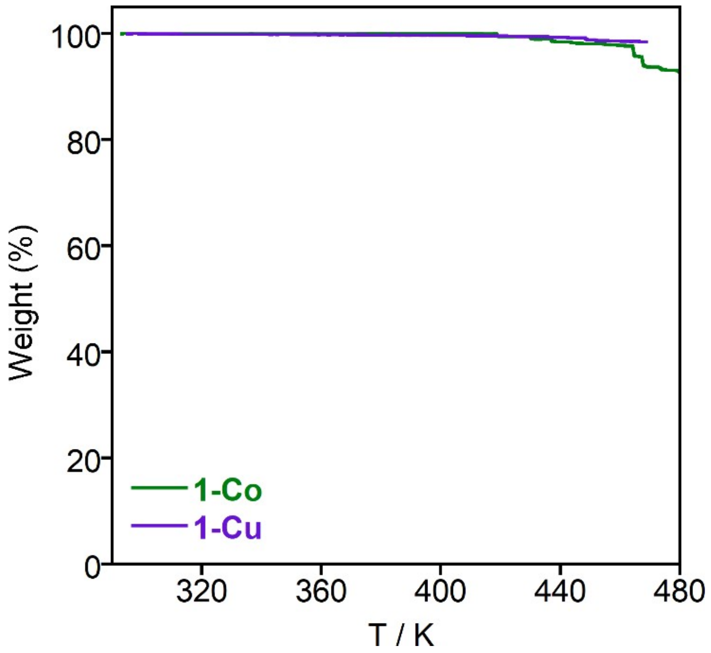

Figure S1 TGA plots for **1-Co** and **1-Cu**.

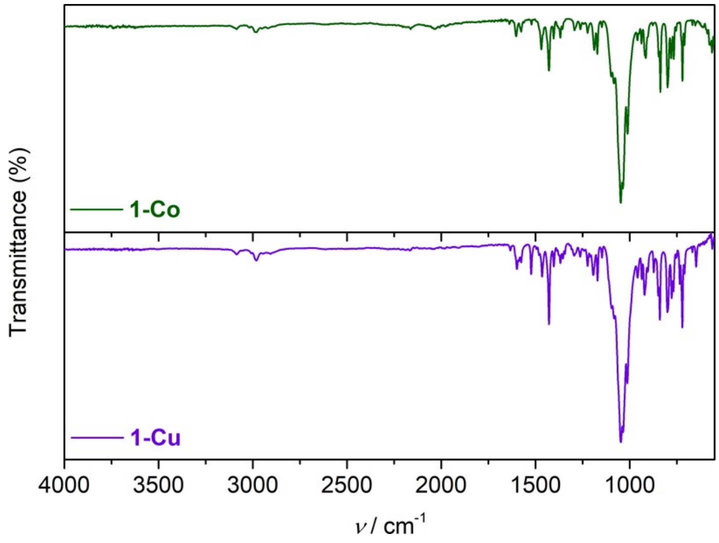

Figure S2 IR spectra of complexes **1-Co** and **1-Cu** at 300 K.

Electrospray Ionization Time-of-Flight mass spectrometry (ESI-TOF MS) measurements were carried out in acetonitrile at 300 K. The presence of the $[Co(^{t}Bu\text{-}N4)(Phen)]^{2+}$ for complex **1-Co** and $[Cu(^{t}Bu\text{-}N4)(Phen)]^{2+}$ for complex **1-Cu** in solution was supported by ESI-TOF MS measurements in acetonitrile: toluene (1:1) at 300 K (Figures S3 - S6). The ESI-mass spectra of **1-Co** and **1-Cu** show a prominent ion peak at a *m*/*z* of 295.6317 and *m*/*z* of 297.6301 respectively (Figures S3 and S4), whose mass and isotope distribution pattern corresponds to $[Co(^{t}Bu\text{-}N4)(Phen)]^{2+}$ (calculated *m*/*z* 295.6322) and $[Cu(^{t}Bu\text{-}N4)(Phen)]^{2+}$ (calculated *m*/*z* 297.6300) (Figures S5 and S6).

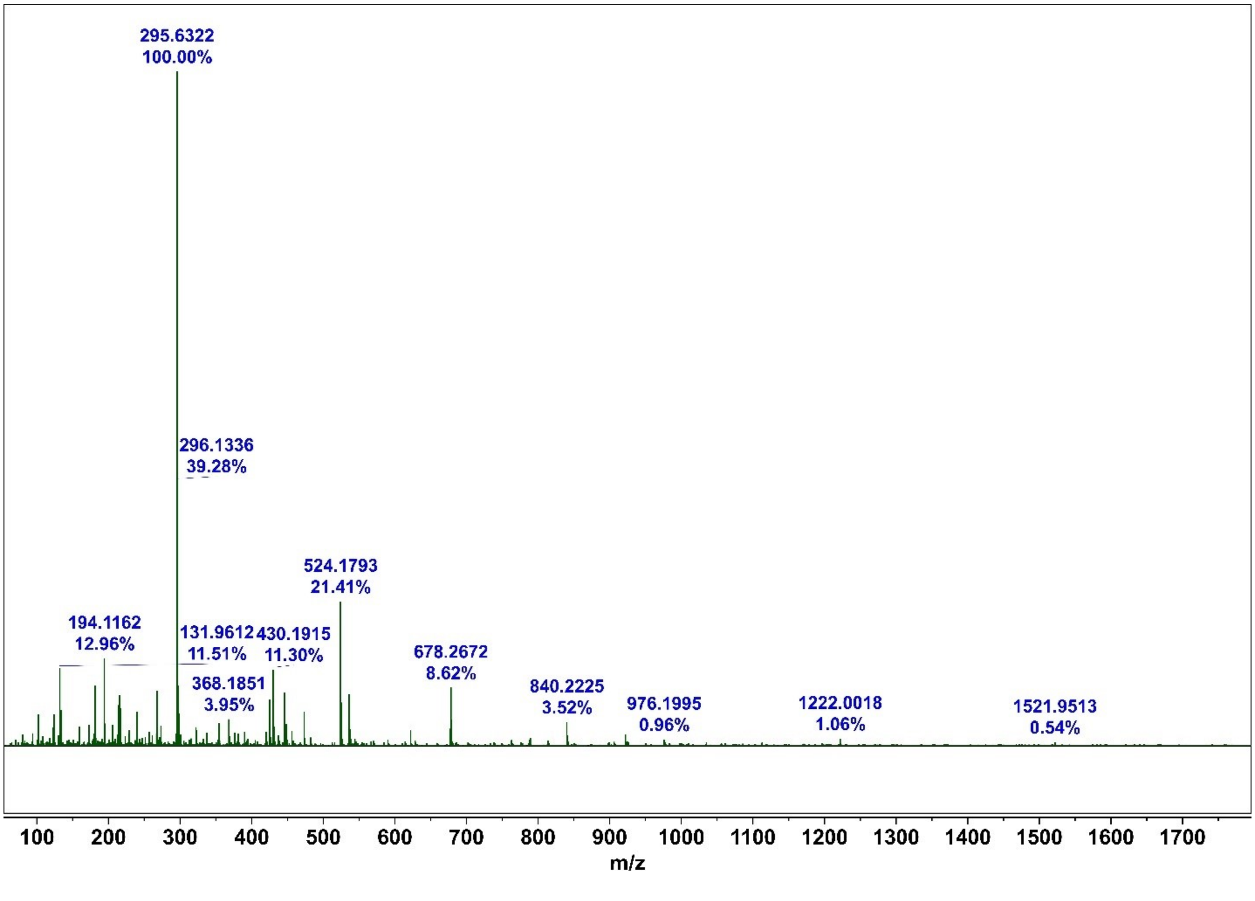

Figure S3 ESI mass spectrum of **1-Co** in acetonitrile: toluene (1:1) at 300 K.

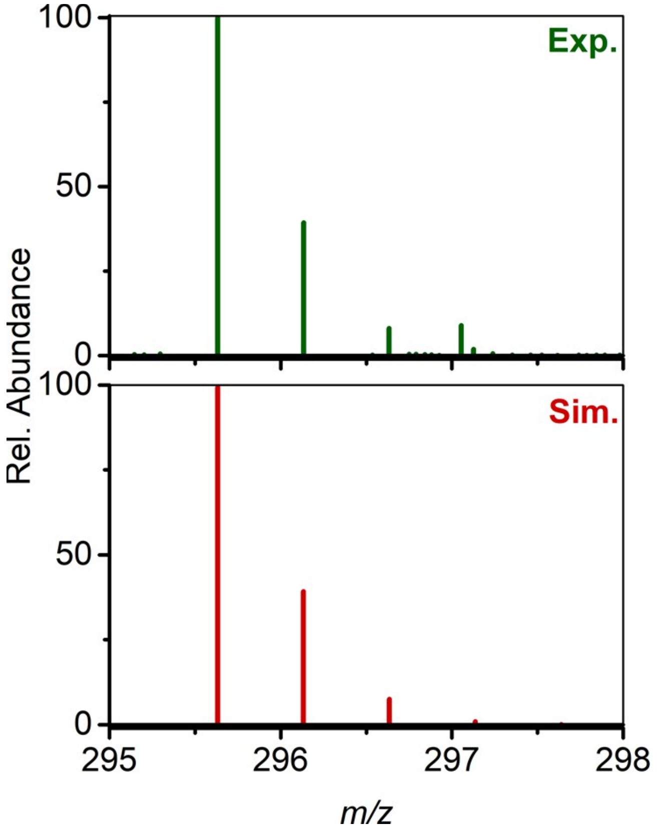

Figure S4 Isotope distribution for [Co(^{t}Bu-N4)(Phen)]$^{2+}$ (experimental, top; and calculated, bottom) in **1-Co** in acetonitrile: toluene (1:1) at 300 K.

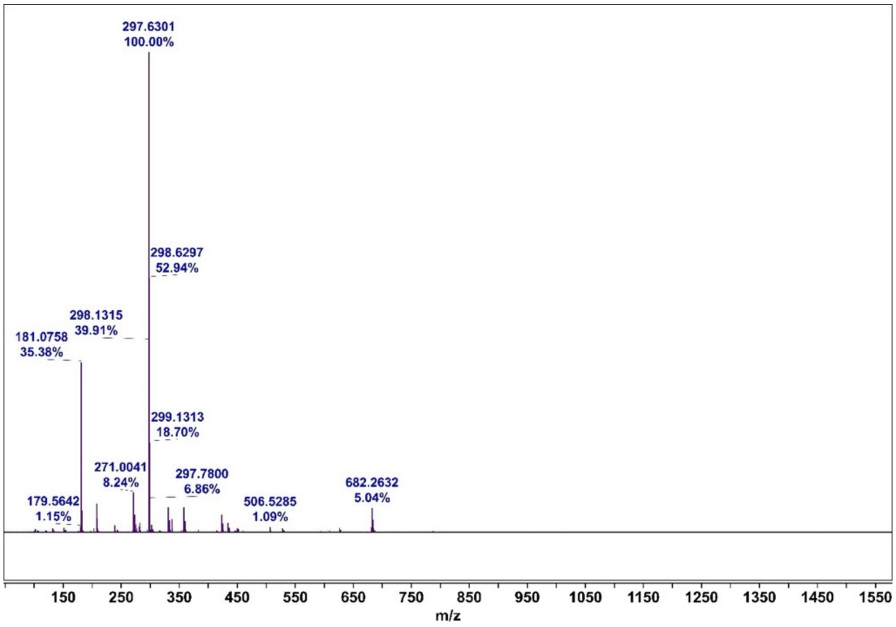

Figure S5 ESI mass spectrum of **1-Cu** in acetonitrile: toluene (1:1) at 300 K.

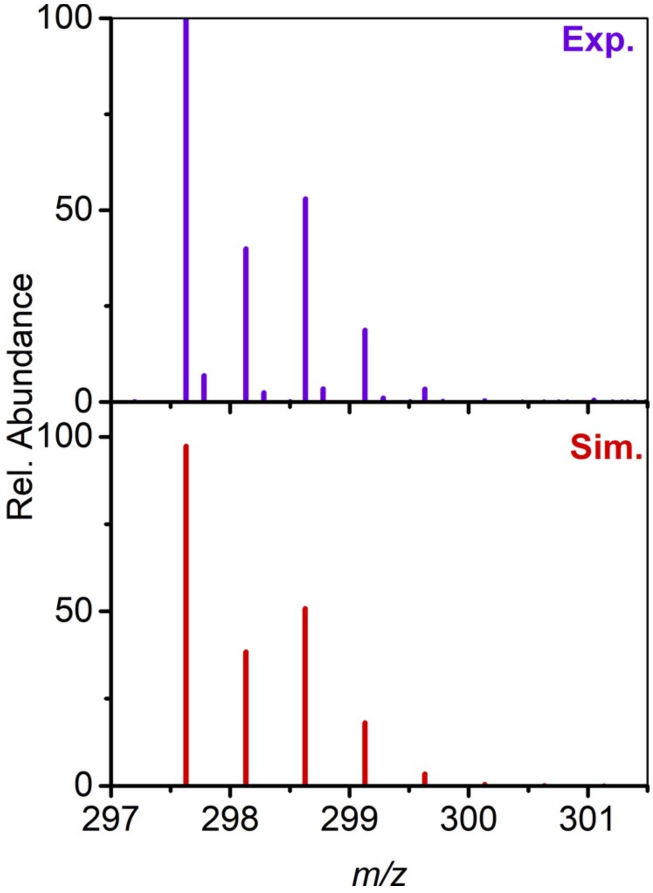

Figure S6 Isotope distribution for [Cu(^{t}Bu-N4)(Phen)]$^{2+}$ (experimental, top; and calculated, bottom) in **1-Cu** in acetonitrile: toluene (1:1) at 300 K.

2.2 X-ray crystallography

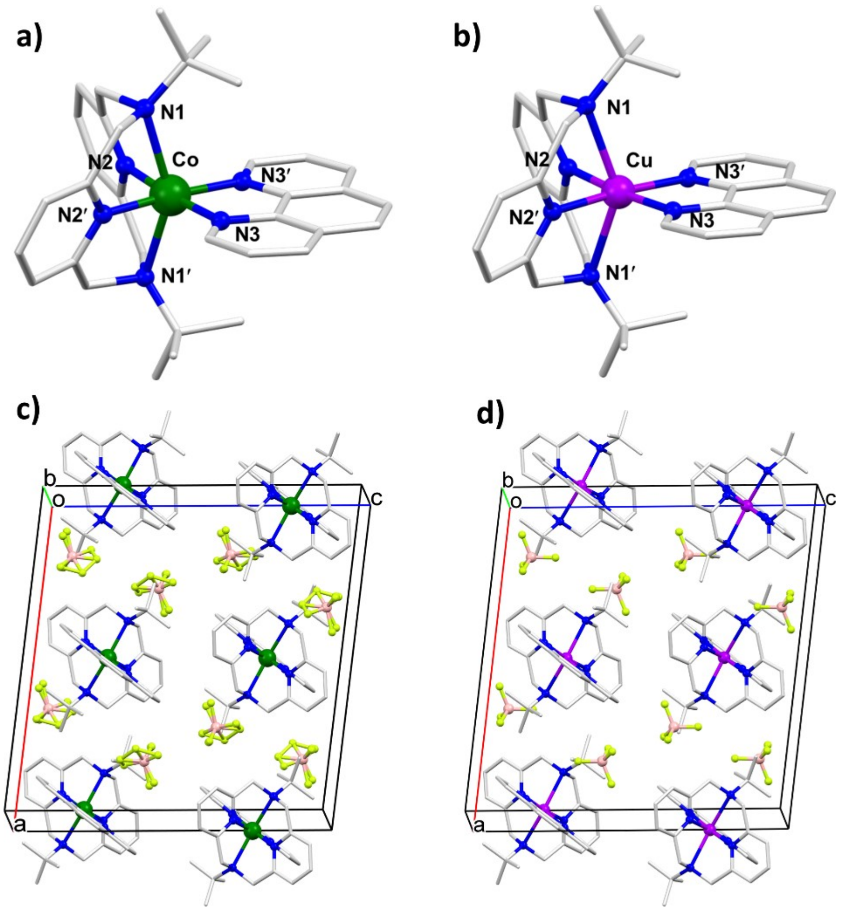

Figure S7 Top: Single crystal X-ray structure of complex cation in **1-Co** (a) and **1-Cu** (b) at 100 K. Hydrogen atoms and counter anions are omitted for clarity (Co, green; Cu, purple; C, gray; N, blue). Bottom: Unit cell packing diagram of **1-Co** (c) and **1-Cu** (d). Hydrogen atoms are omitted for clarity. (Co, green; Cu, purple; C, gray; N, blue; B, light pink; F, light-green).

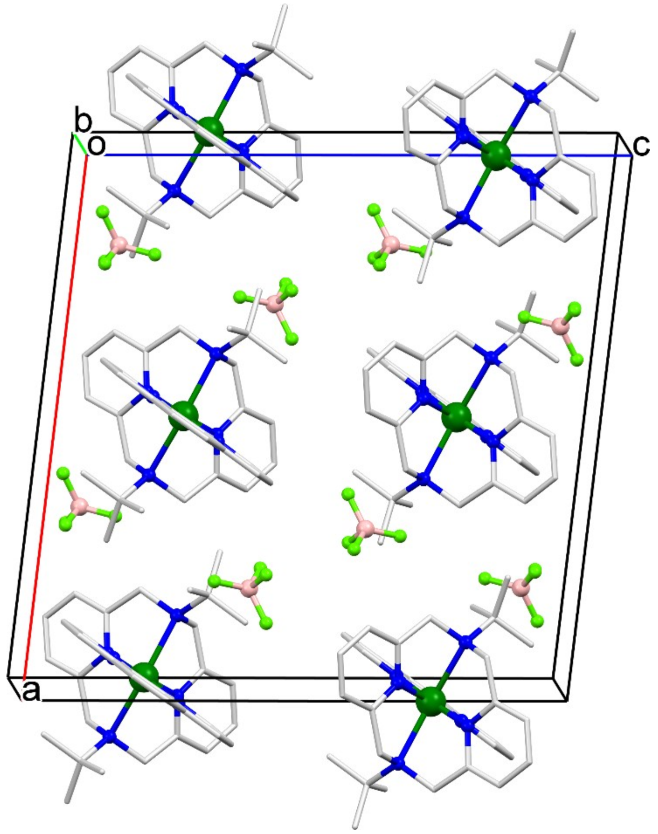

Figure S8 Unit cell packing diagram of **1-Co** at 296 K. Hydrogen atoms are omitted for clarity. (Co: green, C: grey, N: blue; B: light-pink; F: light-green.

Table S1 X-ray crystallography data for complexes **1-Co** and **1-Cu**.

	**1-Co**		**1-Cu**
CCDC no	2320669	2320670	2320671
temp (K)	296	100	100
empirical formula	$C_{34}H_{40}B_2CoF_8N_6$	$C_{34}H_{40}B_2CoF_8N_6$	$C_{34}H_{40}B_2CuF_8N_6$
formula wt	765.27	765.27	769.88
cryst syst	Monoclinic	Monoclinic	Monoclinic
space group	$C2_1/c$	$C2_1/c$	$C2_1/c$
a (Å)	18.1532(10)	18.1452(13)	18.209(3)
b (Å)	10.6741(6)	10.5740(8)	10.6641(15)
c (Å)	17.9568(10)	17.5799(13)	17.517(3)
a (deg)	90	90	90
b (deg)	96.5250(10)	96.7290(10)	96.698(2)
g (deg)	90	90	90
V, Å^3	3456.9(3)	3349.8(4)	3378.4(8)
Z	8	8	8
d_{calcd} (g cm^{-3})	1.470	1.517	1.514
m (mm^{-1})	0.575	0.593	0.727
F(000)	1580	1580	1588
q_{max} (deg)	30.579	30.603	27.494
completeness (%)	99.9	99.9	100
no. of. rflns collected	5293	5142	3186
no. of. Indep rflns	3533	4047	3884
goodness of fit on F^2	1.069	1.025	1.032
final R indices (I>2s(I))	R1 = 0.0627	R1 = 0.0421	R1 = 0.0570
	wR2 = 0.1730	wR2 = 0.0967	wR2 = 0.1438
final R indices (all data)	R1 = 0.0965	R1 = 0.0604	R1 = 0.0720
	wR2 = 0.1972	wR2 = 0.1060	wR2 = 0.1562

R1 = $\sum||Fo| - |Fc||/\sum|Fo|$ and wR2= $|\sum w(|Fo|^2 - |Fc|^2)|/\sum|w(Fo)^2|^{1/2}$

Table S2 Selected bond distances (Å) and bond angles in **1-Co** at 100 and 296 K.

	Exp. at 296 K	Exp. at 100 K	Calculated
Co(1)-N(1)	2.368(2)	2.3581(14)	2.484
Co(1)-N(2)	1.918(2)	1.9208(14)	1.949
Co(1)-N(3)	1.975(2)	1.9726(14)	2.005
N(1)-Co(1)-N(1)	149.75(11)	149.79(7)	149.79
N(1)-Co(1)-N(2)	77.59(8)	77.43(5)	77.94
N(1)-Co(1)-N(2)	81.11(8)	81.30(5)	80.40
N(1)-Co(1)-N(3)	105.12(8)	105.74(5)	102.95
N(1)-Co(1)-N(3)	97.52(8)	96.88(5)	99.62
N(2)-Co(1)-N(2)	89.83(13)	89.91(8)	87.85
N(2)-Co(1)-N(3)	93.80(9)	93.71(6)	94.63
N(2)-Co(1)-N(3)	175.76(9)	175.51(6)	177.47
N(3)-Co(1)-N(3)	82.66(13)	82.81(8)	82.89

Table S3 Selected bond distances (Å) and bond angles in **1-Cu**.

	Exp. at 100 K.	Calculated
Cu(1)-N(1)	2.439(2)	2.5505
Cu(1)-N(2)	1.994(3)	2.0251
Cu(1)-N(3)	2.038(2)	2.0801
N(1)-Cu(1)-N(1)	144.78(12)	144.911
N(1)-Cu(1)-N(2)	75.53(9)	75.972
N(1)-Cu(1)-N(2)	79.32(9)	78.611
N(1)-Cu(1)-N(3)	108.45(9)	105.249
N(1)-Cu(1)-N(3)	98.10(9)	101.264
N(2)-Cu(1)-N(2)	88.01(14)	86.3007
N(2)-Cu(1)-N(3)	95.23(10)	96.3458
N(2)-Cu(1)-N(3)	175.26(10)	177.253
N(3)-Cu(1)-N(3)	81.75(14)	81.0181

Continuous Shape Measures (CShM) Analysis

Continuous Shape Measures (CShM) analysis was carried out to determine the geometry around the copper center using Shape 2.1 software.[27-28] Based on the values obtained, the idealized polyhedron was matched with the actual coordination spheres. The smallest value is symbolic of the proximity of the actual coordination sphere and the idealized polyhedron.

Table S4 CShM analysis data for complexes **1-Co** and **1-Cu**.

Complex	Temp.	Structure				
		HP - 6	PPY - 6	**OC - 6**	TPR - 6	JPPY - 6
**1-Co**	296 K	31.638	23.536	**3.219**	10.699	27.090
	100 K	31.340	23.513	**3.191**	10.466	26.805
**1-Cu**	100 K	30.985	22.626	**3.918**	9.925	25.961

HP – 6: Hexagon (D6h), PPY – 6 = Pentagonal pyramid, **OC – 6: Octahedron (Oh)**, TPR – 6: Trigonal prism (D3h), JPPY – 6 = Johnson pentagonal pyramid J2 (C5v);

Octahedral Distortion Parameters

Octahedral distortion parameters (∑, Θ, ζ) and average metal-ligand bond distance (⟨M-N⟩) were calculated using OctaDist tool.[29] ∑ is the sum of the deviation from 90° of the 12 *cis*-angles of the MN_6 octahedron; Θ is the sum of the deviation from 60° of the 24 trigonal angles of the projection of the MN_6 octahedron onto the trigonal faces; ζ is the distance distortion parameter, which is the sum of deviation from individual M-N bond distances to the mean metal-ligand bond distance.

Table S5 Octahedral distortion parameters for complexes **1-Co** and **1-Cu**.

	**1-Co**		**1-Cu**
T/ K	296	100	100
Σ_M/ °	103.03	102.46	124.07
Θ_M / °	337.00	340.05	390.17
ζ / Å	1.126	1.097	1.128
⟨M-N⟩ / Å	2.087	2.0838	2.157

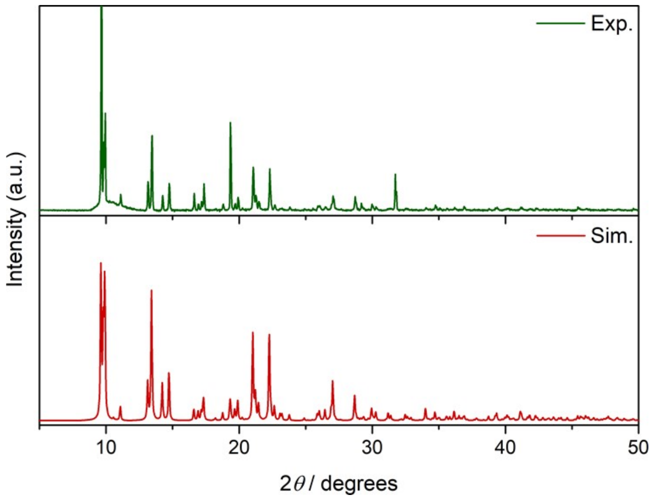

Figure S9 Comparison of the 300 K experimental PXRD pattern and simulation (296 K) for **1-Co**.

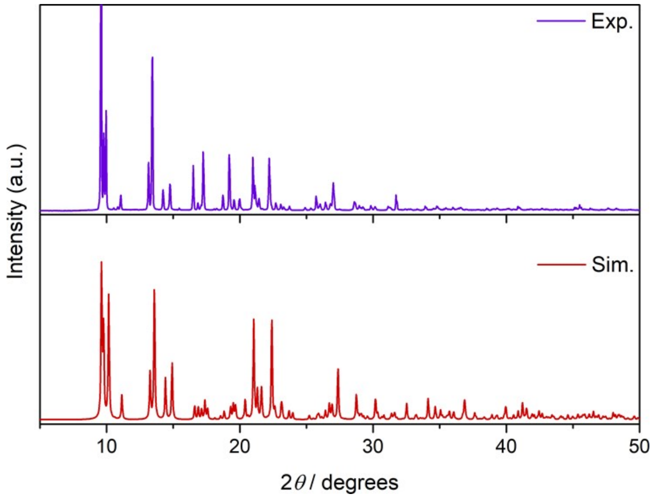

Figure S10 Comparison of the 300 K experimental PXRD pattern and the simulation (100 K) for **1-Cu**.

2.3 Magnetic measurements

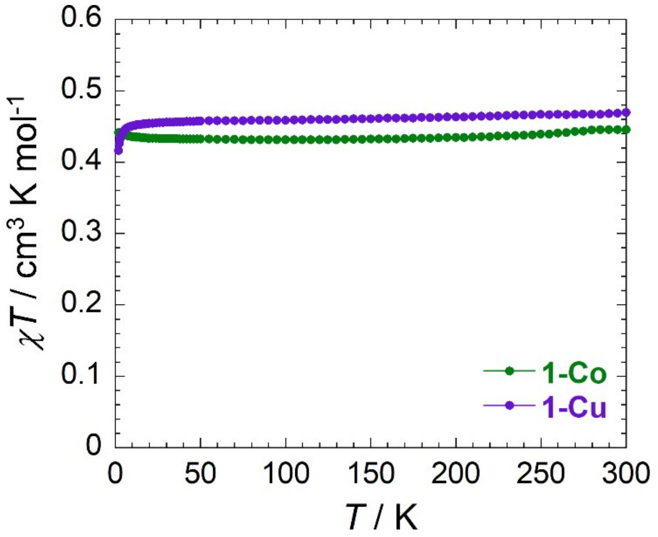

Figure S11 Temperature dependence of χT for **1-Co** and **1-Cu** at 1000 Oe.

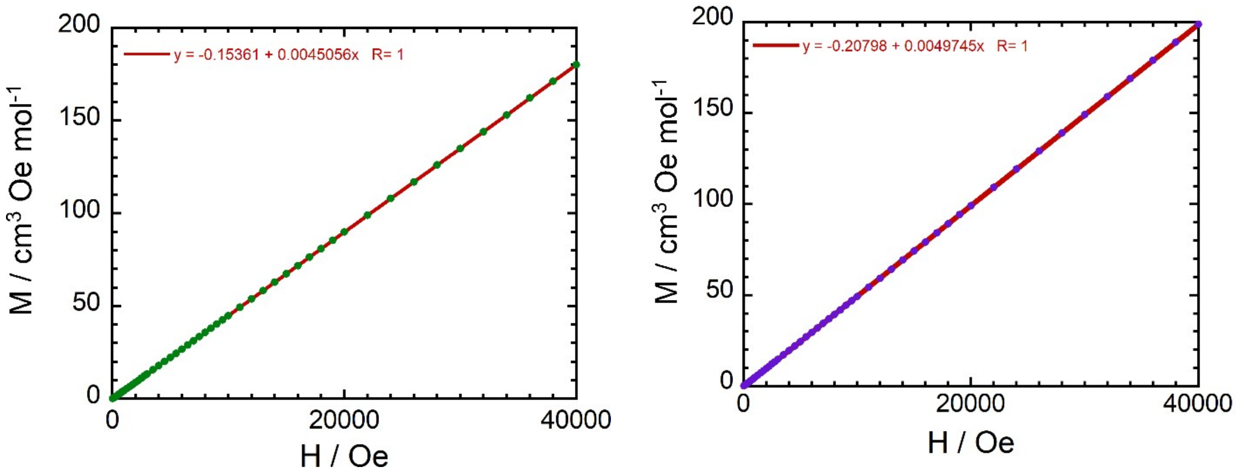

Figure S12 Field dependence of the magnetization as *M vs H* plots for **1-Co** (left) and **1-Cu** (right) at 100 K. The solid lines are the best fit.

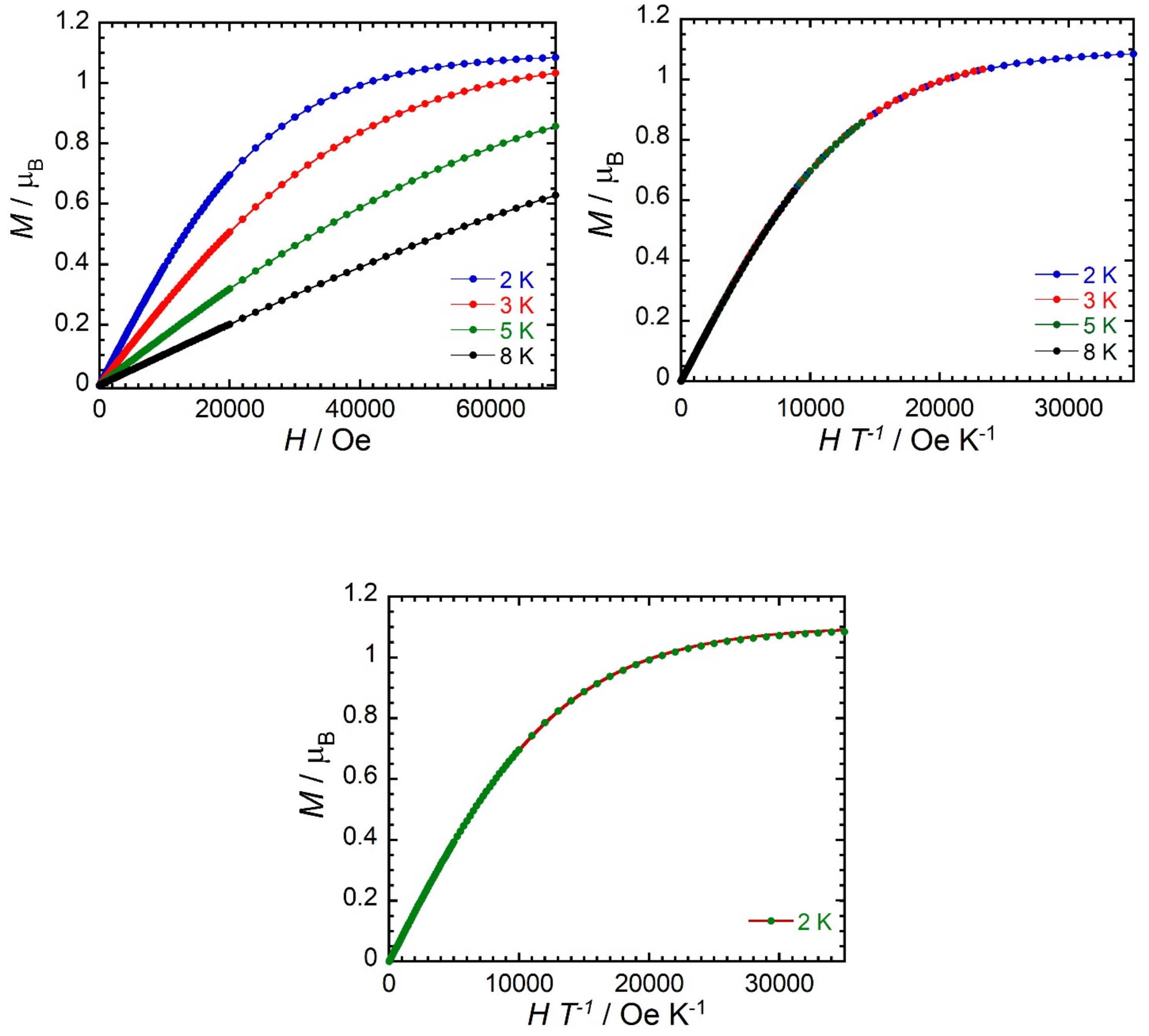

Figure S13 Top: Field dependence of the magnetization as *M vs H* (left) and *M vs H/T* (right) plots for **1-Co** at 2, 3, 5, and 8 K. The solid lines are a guide for the eyes. Bottom: Brillouin function fit (red line) to the *M vs H/T* at 2 K.

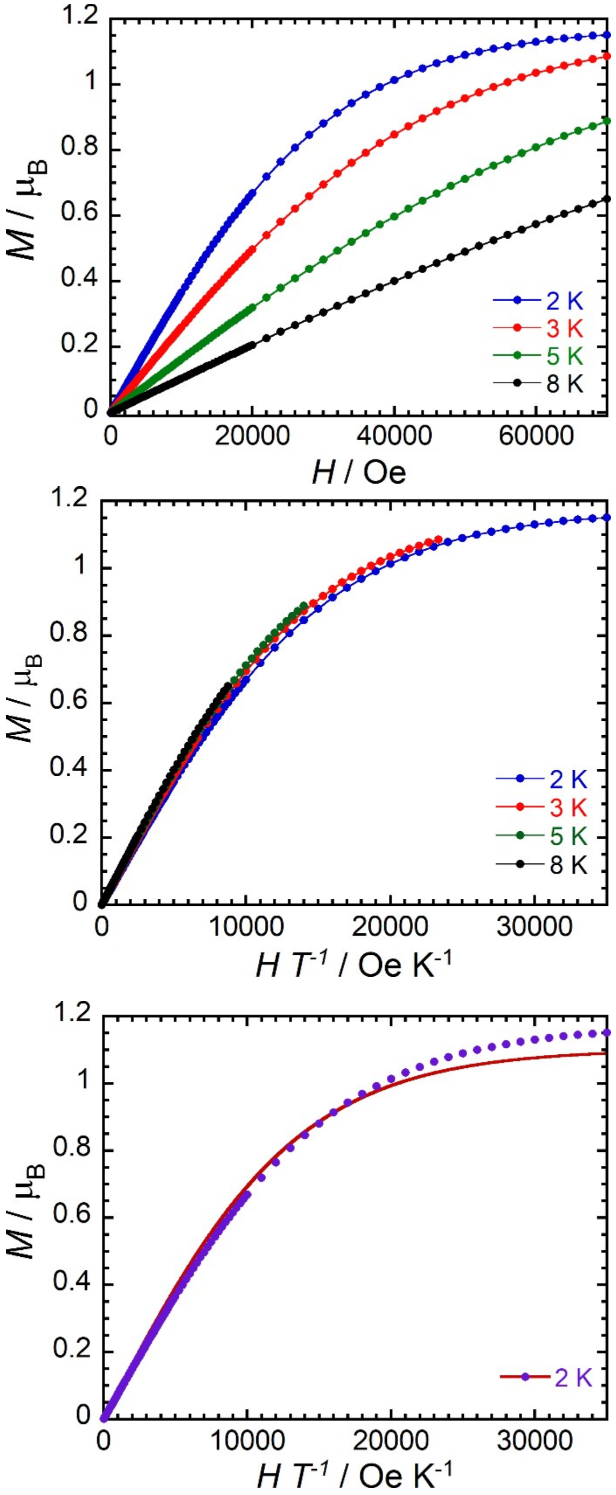

Figure S14 Top: Field dependence of the magnetization as *M vs H* (left) and *M vs H/T* (right) plots for **1-Cu** at at 2, 3, 5, and 8 K. The solid lines are a guide for the eyes. Bottom: Brillouin function fit (red line) to the *M vs H/T* at 2 K.

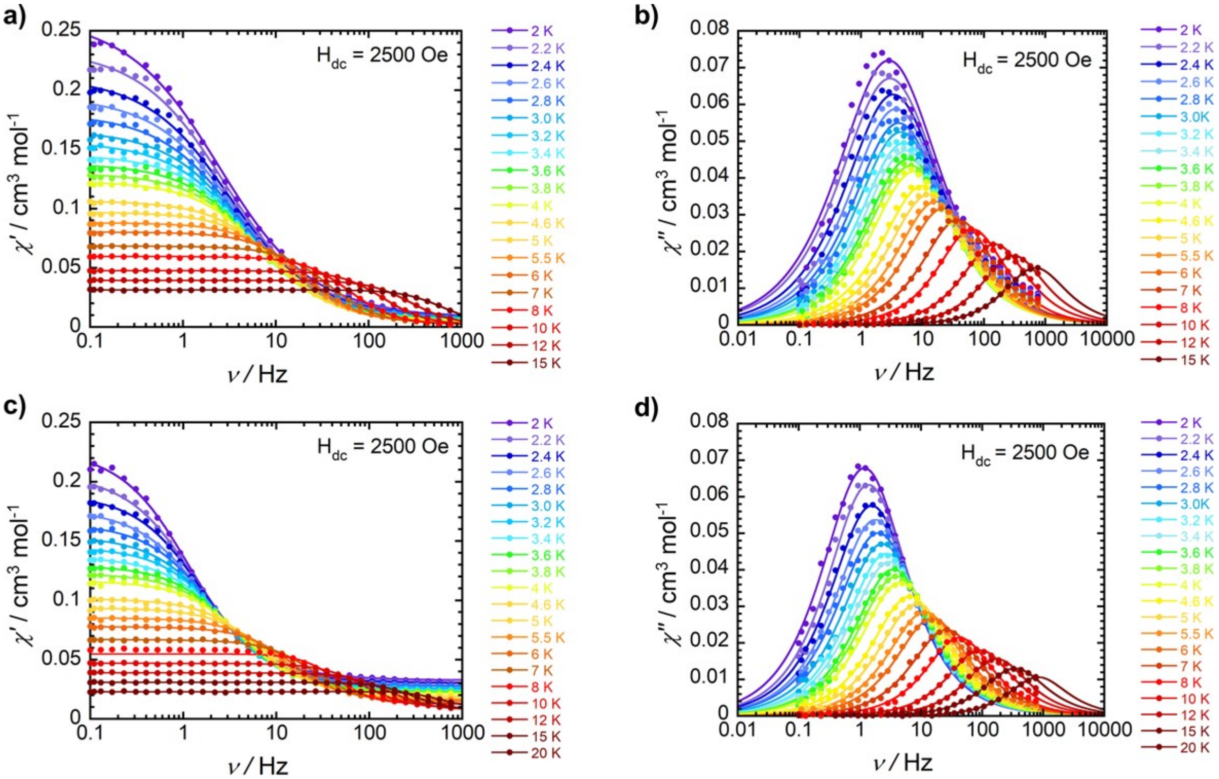

Figure S15 Frequency dependence of the real (χ', left) and imaginary (χ'', right) components of the AC susceptibility at different AC frequencies from 1 - 1000 Hz under 2500 Oe external DC field, with a 3 Oe AC field for **1-Co** (a and b) **1-Cu** (c and d) at different temperatures. Full lines correspond to fit with generalized Debye model.

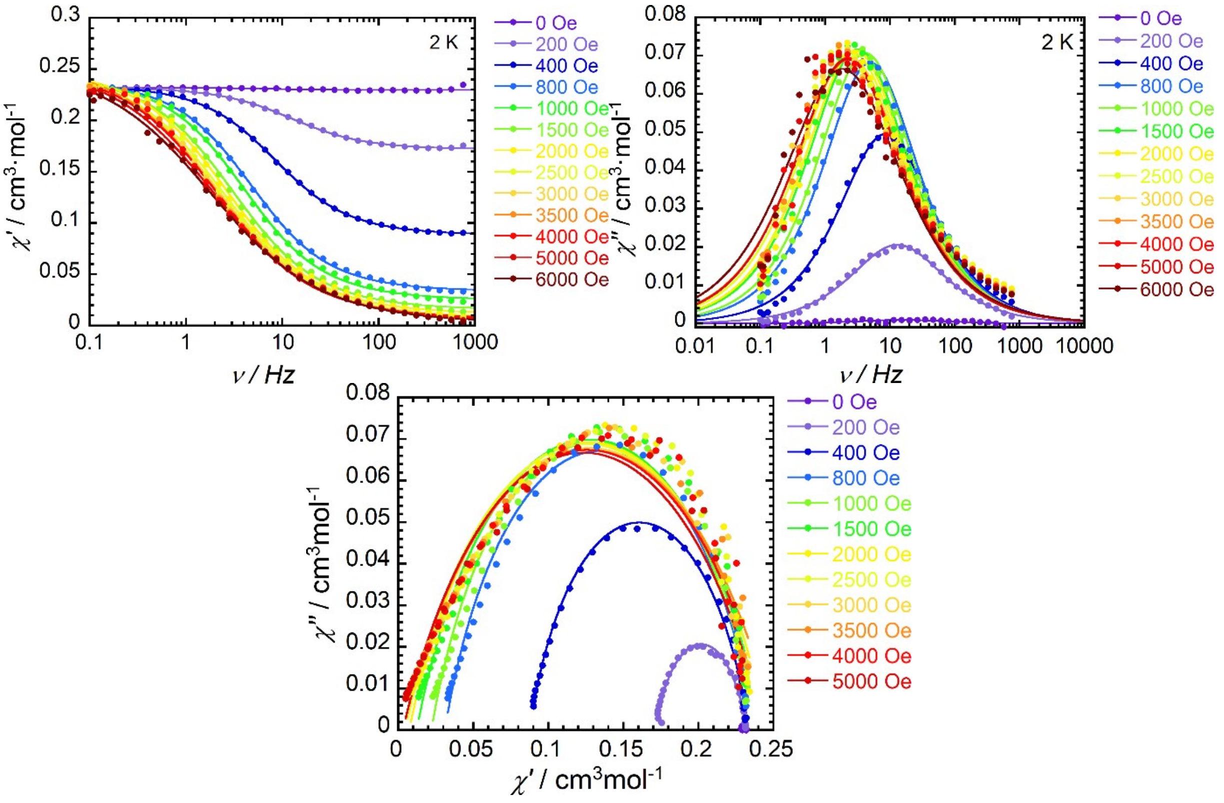

Figure S22 Top: Frequency dependence of the real (χ', left) and imaginary (χ'', right) components of the ac susceptibility at different ac frequencies from 1 - 1000 Hz different external dc field from 0 – 6000 Oe, respectively with a 3 Oe ac field for **1-Co** at 2 K. Full lines correspond to fit with Generalized Debye model. Bottom: The Cole-Cole plot with Generalized Debye fit.

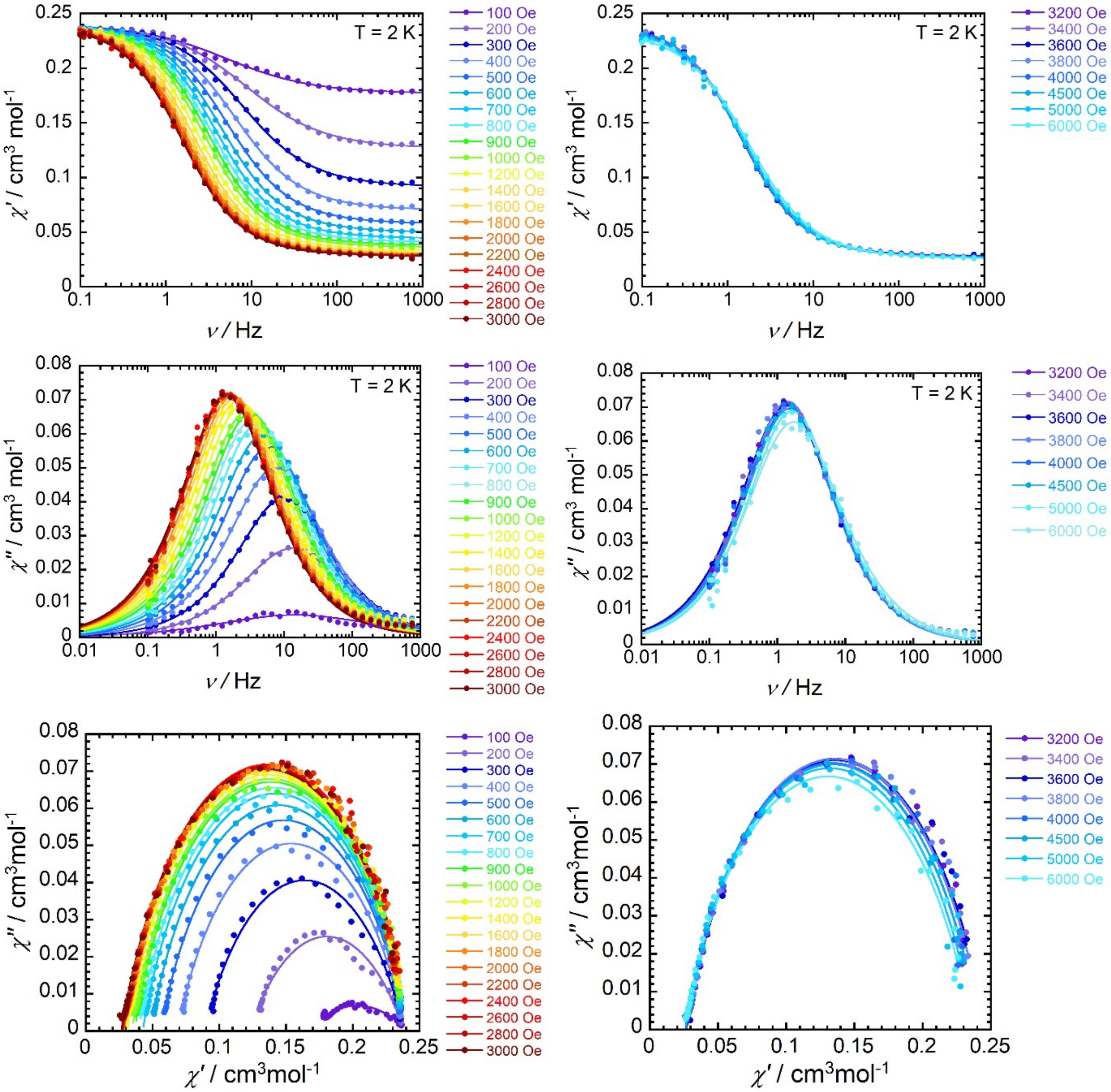

Figure S17 Frequency dependence of the real (χ', top) and imaginary (χ'', middle) components of the ac susceptibility at different ac frequencies from 1 - 1000 Hz different external dc field from 100 – 6000 Oe, respectively with a 3 Oe ac field for **1-Cu** at 2 K. Full lines correspond to fit with Generalized Debye model. Bottom: The Cole-Cole plot with Generalized Debye fit.

Table S6 Fitting parameters used in the generalized Debye model for variable temperature AC susceptibility data collect of **1-Co** at 2500 Oe.

T (K)	α	$\chi_T - \chi_S$	τ (s)
2.0000	0.30598	0.23736	0.0562
2.2000	0.30198	0.21965	0.053631
2.4000	0.28178	0.19823	0.047357
2.6000	0.26822	0.18149	0.042918
2.8000	0.26018	0.16731	0.038836
3.0000	0.25179	0.15762	0.035626
3.2000	0.24053	0.14581	0.031671
3.4000	0.23665	0.13876	0.029089
3.6000	0.22809	0.13046	0.026481
3.8000	0.23315	0.1261	0.024363
4.0000	0.21561	0.11798	0.021701
4.6000	0.19192	0.10292	0.015655
5.0000	0.17678	0.094051	0.012558
5.5000	0.1562	0.086118	0.0095488
6.0000	0.14344	0.079745	0.007275
7.0000	0.11134	0.06827	0.0041857
8.0000	0.085439	0.059705	0.0024891
10.000	1.0022	0.010731	2.7528e-159
12.000	0.040104	0.040294	0.00052182
15.000	0.033492	0.032798	0.00023046

Table S7 Fitting parameters used in the generalized Debye model for variable field AC susceptibility data collect of **1-Co** at 2 K.

Field (Oe)	α	$\chi_T - \chi_S$	τ (s)
200.00	0.24569	0.06109	0.01215
400.00	0.24577	0.14729	0.018398
800.00	0.24871	0.20357	0.031858
1000.0	0.25545	0.21353	0.038062
1500.0	0.28289	0.22689	0.049823
2000.0	0.28794	0.22816	0.054424
2500.0	0.30872	0.23536	0.06231
3000.0	0.32571	0.23792	0.067463
3500.0	0.32425	0.23519	0.068662
4000.0	0.31777	0.23381	0.070379
5000.0	0.31456	0.23113	0.072159

Table S8 Fitting parameters used in the generalized Debye model for variable temperature AC susceptibility data collect of **1-Cu** at 2500 Oe.

T (K)	α	χ_T - χ_S	τ (s)
2.00	0.20504	0.20504	0.13402
2.20	0.18655	0.18655	0.11539
2.40	0.1932	0.1932	0.10174
2.60	0.1888	0.1888	0.088265
2.80	0.19627	0.19627	0.079513
3.00	0.18486	0.18486	0.067959
3.20	0.17366	0.17366	0.058882
3.40	0.17894	0.17894	0.051834
3.60	0.16682	0.16682	0.044765
3.80	0.17345	0.17345	0.040002
4.00	0.16035	0.16035	0.034792
4.60	0.1526	0.1526	0.024234
5.00	0.14324	0.14324	0.018748
5.50	0.13327	0.13327	0.013743
6.00	0.11537	0.11537	0.010391
7.00	0.10445	0.10445	0.0060768
8.00	0.077261	0.077261	0.0037337
10.0	0.056701	0.056701	0.0017105
12.0	0.036925	0.036925	0.00091498
15.0	0.037101	0.037101	0.0004584
20.0	0.00035446	0.00035446	0.00019098

Table S9 Fitting parameters used in the generalized Debye model for variable field AC susceptibility data collect of **1-Cu** at 2 K.

Field (Oe)	α	χ_T - χ_S	τ (s)
100.00	0.47814	0.030768	0.010727
200.00	0.25954	0.079883	0.012127
300.00	0.22595	0.11805	0.015848
400.00	0.21858	0.14204	0.020953
500.00	0.21196	0.15727	0.026747
600.00	0.20983	0.16676	0.032552
700.00	0.2036	0.17436	0.038095
800.00	0.20702	0.17924	0.04456
900.00	0.20423	0.1828	0.049141
1000.0	0.20948	0.1862	0.05526
1200.0	0.20289	0.18945	0.064409
1400.0	0.19907	0.1914	0.074559
1600.0	0.20089	0.19441	0.08244
1800.0	0.19547	0.19414	0.090538
2000.0	0.20189	0.1978	0.098527
2200.0	0.19091	0.19475	0.10165
2400.0	0.19509	0.19668	0.10679
2600.0	0.19361	0.19461	0.10781
2800.0	0.19209	0.19407	0.10592
3000.0	0.20043	0.1953	0.10983
3200.0	0.20249	0.19717	0.11221
3400.0	0.20658	0.19764	0.11365
3600.0	0.21778	0.20026	0.11603
3800.0	0.19519	0.19574	0.11198
4000.0	0.20565	0.19513	0.10898
4500.0	0.20759	0.19398	0.10514
5000.0	0.19678	0.18857	0.096673

2.4 Electronic absorption spectroscopy

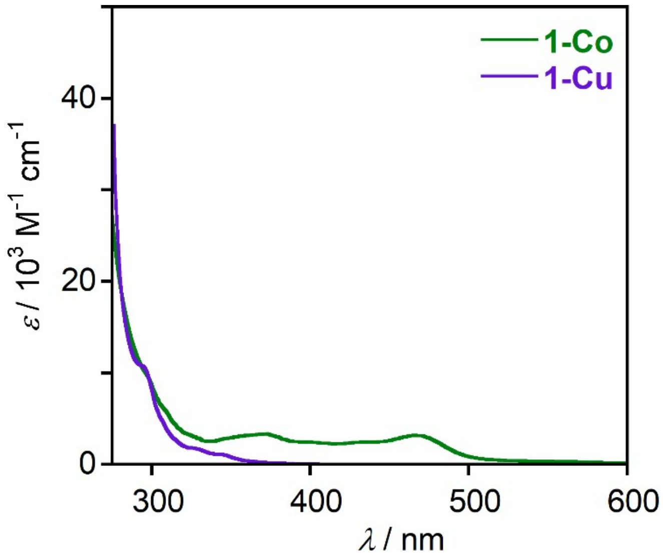

Figure S18 UV-vis spectra of **1-Co** and **1-Cu** in acetonitrile: toluene (1:1) at 300 K.

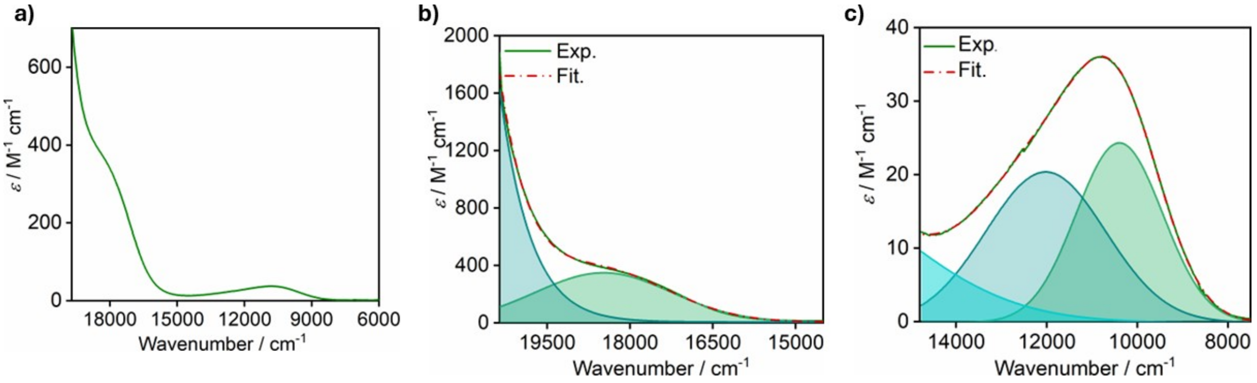

Figure S19 UV-vis-NIR spectrum of **1-Co** in 1 mM acetonitrile: toluene (1:1) solution at 300 K (a) along with the deconvoluted spectra (b and c).

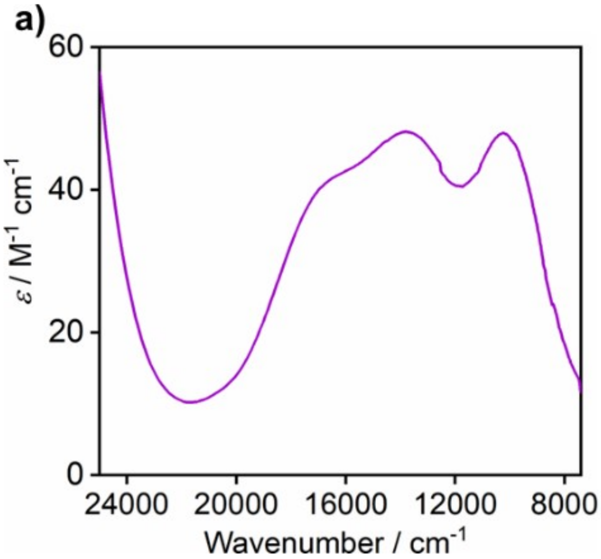

Figure S20 UV-vis-NIR spectrum of **1-Cu** in 1 mM acetonitrile: toluene (1:1) solution at 300 K (a) along with deconvoluted spectra (b).

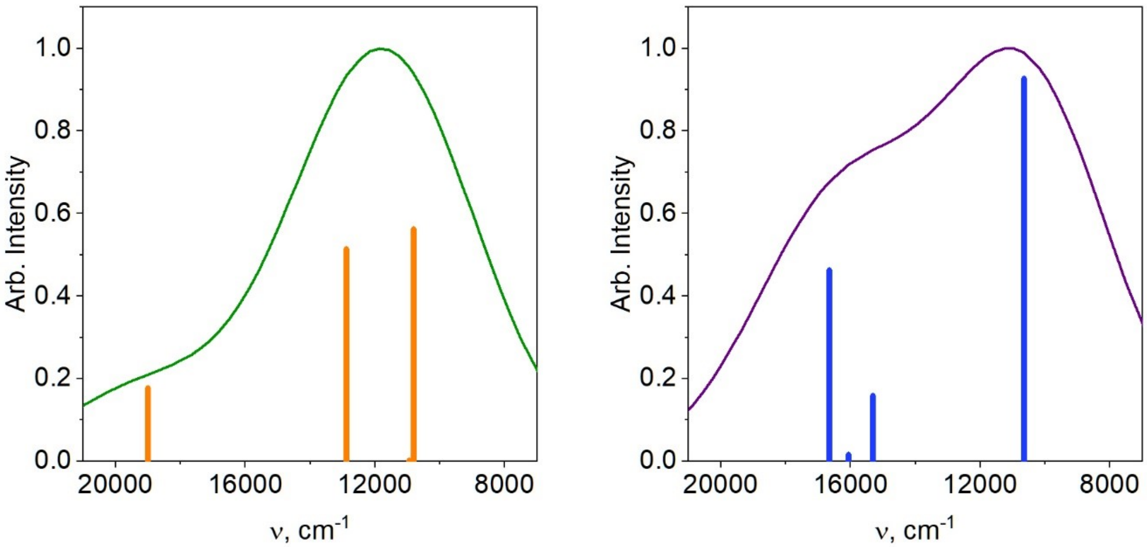

Figure S21 The NEVPT2\CASSCF calculated absorption spectrum of **1-Co** (left) and **1-Cu** (right) arising from electronic transitions $^2A_{1g} \rightarrow {}^2E_g$ (10815 cm^{-1}), $^2A_{1g} \rightarrow {}^2B_{1g}$ (12874 cm^{-1}), and $^2A_{1g} \rightarrow {}^2A_{2g}$ (19017 cm^{-1}) for **1-Co** and from $^2B_{1g} \rightarrow {}^2A_{1g}$ (10656 cm^{-1}), $^2B_{1g} \rightarrow {}^2B_{2g}$ (15315 cm^{-1}), and $^2B_{1g} \rightarrow {}^2E_g$ (16654 cm^{-1}) for **1-Cu**.

2.5 EPR measurements

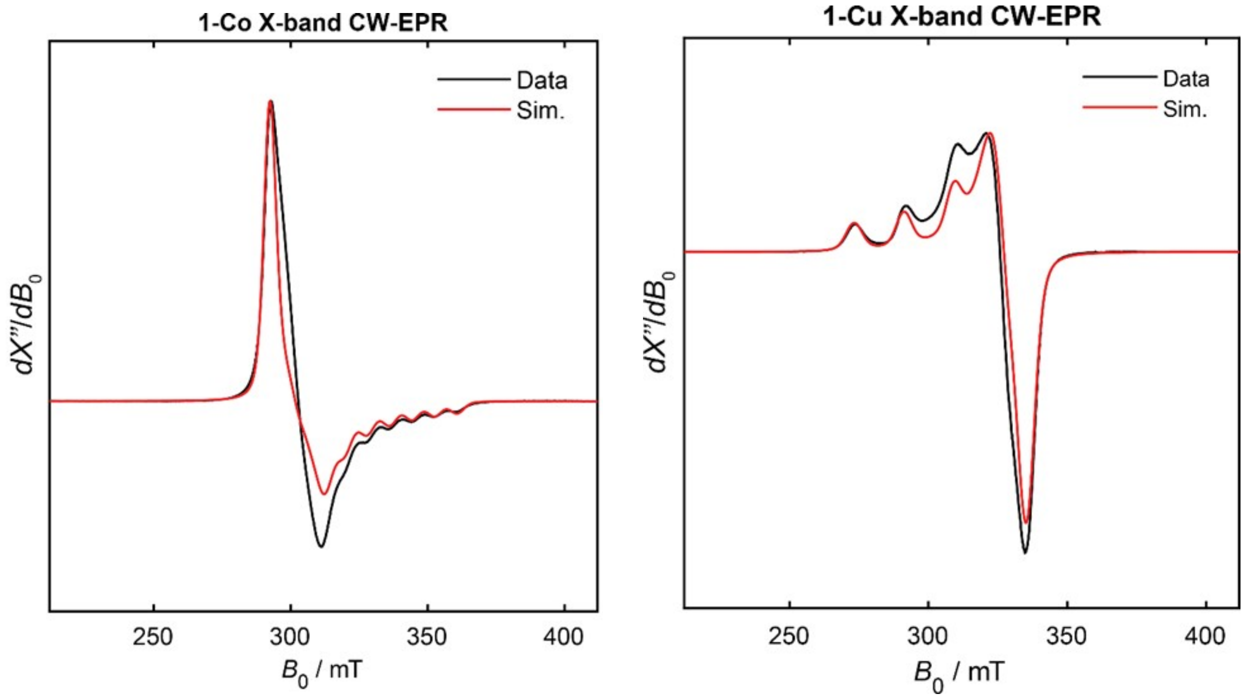

Figure S22 X-band CW-EPR spectra of **1-Co** (left) and **1-Cu** (right), with simulations overlaid in red. Acquisition parameters: X-band CW-EPR: temperature = 77 K (LN_2), MW frequency = 9.390 GHz, MW power = 550 μW, modulation amplitude = 0.8 mT, conversion time = 10 ms.

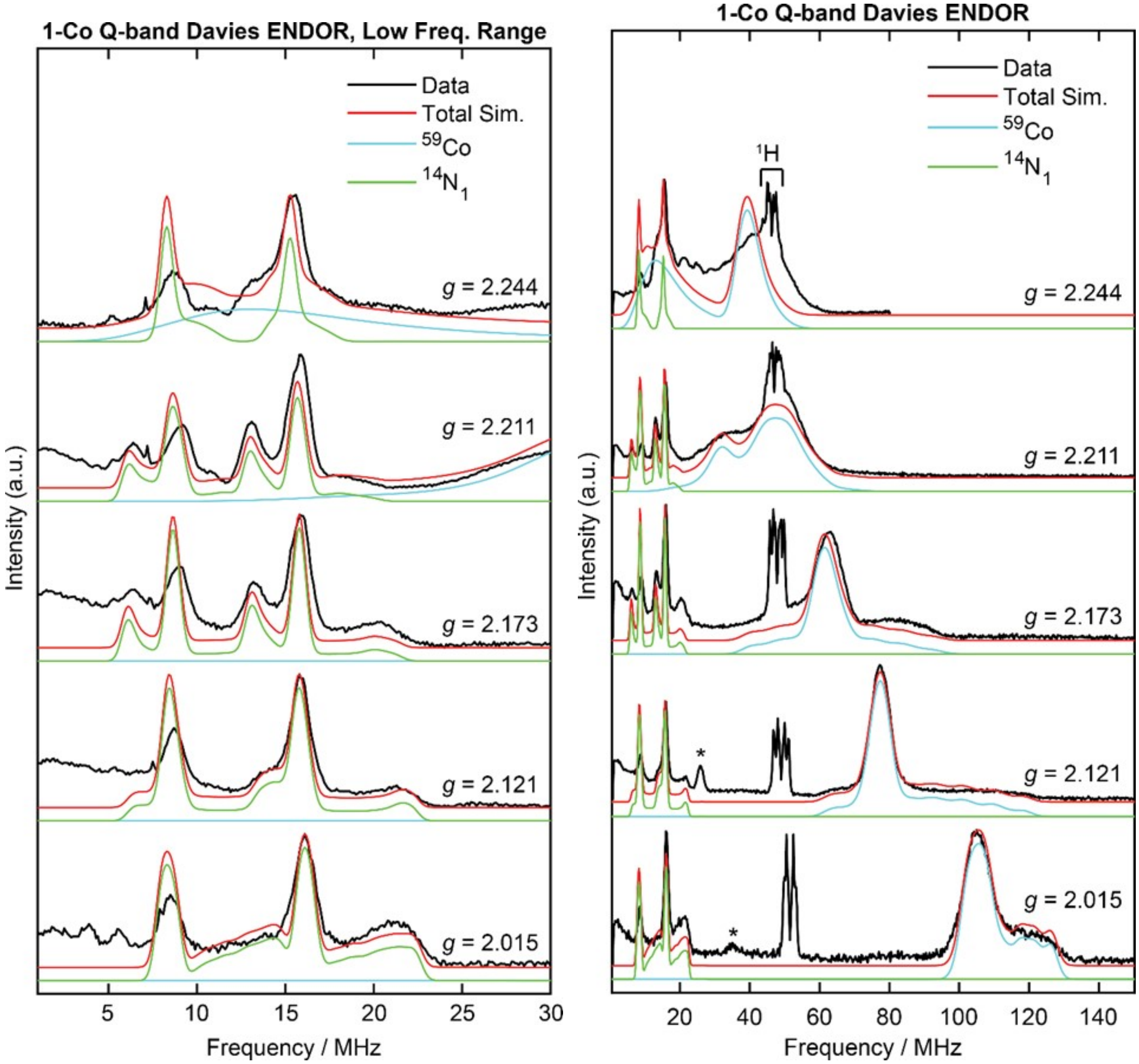

Figure S23 Q-band Davies ENDOR spectra of **1-Co,** with total simulations overlaid in red, and individual nuclear contributions plotted beneath in cyan (^{59}Co) and green ($^{14}N_1$). Asterisks indicate signals arising from 3rd harmonic of intense ^{59}Co signals at higher frequency – these are absent in the low frequency ENDOR because a low-pass RF filter with a cut-off frequency of 35 MHz was used for these spectra. Sharp, non-simulated signals centered around c.a. 50-52 MHz are from weakly coupled ^{1}H nuclei of ligand. Acquisition parameters: Low Freq. ENDOR: τ = 400 ns, MW π pulse length = 160 ns, RF π pulse length = 40 μs. Wide ENDOR spectra: τ = 240 ns, MW π pulse length = 160 ns, RF π pulse length = 40 μs. Both ENDOR data sets: temperature = 7.5 K, MW frequency = 34.133 GHz, T_{RF} delay = 2 μs, shot rep. time = 20 ms.

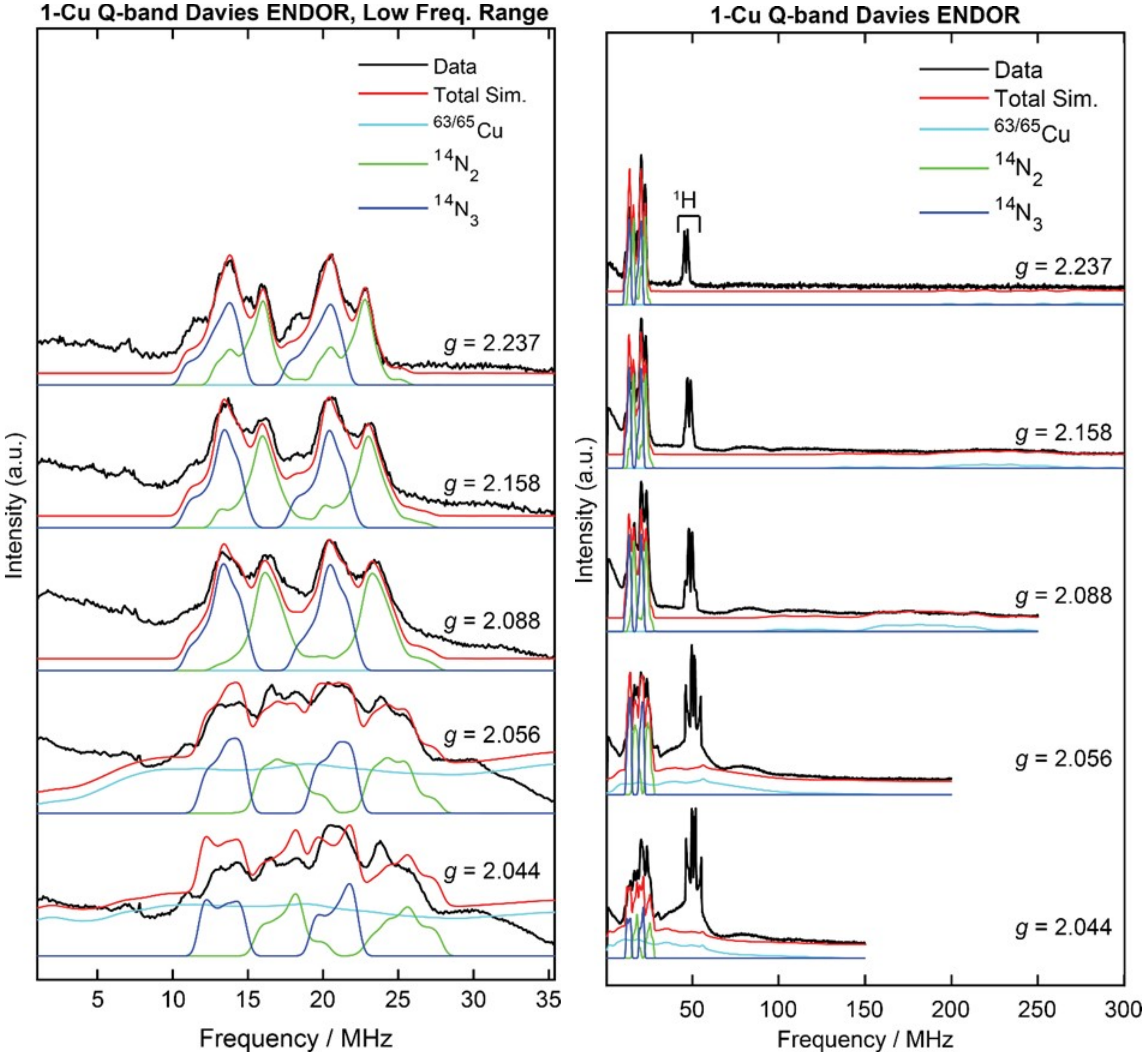

Figure S24 Q-band Davies ENDOR spectra of **1-Cu** with total simulations overlaid in red, and individual nuclear contributions plotted beneath in cyan ($^{63/65}$Cu), green ($^{14}N_2$), and blue ($^{14}N_3$). Unsimulated signals centered around c.a. 50-52 MHz in wider ENDOR spectra are from weakly coupled ^{1}H nuclei of ligand. Acquisition parameters: Low Freq. ENDOR: τ = 400 ns, MW π pulse length = 160 ns, RF π pulse length = 40 µs. Wide ENDOR spectra: τ = 240 ns, MW π pulse length = 160 ns, RF π pulse length = 40 µs. Both ENDOR data sets: temperature = 10 K, MW frequency = 34.125 GHz, T_{RF} delay = 2 µs, shot rep. time = 20 ms.

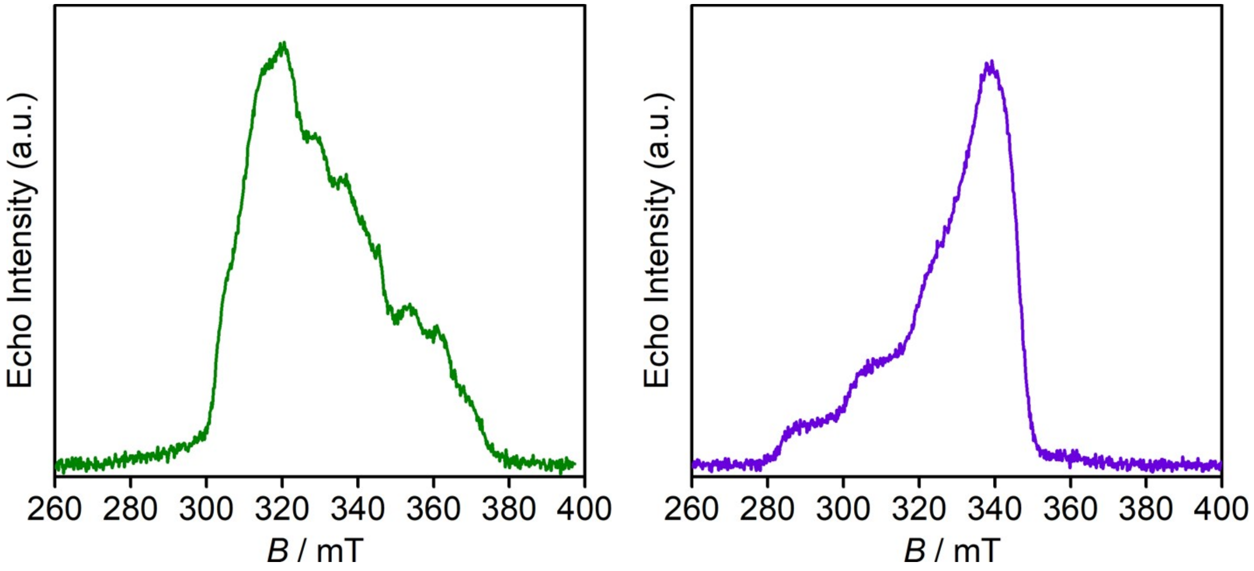

Figure S25 Echo-detected field-swept spectra recorded at the X-band (9.714 GHz) for frozen solutions (ca. 1 mM) of **1-Co** (left) and **1-Cu** (right) in acetonitrile-d_3: toluene-d_8 (1:1) at 10 K and 20 K respectively.

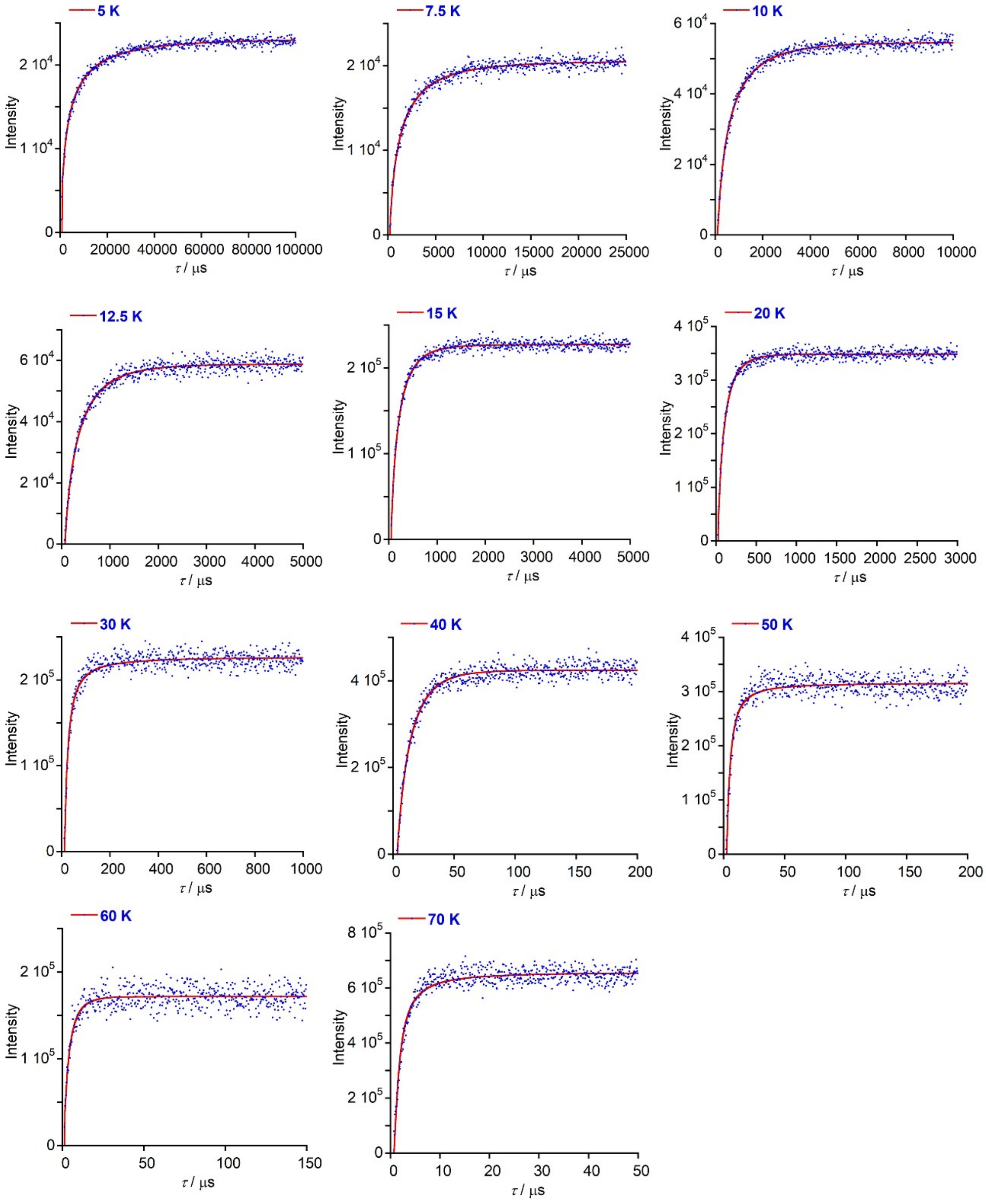

Figure S26. Variable-temperature inversion recovery curves for frozen solution (ca. 1 mM) of **1-Co** in acetonitrile-d_3: toluene-d_8 (1:1) measured at 313.4 mT. Red solid lines represent the best fits to the exponential decays using a stretched monoexponential equation.

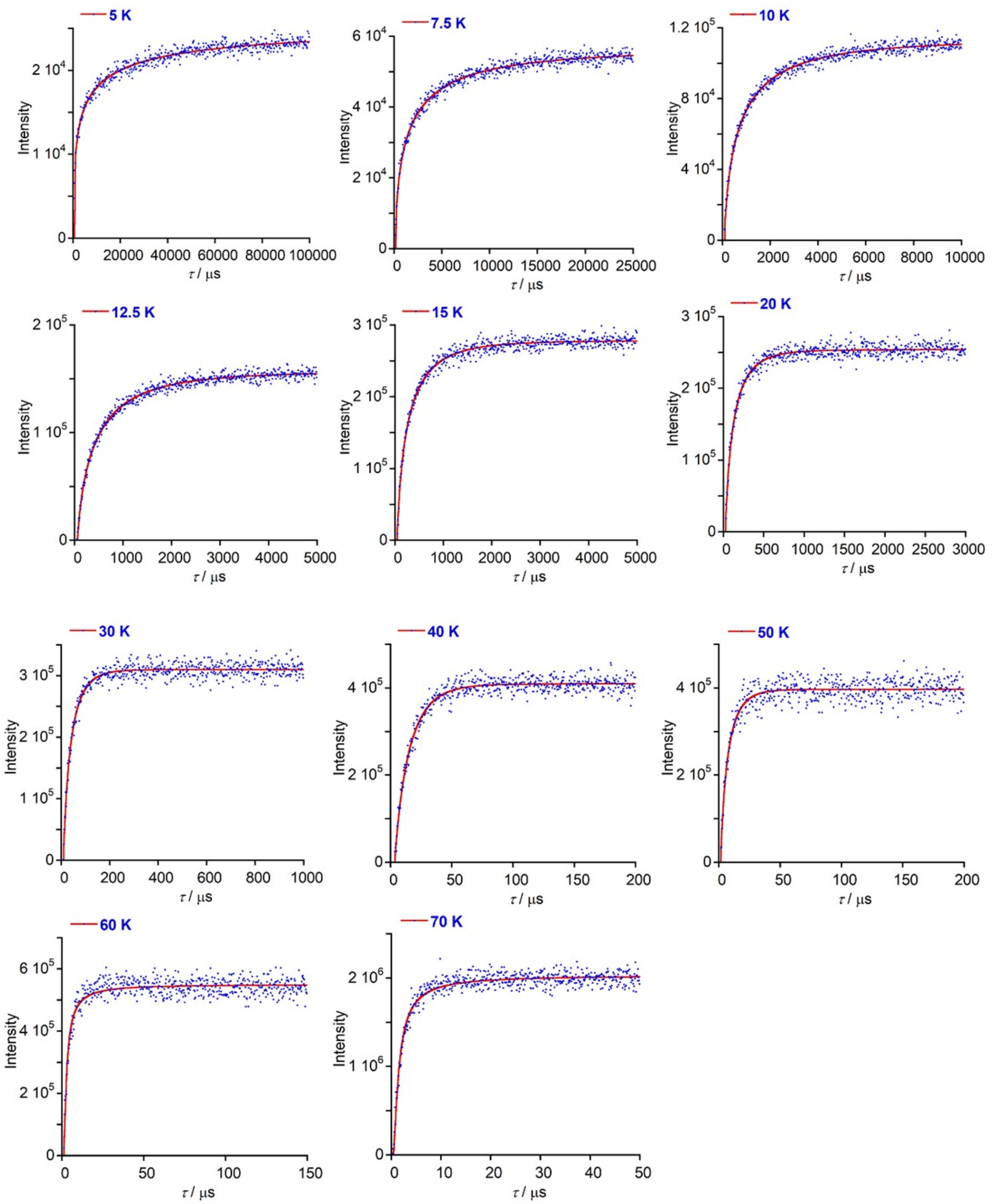

Figure S27 Variable-temperature inversion recovery curves for frozen solution (ca. 1 mM) of **1-Co** in acetonitrile-d_3: toluene-d_8 (1:1) measured at 341.5 mT. Red solid lines represent the best fits to the exponential decays using a stretched monoexponential equation.

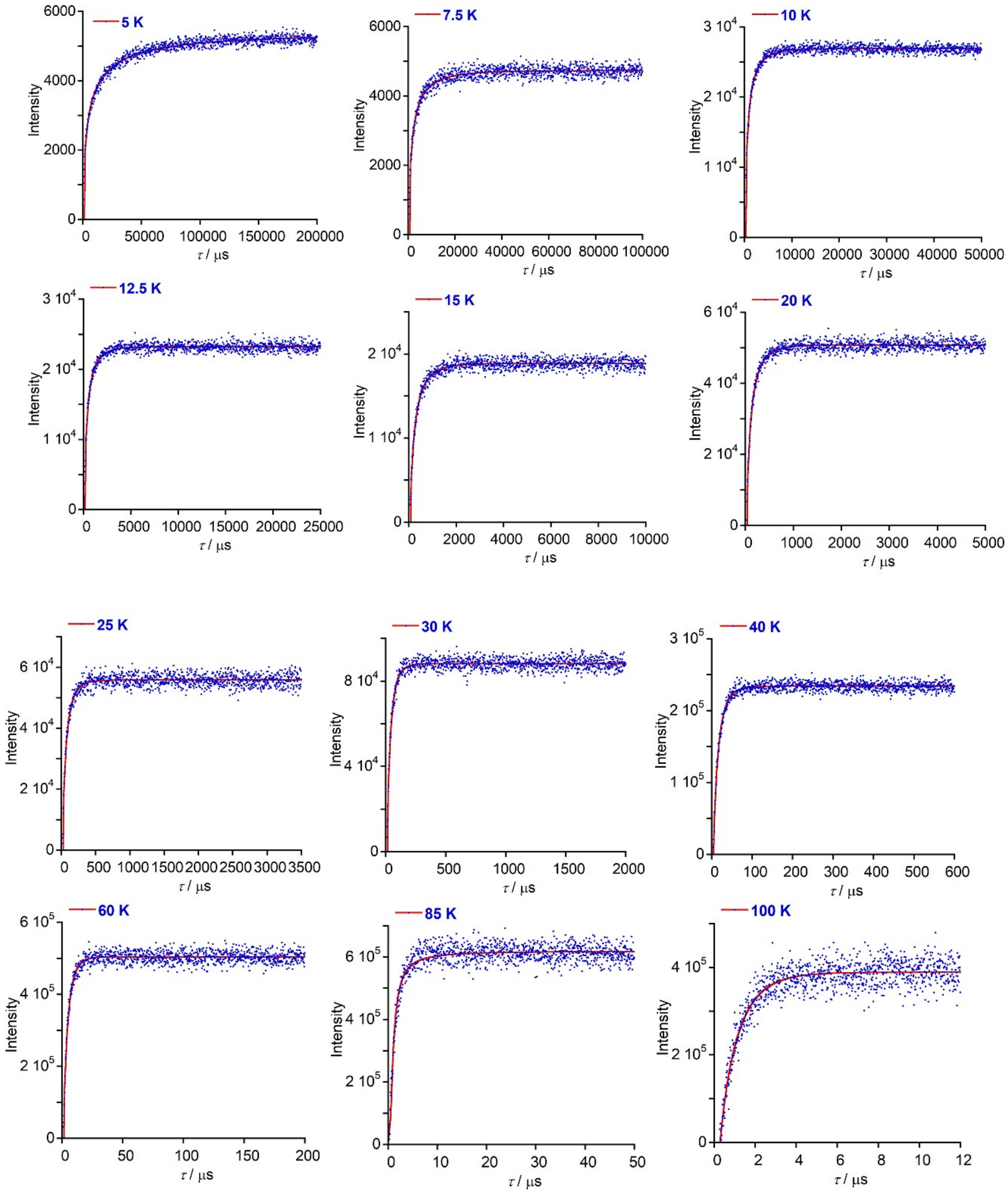

Figure S28 Variable-temperature inversion recovery curves for frozen solution (ca. 1 mM) of **1-Cu** in acetonitrile-d_3: toluene-d_8 (1:1) measured at 312.7 mT. Red solid lines represent the best fits to the exponential decays using a stretched monoexponential equation.

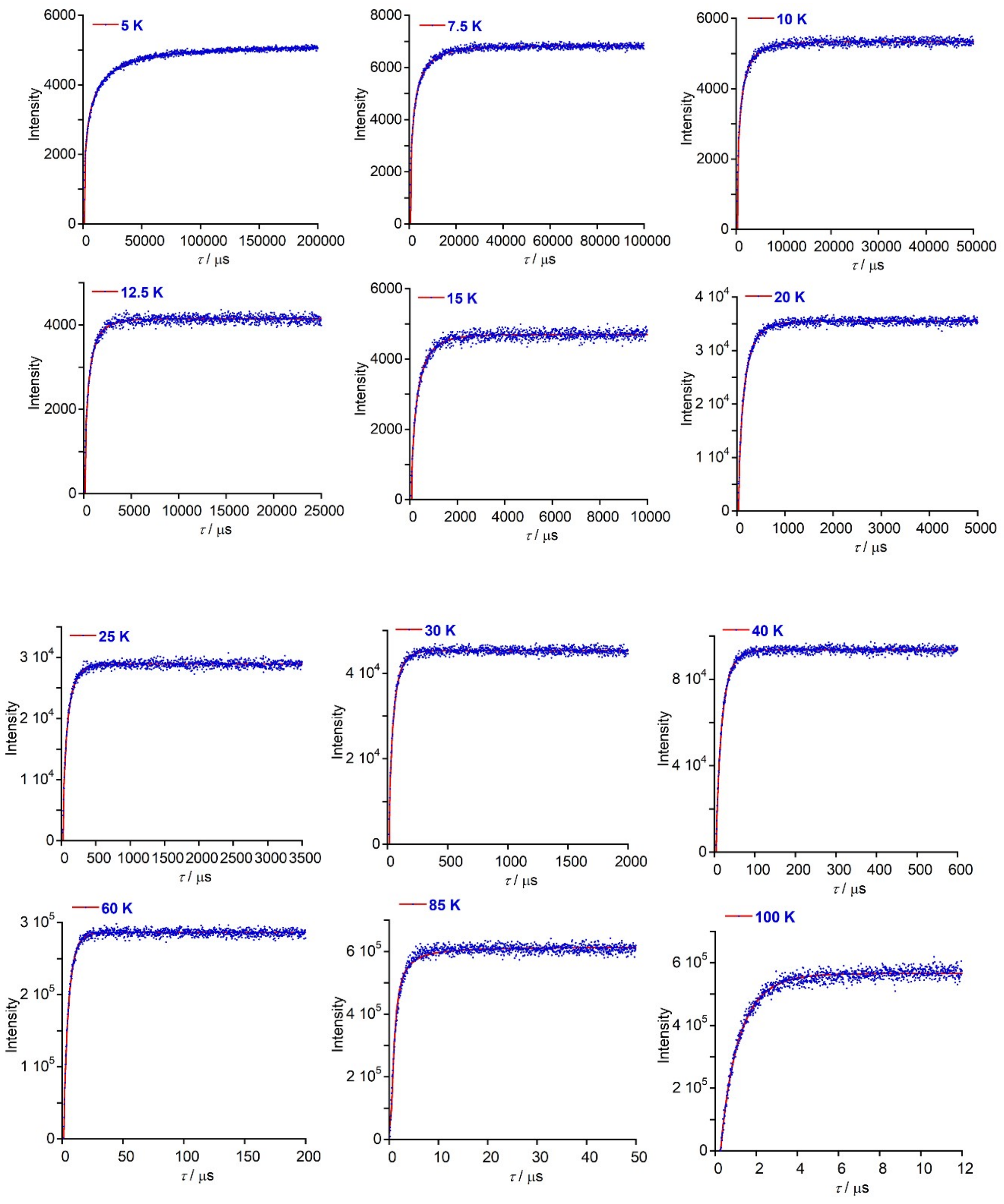

Figure S29 Variable-temperature inversion recovery curves for frozen solution (ca. 1 mM) of **1-Cu** in acetonitrile-d_3: toluene-d_8 (1:1) measured at 340.28 mT. Red solid lines represent the best fits to the exponential decays using a stretched monoexponential equation.

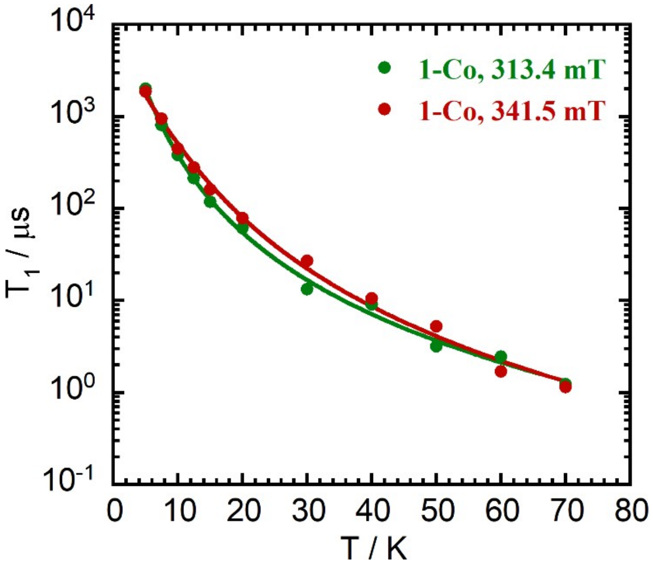

Figure S30 Temperature dependence of electron spin relaxation time: spin–lattice relaxation (T_1) for complex **1-Co** at 313.4 mT and 341.5 mT in acetonitrile-d_3: toluene-d_8 (1:1). Full lines are the best fits of the models with the combination of direct and Raman processes.

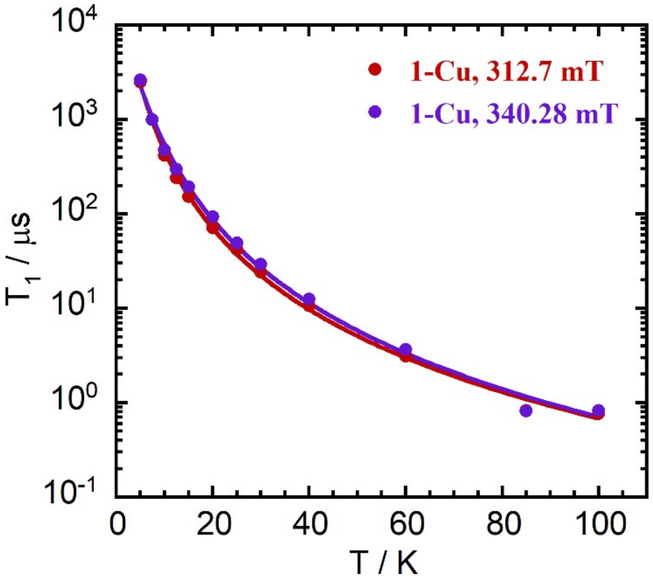

Figure S31 Temperature dependence of electron spin relaxation time: spin–lattice relaxation (T_1) for complex **1-Cu** at 312.7 mT and 340.28 mT in acetonitrile-d_3: toluene-d_8 (1:1). Full lines are the best fits of the models with the combination of direct and Raman processes.

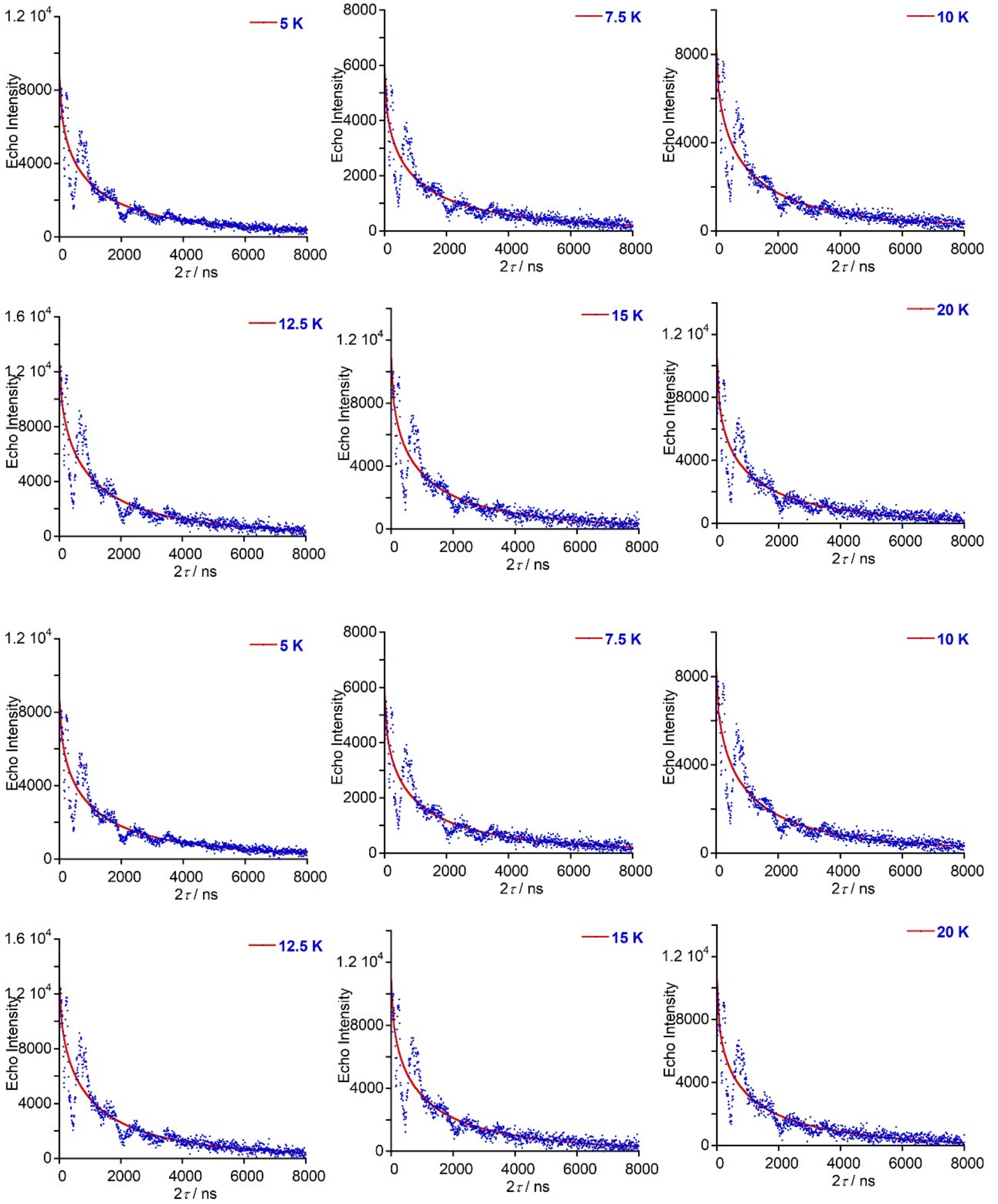

Figure S32 Variable-temperature Hahn echo decay curves for frozen solution (ca. 1 mM) of **1-Co** in acetonitrile-d_3: toluene-d_8 (1:1) measured at 313.4 mT. Red solid lines represent the best fits to the exponential decays using a stretched monoexponential equation.

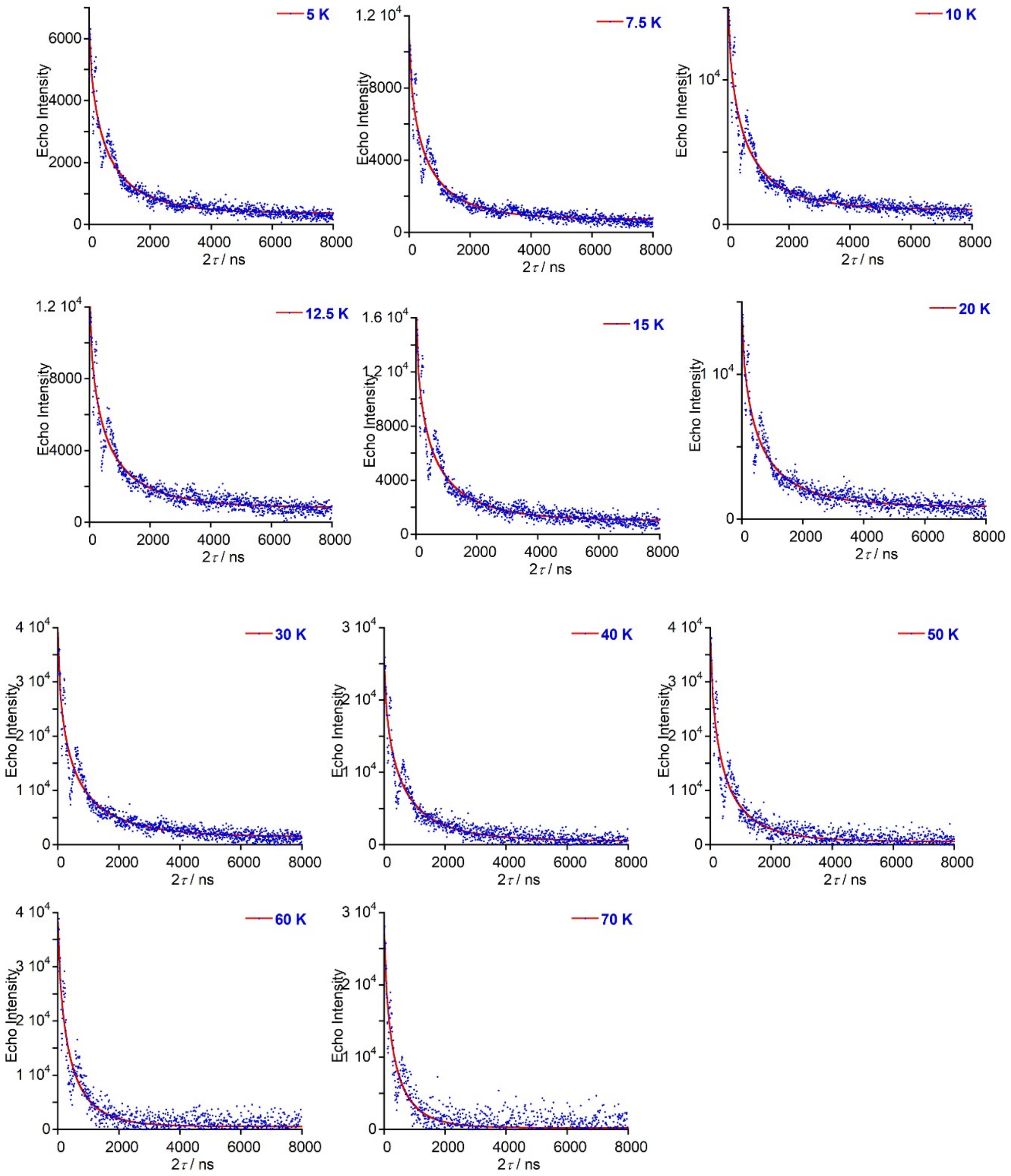

Figure S33 Variable-temperature Hahn echo decay curves for frozen solution (ca. 1 mM) of **1-Co** in acetonitrile-d_3: toluene-d_8 (1:1) measured at 341.5 mT. Red solid lines represent the best fits to the exponential decays using a stretched monoexponential equation.

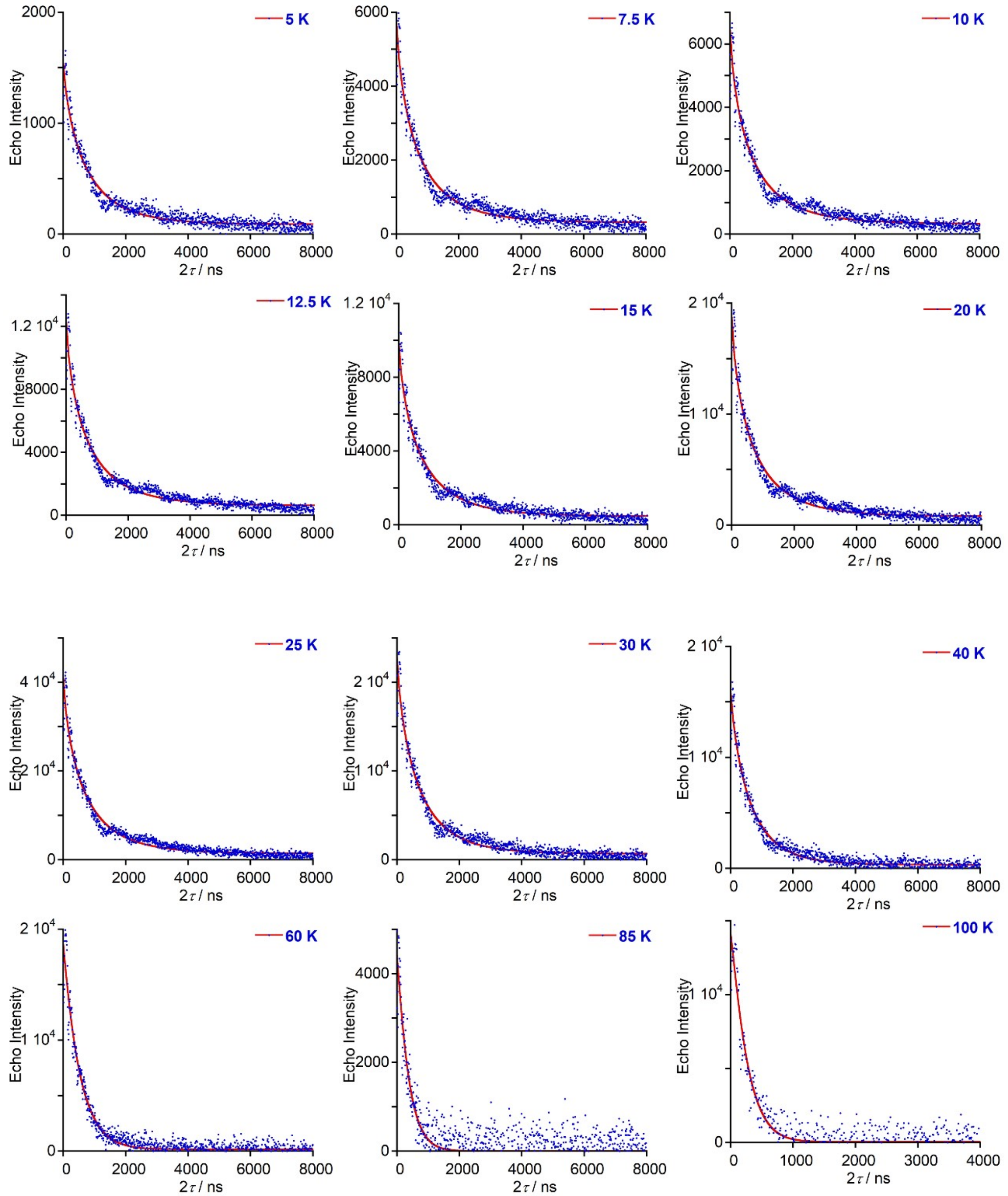

Figure S34 Variable-temperature Hahn echo decay curves for frozen solution (ca. 1 mM) of **1-Cu** in acetonitrile-d_3: toluene-d_8 (1:1) measured at 312.7 mT. Red solid lines represent the best fits to the exponential decays using a stretched monoexponential equation.

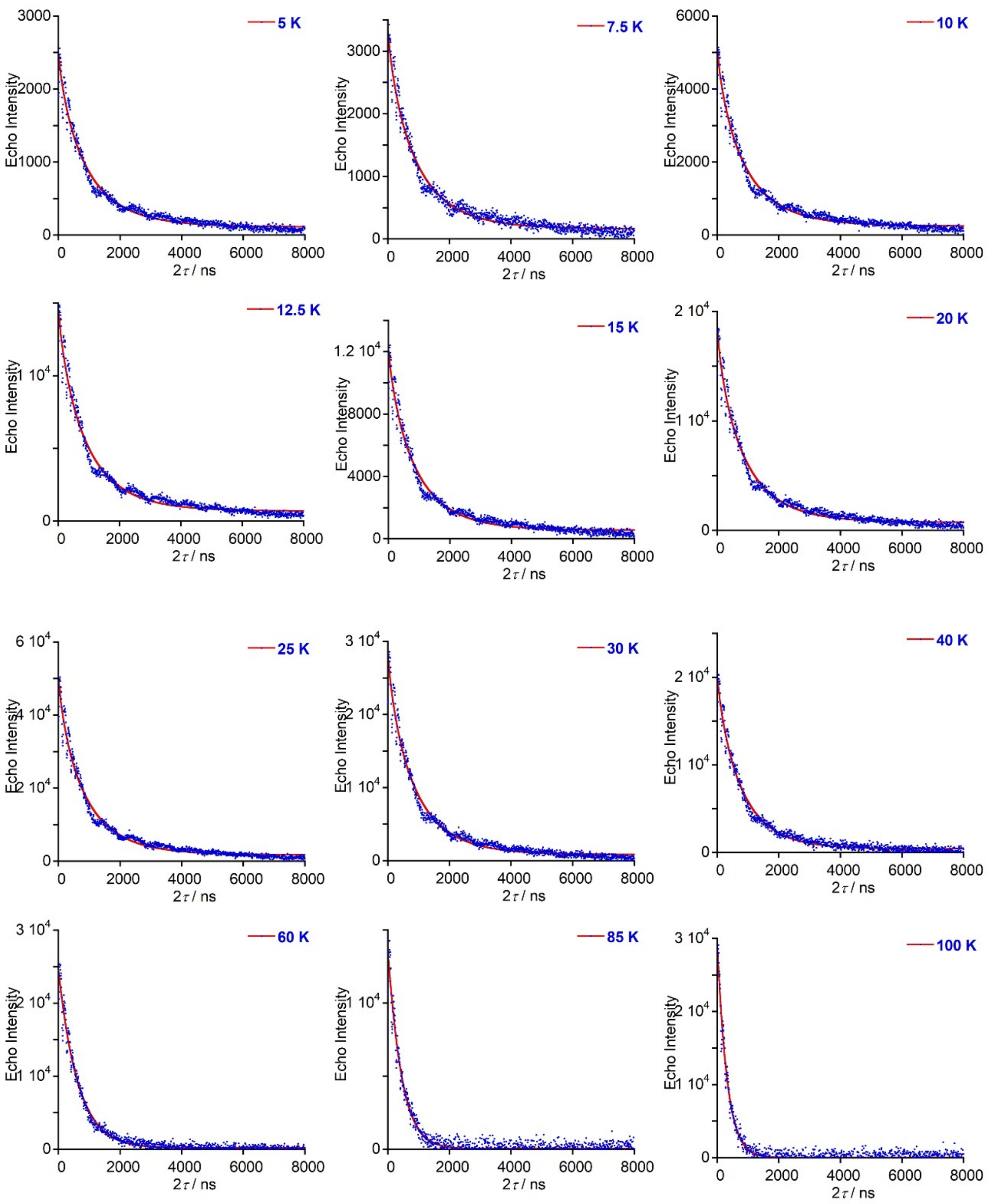

Figure S35 Variable-temperature Hahn echo decay curves for frozen solution (ca. 1 mM) of **1-Cu** in acetonitrile-d_3: toluene-d_8 (1:1) measured at 340.28 mT. Red solid lines represent the best fits to the exponential decays using a stretched monoexponential equation.

Table S10 Temperature-dependent T_1 and T_m data collected at selected field positions at X-band for **1-Co** in acetonitrile-d_3: toluene-d_8 (1:1).

Temperature (K)	Complex **1-Co**			
	313.4 mT ($g_{\parallel}$)		341.5 mT ($g_{\perp}$)	
	T_1 (μs)	T_m (μs)	T_1 (μs)	T_m (μs)
5.0000	2006	0.81851	1870.6	0.52384
7.5000	804.48	0.80448	951.5	0.50741
10.000	383.93	0.84741	448.98	0.51161
12.500	214.58	0.85423	280.2	0.47974
15.000	118.35	0.82440	162.23	0.47386
20.000	61.456	0.75635	78.773	0.52507
30.000	13.344	0.69906	26.88	0.46050
40.000	9.06	0.62218	10.551	0.48135
50.000	3.1827	0.55912	5.2313	0.40262
60.000	2.4499	0.44733	1.7005	0.36154
70.000	1.2283	0.35360	1.1447	0.31565

Table S11 Temperature-dependent T_1 and T_m data collected at selected field positions at X-band for complex **1-Cu** in acetonitrile-d_3: toluene-d_8 (1:1).

Temperature (K)	Complex **1-Cu**			
	312.7 mT ($g_\perp$)		340.28 mT ($g_\parallel$)	
	T_1 (μs)	T_m (μs)	T_1 (μs)	T_m (μs)
5.0000	2510.7	0.69997	2633.4	0.84416
7.5000	1001.2	0.65764	994.07	0.84523
10.000	417.54	0.65173	475.71	0.83124
12.500	241.88	0.65402	298.67	0.83187
15.000	152.95	0.66503	193.57	0.82526
20.000	71.003	0.64757	93.079	0.80247
25.000	41.978	0.64585	49.227	0.79177
30.000	24.302	0.64418	29.397	0.78891
40.000	10.664	0.61692	12.434	0.75179
60.000	3.1064	0.53716	3.657	0.63250
85.000	0.82005	0.38442	0.81492	0.45213
100.00	0.76732	0.27672	0.82778	0.32344

Table S12 Temperature-dependent β_1 and β_m data collected at selected field positions at X-band for complex **1-Co** in acetonitrile-d_3: toluene-d_8 (1:1).

Temperature (K)	Complex **1-Co**			
	313.4 mT ($g_{\|}$)		341.5 mT ($g_{\perp}$)	
	β_1	β_m	β_1	β_m
5.0000	0.42617	0.53615	0.27476	0.65489
7.5000	0.51638	0.51428	0.40789	0.64175
10.000	0.59072	0.55016	0.46964	0.64058
12.500	0.65125	0.54789	0.55249	0.60268
15.000	0.6553	0.52861	0.57105	0.59682
20.000	0.69315	0.48465	0.64837	0.63303
30.000	1.2711	0.54344	0.75748	0.58426
40.000	0.77604	0.58391	0.83697	0.62288
50.000	1.4279	0.63101	0.76126	0.63928
60.000	0.69237	0.62623	1.2962	0.68882
70.000	1.3894	0.61115	1.2644	0.68499

Table S13 Temperature-dependent β_1 and β_m data collected at selected field positions at X-band for complex **1-Cu** in acetonitrile-d$_3$: toluene-d$_8$ (1:1).

Temperature (K)	Complex **1-Cu**			
	312.7 mT ($g_\perp$)		340.28 mT ($g_\parallel$)	
	β_1	β_m	β_1	β_m
5.0000	0.33353	0.79437	0.37053	0.84094
7.5000	0.45682	0.76048	0.46132	0.8445
10.000	0.52128	0.74272	0.50875	0.84396
12.500	0.57961	0.74419	0.58634	0.85018
15.000	0.61056	0.75304	0.6233	0.86119
20.000	0.63589	0.75897	0.68148	0.84133
25.000	0.69776	0.7721	0.70408	0.84511
30.000	0.75774	0.79644	0.73909	0.85035
40.000	0.79333	0.84705	0.81031	0.88388
60.000	0.82118	1.0353	0.87876	1.0119
85.000	1.4666	1.0919	1.3985	1.0593
100.00	0.85812	1.1401	0.88382	1.0447

Table S14 Best-Fit Parameters to Reproduce the Temperature Dependence of the Spin−Lattice Relaxation Rate for **1-Co** and **1-Cu**.

Complex	Field (mT)	a (s^{-1} K^{-1})	b (s^{-1} K^{-n})	n
**1-Co**	314.5	46(21)	2.2(7)	3.0(1)
**1-Co**	341.5	98.0(4)	0.39(1)	3.41(1)
**1-Cu**	312.7	36.2(1)	2.12(1)	2.92(1)
**1-Cu**	340.28	56(15)	1.1(4)	3.05(9)

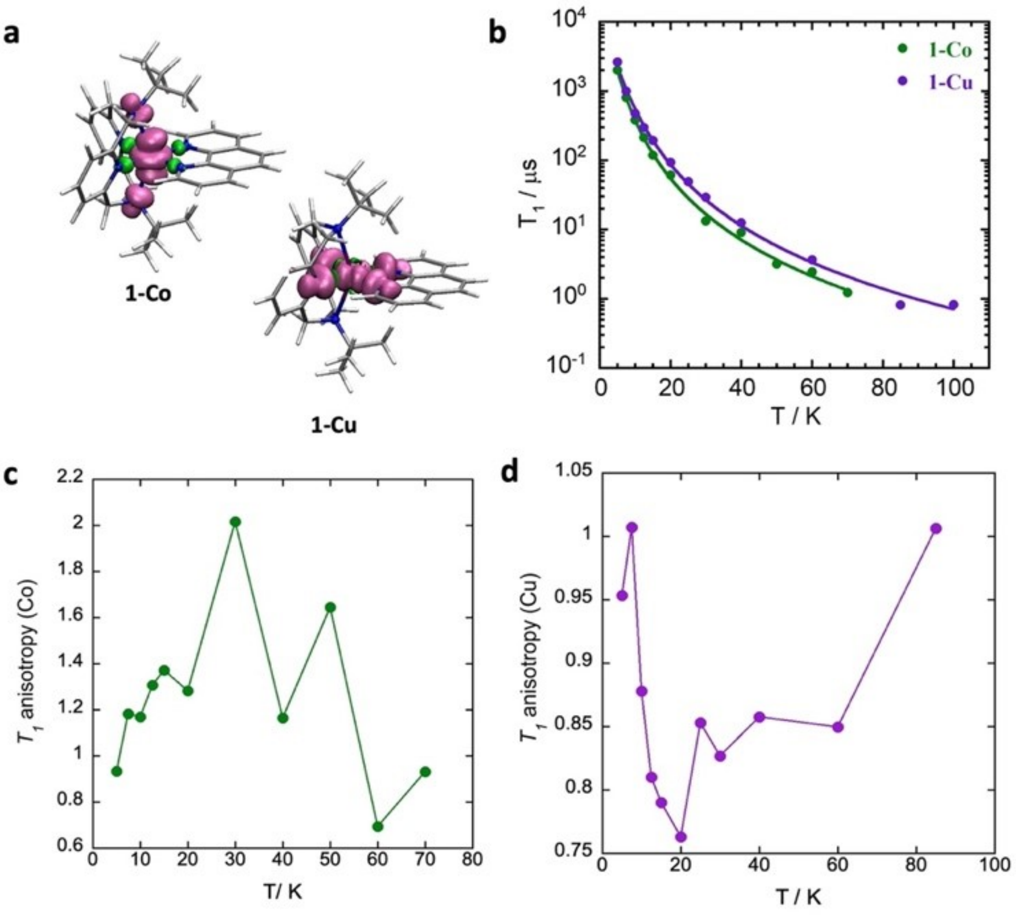

Figure S36 (a) Total spin density (calculated using the B3PW91/EPR-II+CP(PPP) method (*see* ESI for details), (b) Temperature dependence of spin–lattice relaxation (T_1) for complex **1-Co** at 341.5 mT ($g_{||}$) and **1-Cu** at 340.28 mT ($g_{\perp}$) in acetonitrile-d$_3$: toluene-d$_8$ (1:1), full lines are the best fits of the models with the combination of direct and Raman processes, (c) T_1 anisotropy for **1-Co** and (d) T_1 anisotropy for **1-Cu** in a dilute solvent matrix.

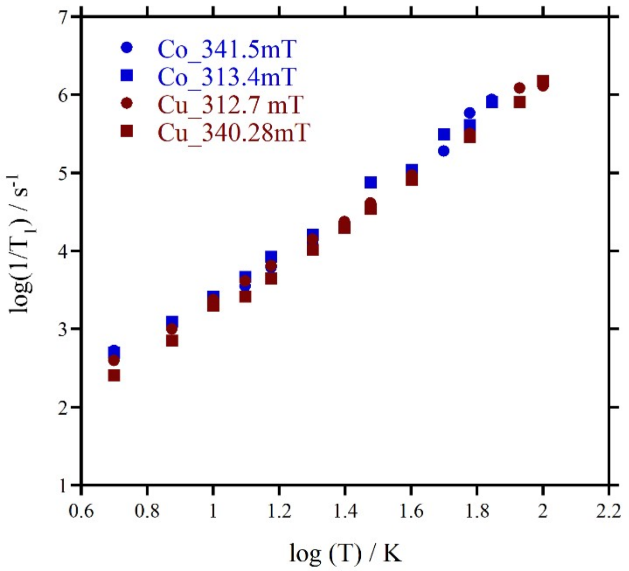

Figure S37 Temperature dependence of spin–lattice relaxation. Log–log plots of the spin–lattice relaxation rate ($1/T_1$) versus temperature for complexes **1-Co** (blue) and **1-Cu** (maroon) measured at the indicated magnetic fields in acetonitrile-d_3:toluene-d_8 (1:1).

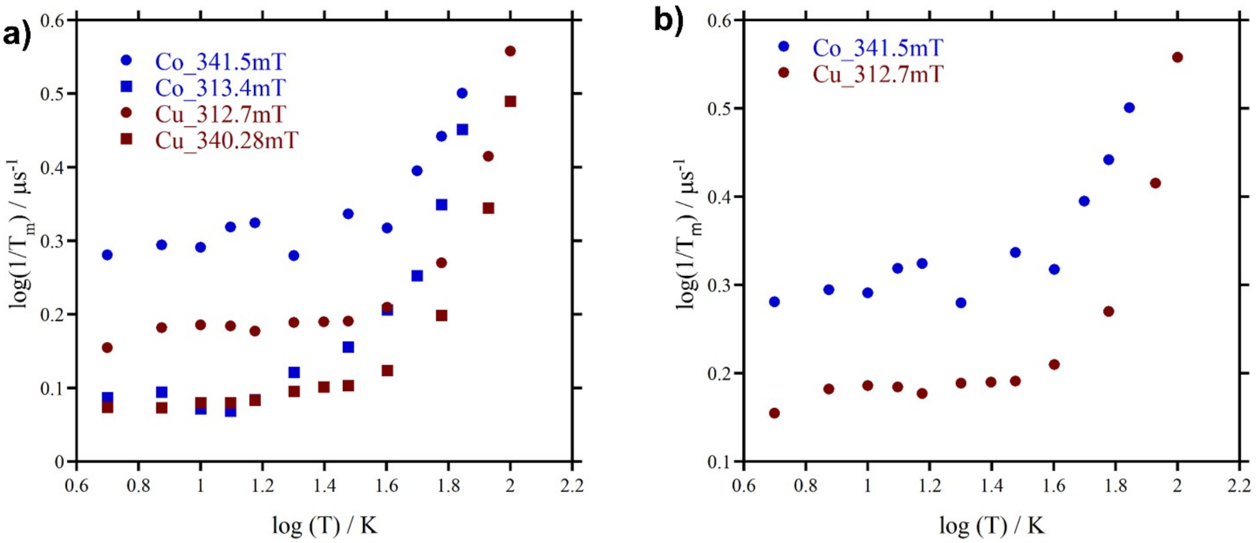

Figure S38 Temperature dependence of phase memory time. Log–log plots of the phase memory relaxation rate ($1/T_m$) as a function of temperature for complexes **1-Co** (blue) and **1-Cu** (maroon) measured at the indicated magnetic fields in acetonitrile-d_3:toluene-d_8 (1:1). a) Field-dependent measurements at multiple magnetic field positions. b) Representative data at selected fields.

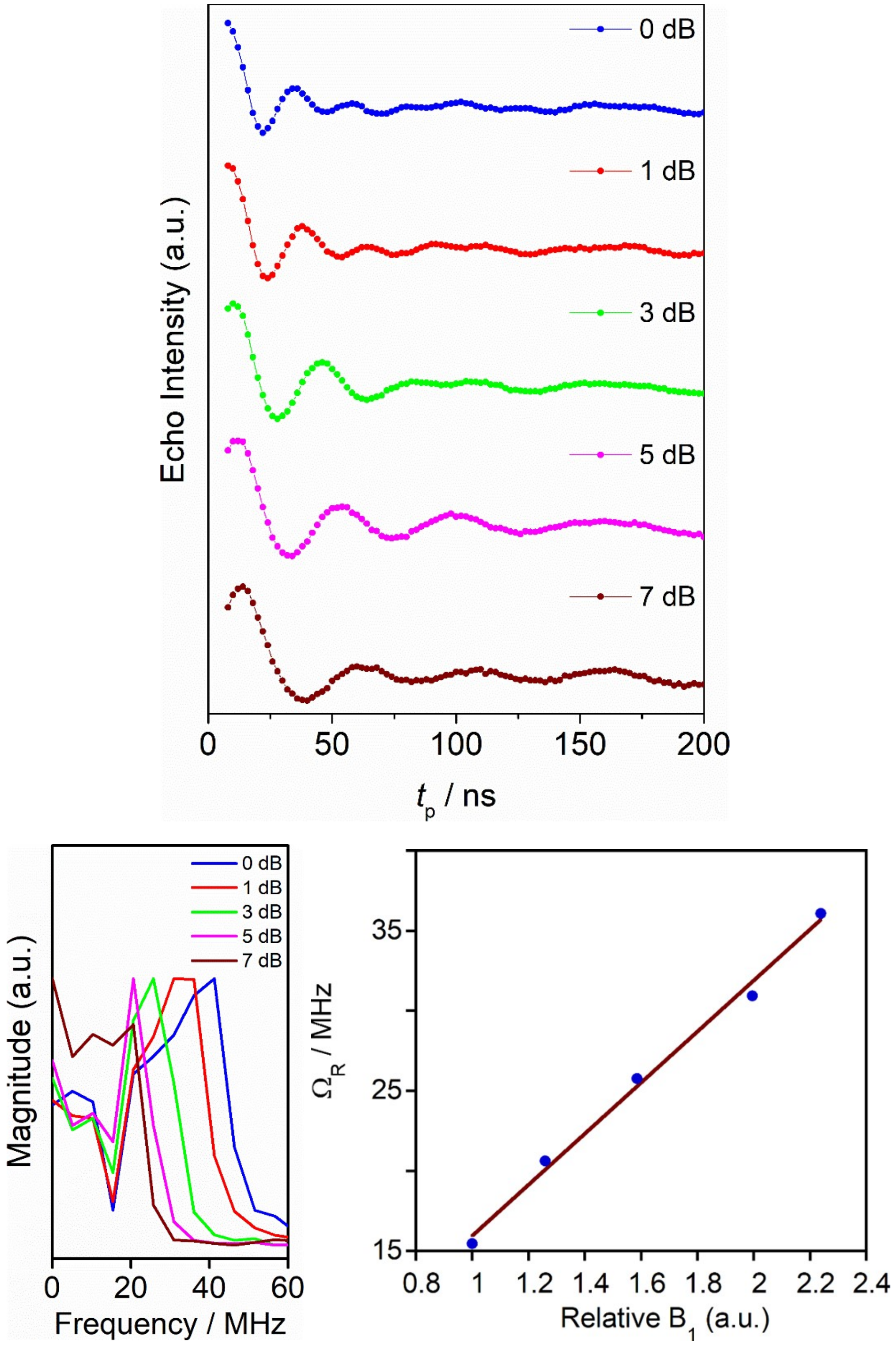

Figure S39 Top: Rabi oscillations for complex **1-Co** in acetonitrile-d_3: toluene-d_8 (1:1) were recorded at 20 K for different microwave attenuations (0 – 7 dB) at 341.5 mT. Bottom: Fourier transform of the Rabi oscillations (left) and linear dependence of the Rabi frequency (Ω_R) as a function of the relative microwave attenuation B_1. The relative microwave attenuation is calculated relative to the weakest microwave power examined (7 dB).

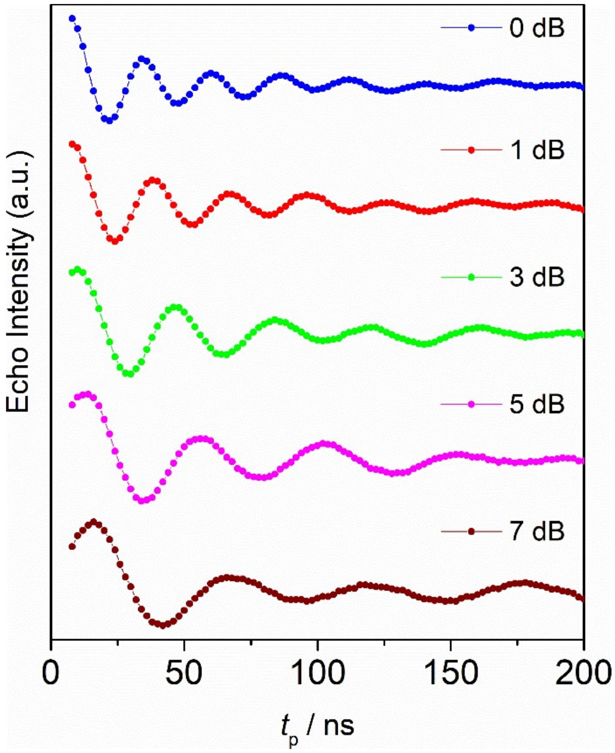

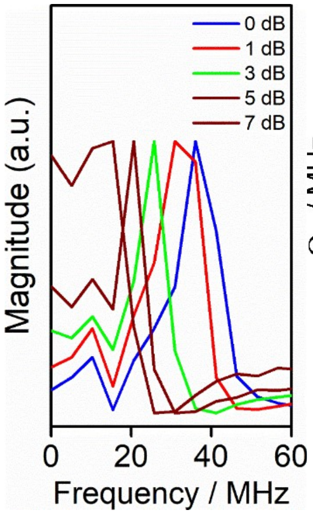

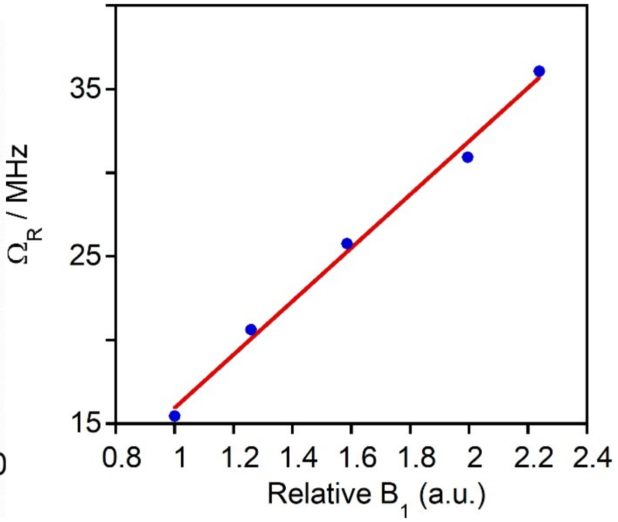

Figure S40 Top: Rabi oscillations for complex **1-Cu** in acetonitrile-d_3: toluene-d_8 (1:1) were recorded at 20 K for different microwave attenuations (0 – 7 dB) at 312.7 mT. Bottom: Fourier transform of the Rabi oscillations (left) and linear dependence of the Rabi frequency (Ω_R) as a function of the relative microwave attenuation B_1. The relative microwave attenuation is calculated relative to the weakest microwave power examined (7 dB).

2.6. Electronic structure calculations

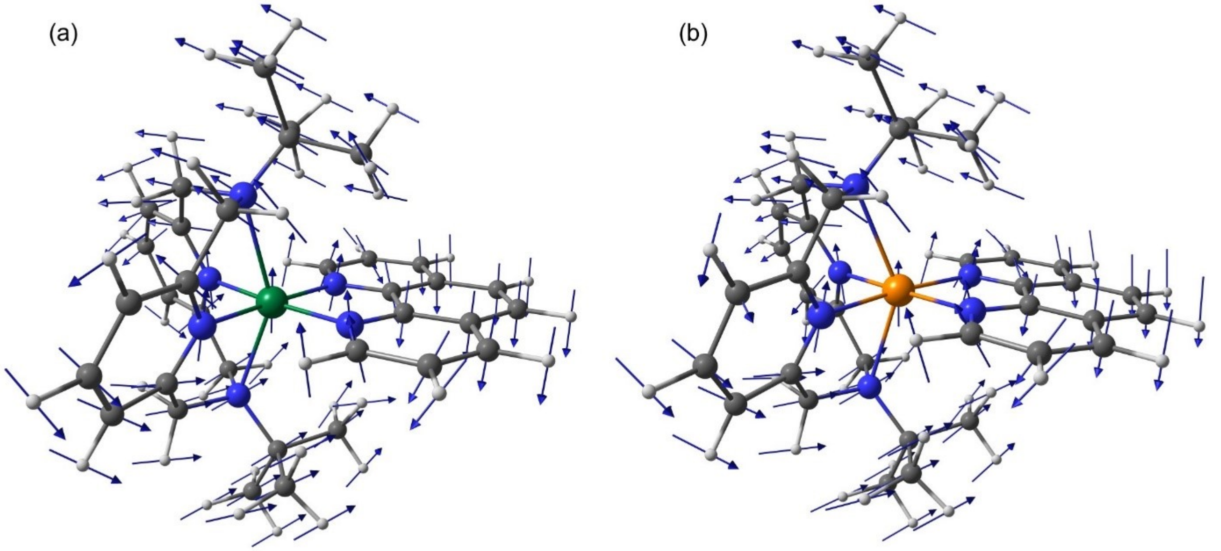

Figure S41 The lowest frequency vibrational modes: (a) **1-Co** (38.49 cm^{-1}), (b) **1-Cu** (34.05 cm^{-1}). The arrows are showing relative displacements of atoms.

Visualization of the selected vibrational modes.

Two trios of quasi-degenerate vibrational modes, ν_{8-10} and ν_{11-13}, were selected as candidates for the modes responsible for the Raman mechanism of magnetic relaxation in **1-Co** and **1-Cu** (see Table S15). Here, in contrast to the main text, the vibrational modes are counted starting from mode 7 (the first 6 modes are translational and rotational). The ν_8 modes, with vibrational frequencies of 47.40 cm^{-1} (**1-Co**) and 40.55 cm^{-1} (**1-Cu**), correspond to the N(Py)–Co/Cu–N(Phen) bending vibrations. The ν_9 modes, with vibrational frequencies of 48.55 cm^{-1} (**1-Co**) and 43.91 cm^{-1} (**1-Cu**), correspond to the N(Py)–Co/Cu–N(Py) bending vibrations. The final modes of the ν_{8-10} trio, the ν_{10} modes, correspond to the N(Phen)–Co/Cu–N(Phen) twisting with frequencies of 51.17 cm^{-1} (**1-Co**) and 47.24 cm^{-1} (**1-Cu**).

The next sets of modes are closer in energy and characterized by vibrational frequencies of approximately 70 cm^{-1}. For **1-Co**, ν_{11} (71.01 cm^{-1}), corresponds to the bending of the N(amino)–Co–N(amino) angle, which causes a significant displacement of tert-butyl groups. For the **1-Cu** complex, ν_{13} (71.24 cm^{-1}) corresponds to the same vibration. Finally, the ν_{12} (72.51 cm^{-1}) and ν_{13} (73.44 cm^{-1}) modes of **1-Co** correspond to symmetrically equivalent displacements caused by rotation of tert-butyl groups around the N–C bonds. The equivalent modes of **1-Cu** (ν_{11} and ν_{12}) have frequencies of 69.37 cm^{-1} and 69.79 cm^{-1}, respectively.

Table S15 Selected vibrational modes.

**1-Co**		
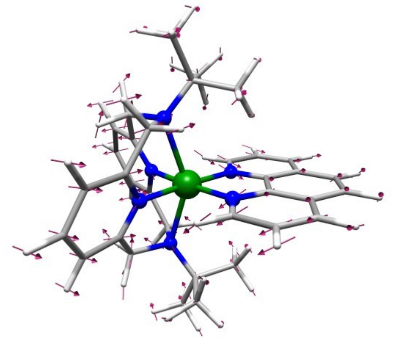	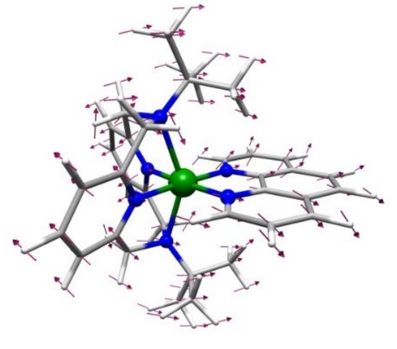	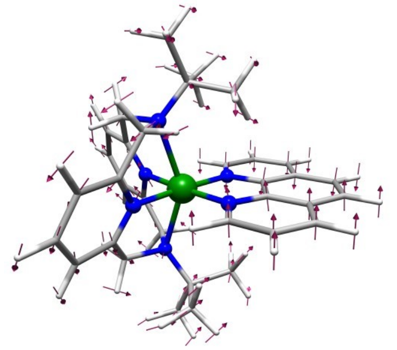
Mode 8, 47.40 cm^{-1}	Mode 9, 48.55 cm^{-1}	Mode 10, 51.17 cm^{-1}

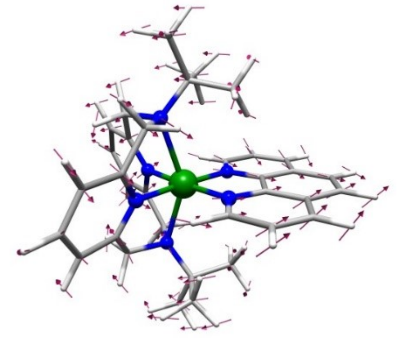	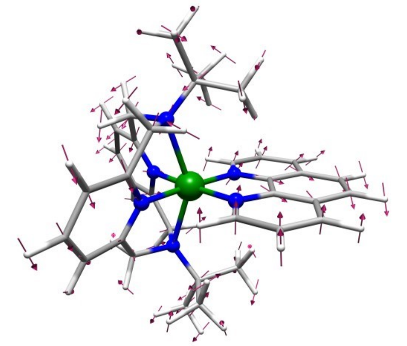	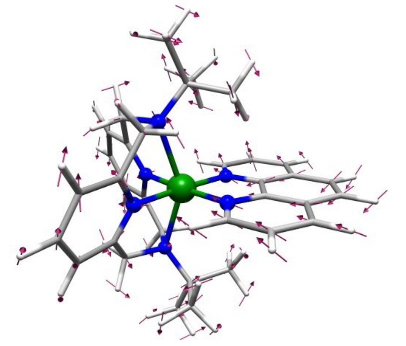
Mode 11, 71.01 cm^{-1}	Mode 12, 72.51 cm^{-1}	Mode 13, 73.44 cm^{-1}
**1-Cu**		
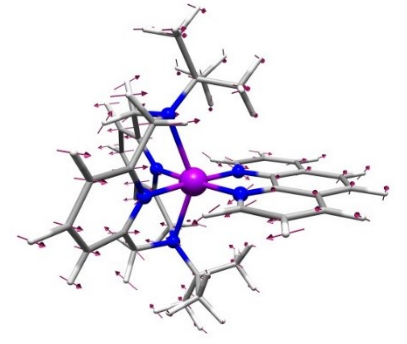	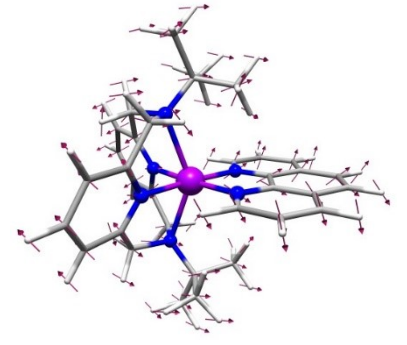	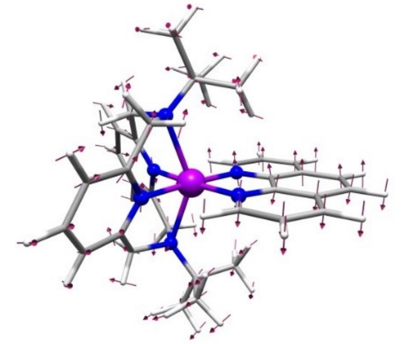
Mode 8, 40.55 cm^{-1}	Mode 9, 43.91 cm^{-1}	Mode 10, 47.24 cm^{-1}
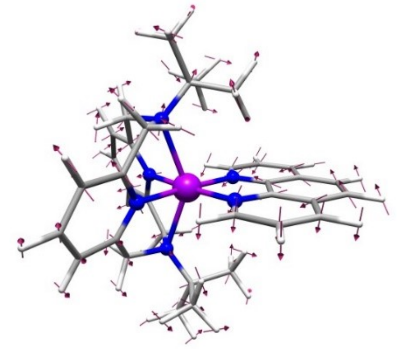	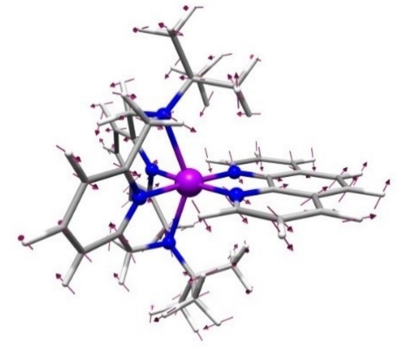	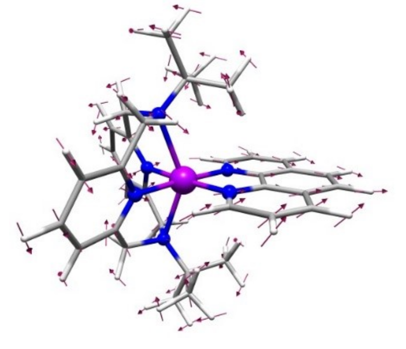
Mode 11, 69.37 cm^{-1}	Mode 12, 69.79 cm^{-1}	Mode 13, 71.24 cm^{-1}

Table S16 Energies (cm^{-1}) and symmetry (Γ) of the ligand field one-electron eigenfunctions (orbitals) calculated by the *ab initio* ligand field theory (AILFT)[28] using the CASSCF (7,5) calculations for **1-Co** and the CASSCF(9,5) calculations for **1-Cu**. The orbital symmetry is given in the approximate D_{4h} symmetry of the complex.

**1-Co**			**1-Cu**		
Orbital	Energy	Γ	Orbital	Energy	Γ
d_{yz}	0	E_g	d_{yz}	0	E_g
d_{xz}	273	E_g	d_{xz}	409	E_g
d_{xy}	1182	B_{2g}	d_{xy}	1144	B_{2g}
d_{z^2}	6679	A_{1g}	d_{z^2}	4004	A_{1g}
$d_{x^2-y^2}$	17688	B_{1g}	$d_{x^2-y^2}$	11858	B_{1g}

Table S17 Dominant configurations, symmetries, and NEVPT2 energies (cm^{-1}) of the thirteen lowest-energy electronic states of **1-Co** which arise from the d^7 electronic configuration of cobalt (II), oscillator strengths for the transitions from the ground doublet state, and reduced SOC matrix elements (cm^{-1}) between the ground state and each of the excited states. Configurations are written as particular occupations of the five active space orbitals. In a short notation, only a singly occupied and unoccupied orbitals are shown. The active space orbitals are composed dominantly of the atomic d orbitals of Co. The symmetries of the states are given in the approximate D_{4h} symmetry of the complex. The e_g and e'_g orbitals correspond to two linear combinations of the d_{xz} and d_{yz} orbitals. Note that the actual C_1 symmetry results in breaking of the degeneracy of the E states.

State	Configuration	Γ	Energy	f_{osc}	SOC
0	$(d_{z^2})^1(d_{x^2-y^2})^0$ (97%)	$^2A_{1g}$	0		
1	$(d_{xy})^1(d_{z^2})^1(d_{x^2-y^2})^1$ (54%) + $(e_g)^1(d_{z^2})^1(d_{x^2-y^2})^1$ (40%)	$^4A_{2g}$+^4E_g	1 961		803
2	$(e_g)^1(d_{z^2})^1(d_{x^2-y^2})^1$ (87%) + $(e'_g)^1(d_{xy})^1(d_{x^2-y^2})^1$ (12%)	^4E_g	2 514		461
3	$(e_g)^1(d_{z^2})^1(d_{x^2-y^2})^1$ (47%) + $(d_{xy})^1(d_{z^2})^1(d_{x^2-y^2})^1$ (41%)	$^4A_{2g}$+^4E_g	2 881		676
4	$(e_g)^1(e'_g)^1(d_{x^2-y^2})^1$ (99%)	$^4B_{1g}$	10 009		20
5	$(e'_g)^1(d_{x^2-y^2})^0$ (81%) + $(e_g)^1(d_{z^2})^1(d_{x^2-y^2})^1$ (15%)	^2E_g	10 815	1.24×10^{-5}	874
6	$(e_g)^1(d_{x^2-y^2})^0$ (80%) + $(e'_g)^1(d_{z^2})^1(d_{x^2-y^2})^1$ (15%)	^2E_g	10 952	6.80×10^{-8}	866
7	$(d_{z^2})^0(d_{x^2-y^2})^1$ (92%)	$^2B_{1g}$	12 874	1.12×10^{-5}	18
8	$(e'_g)^1(d_{xy})^1(d_{x^2-y^2})^1$ (53%) + $(e_g)^1(d_{xy})^1(d_{z^2})^1$ (41%)	^4E_g	16 197		193
9	$(e_g)^1(d_{xy})^1(d_{x^2-y^2})^1$ (51%) + $(e'_g)^1(d_{xy})^1(d_{z^2})^1$ (43%)	^4E_g	16 438		194
10	$(d_{xy})^1(d_{z^2})^1(d_{x^2-y^2})^1$ (91%)	$^2A_{2g}$	19 018	3.88×10^{-6}	56
11	$(e_g)^1(e'_g)^1(d_{x^2-y^2})^1$ (42%) + $(d_{xy})^1(d_{x^2-y^2})^0$ (42%) + $(d_{xy})^1(d_{z^2})^0$ (15%)	$^2B_{1g}$ + $^2B_{2g}$	20 717	3.00×10^{-9}	317
12	$(e_g)^1(d_{z^2})^1(d_{x^2-y^2})^1$ (90%)	^2E_g	20 733	2.00×10^{-9}	20

Table S18 Dominant configurations, symmetries, and NEVPT2 energies (cm^{-1}) of the electronic states of **1-Cu,** which arise from the d^9 electronic configuration of copper (II), oscillator strengths for the transitions from the ground doublet state, and reduced SOC matrix elements (cm^{-1}) between the ground state and each of the excited states. Configurations are written as particular occupations of the five active space orbitals. In a short notation, only a singly occupied orbital is shown. The active space orbitals are composed dominantly of the atomic d orbitals of Cu. The e_g and e'_g orbitals correspond to two linear combinations of the d_{xz} and d_{yz} orbitals.

State	Configuration	Γ	Energy	$f_{osc} \times 10^{-6}$	SOC
0	$(d_{x^2-y^2})^1$	$^2B_{1g}$	0		
1	$(d_{z^2})^1$	$^2A_{1g}$	10 656	6.89	165
2	$(d_{xy})^1$	$^2B_{2g}$	15 315	1.23	1 591
3	$(e'_g)^1$	^2E_g	16 070	0.123	805
4	$(e_g)^1$	^2E_g	16 654	3.52	818

Table S19 Symmetry of the orbital part of the spin-orbit coupling between the ground electronic state of **1-Co** (**1-Cu**) and various excited states in the approximate D_{4h} point group. Symbols Γ, Γ^L, and Γ' denote symmetry of the ground state, the angular momentum operator $L = [(L_x, L_y), L_z]$, and an excited state. The orbital part (matrix element) is zero if the symmetry product does not contain the totally symmetric irreducible representation, a_{1g}. The a_{1g} representation is highlighted by an underscore when present in the symmetry product.

Γ'	$\Gamma \times \Gamma^L \times \Gamma'$
	**1-Co**: $\Gamma = A_{1g}$
A_{2g}	$A_{1g} \times [e_g, a_{2g}] \times A_{2g} = [e_g, \underline{a_{1g}}]$
B_{1g}	$A_{1g} \times [e_g, a_{2g}] \times B_{1g} = [e_g, b_{2g}]$
B_{2g}	$A_{1g} \times [e_g, a_{2g}] \times B_{2g} = [e_g, b_{1g}]$
E_g	$A_{1g} \times [e_g, a_{2g}] \times E_g = [\underline{a_{1g}} + a_{2g} + b_{1g} + b_{2g}, e_g]$
	**1-Cu**: $\Gamma = B_{1g}$
A_{1g}	$B_{1g} \times [e_g, a_{2g}] \times A_{1g} = [e_g, b_{2g}]$
B_{2g}	$B_{1g} \times [e_g, a_{2g}] \times B_{2g} = [e_g, \underline{a_{lg}}]$
E_g	$B_{1g} \times [e_g, a_{2g}] \times B_{2g} = [\underline{a_{1g}} + a_{2g} + b_{1g} + b_{2g}, e_g]$

It can be seen from Table S19 that in the case of the ideal D_{4h} point group, the excited states of the A_{2g} symmetry would contribute to the anisotropy of the g_z component of the g-tensor in the case of **1-Co**, and the excited states of the B_{2g} symmetry, in the case of **1-Cu**. The excited states of the E_g symmetry would contribute to the anisotropy of the g_{xy} component of the g-tensor in the case of both **1-Co** and **1-Cu**.

Table S20 Principal components of the g-tensor of **1-Co** calculated using different number of the excited states included in the state-averaging. The shifts Δg are reported relative to the free-electron g-value. The cases correspond to cumulative inclusion of the excited states. Each case is different from the previous by inclusion of the next nearest excited state or a group of close-lying states (see Table S16). Case 1 corresponds to 3 quartet states, case 2 – to 4 quartets, case 3 – to 3 doublets and 4 quartets, case 4 – to 4 doublets and 4 quartets, case 5 – to 4 doublets and 6 quartets, case 6 – to 5 doublets and 6 quartets, case 7 – to 7 doubles and 6 quartets, and case 8 – to all 40 doublet and 10 quartet states that arise from the d^7 configuration of Co(II).

Case	g_x	g_y	g_z	Δg_x	Δg_y	Δg_z
1	2.0575	2.0539	2.0403	0.0552	0.0515	0.0379
2	2.0841	2.0830	2.0313	0.0818	0.0807	0.0289
3	2.3303	2.3228	2.0399	0.3279	0.3205	0.0376
4	2.3285	2.3213	2.0487	0.3262	0.3189	0.0464
5	2.3484	2.3408	2.0543	0.3461	0.3384	0.0520
6	2.3517	2.3439	2.0597	0.3494	0.3416	0.0574
7	2.3553	2.3292	2.0613	0.3530	0.3269	0.0589
8	2.3173	2.3096	2.0779	0.3149	0.3073	0.0756

Cases 1 and 2 correspond to inclusion of the three (four) lowest quartet states ($^4A_{2g}$ + ^4E_g, ^4E_g, $^4A_{2g}$ + ^4E_g, and $^4B_{1g}$) in the state-averaging. Since quartet states do not contribute to the g-tensor anisotropy of the ground doublet state through the spin-orbit coupling at the second order, the tensor's anisotropy remains negligible. Inclusion of the first two excited doublet states of the ^2E_g symmetry (case 3) induces the tensor's anisotropy in the xy plane as in accordance with symmetry rules (Table S19). Inclusion of neither the $^2B_{1g}$ state (case 4) nor the ^4E_g states (case 5) contributes to the tensor's anisotropy in the ideal D_{4h} point group, and computationally we observe only a minor change in the principal g values. The small spin-orbit coupling (56 cm^{-1}) between the ground $^2A_{1g}$ state and the excited $^2A_{2g}$ state (case 6), though orbitally allowed, does not contribute much to the tensor's anisotropy in the z direction, which could explain the small anisotropy of the g_z component of the tensor observed experimentally. Inclusion of the $^2B_{1g}$ + $^2B_{2g}$ and ^2E_g states (case 7) does not change the tensor's anisotropy. The spin-orbit coupling of the $^2B_{1g}$ + $^2B_{2g}$ state to the ground state is orbitally forbidden, and the coupling of the ^2E_g state, though orbitally allowed, is negligible (20 cm^{-1}).

Table S21 Principal components of the *g*-tensor of **1-Cu** calculated using different number of the excited states included in the state-averaging. The shifts Δ*g* are reported relative to the free-electron *g*-value. The cases correspond to inclusion of the first excited state (A_{1g}), two first excited states (A_{1g} and B_{2g}), and all the excited states (A_{1g}, B_{2g}, and E_g).

Case	g_x	g_y	g_z	Δg_x	Δg_y	Δg_z
1	2.0020	2.0020	2.0107	-0.0003450	-0.0003450	0.008371
2	1.9912	1.9927	2.3822	-0.01112	-0.009625	0.3799
3	2.0809	2.0809	2.3673	0.0786	0.0796	0.3650

Case 1 corresponds to a single excited state of the A_{1g} symmetry, which, according to Table S18, does not contribute to anisotropy of the *g*-tensor. Distortion from the ideal D_{4h} point group can explain minor deviation of the principal components of the tensor from the free-electron *g*-value. Inclusion of the second excited state (B_{2g}) significantly contributes to tensor's anisotropy in the *z* direction through the spin-orbit coupling between the ground state and given excited state (Table S18). Similarly, inclusion of the last two excited states of the E_g symmetry contributes to tensor's anisotropy in the *xy* plane.

Table S22 Hyperfine coupling constants A_{xx}, A_{yy}, and A_{zz} (MHz) of metals in **1-Co** and **1-Cu** cations calculated at various levels of theory. Geometries of **1-Co** and **1-Cu** cations were preoptimized at the *w*B97M-D3BJ/def2-SVP level of theory.

Case	Level of theory	Co in **1-Co**			Cu in **1-Cu**		
		A_x	A_y	A_z	A_x	A_y	A_z
1	B3PW91/CP(PPP)*	-105.8	-121.0	316.2	39.5	41.6	-558.9
2	B3PW91/aug-cc-pVTZ-J	-96.2	-112.2	324.7	45.3	47.2	-567.5
3	B3PW91/DKH-def2-TZVP	-128.1	-143.5	300.0	-200.8	-202.9	-823.4
4	PBE0/ZORA-def2-TZVP	-124.8	-138.1	300.9	-41.7	-44.1	-677.6
5	PBE0/EPR-II**	169.1	182.2	626.1	-224.2	417.8	419.9
6	*w*B97M-D3BJ/CP(PPP)*	220.0	-231.8	-237.1	-40.7	-43.0	-705.4

Table S23 DFT calculated Mulliken spin density of the unpaired electron spin on the metal and the directly bound donor nitrogen atoms in complexes **1-Co** and **1-Cu**.

**1-Co**	Spin density	**1-Cu**	Spin density
Co(1)	0.926	Cu(1)	0.648
N(1)	0.052	N(1)	0.000
N(2)	-0.001	N(2)	0.103
N(3)	-0.001	N(3)	0.074

Table S24 Combined experimental and DFT predicted hyperfine parameters for **1-Co** and **1-Cu.**

Atom		A_x	A_y	A_z	a_{iso}	**Euler Angles** [a] **(α,β,γ) °**	***Atom***	A_x	A_y	A_z	a_{iso}	**Euler Angles** [a] **(α,β,γ) °**
^{59}Co	Exp.	-46.0	-82.7	230.8	34.0	(0, 0, 0)	^{63}Cu	14.7	50.4	-550.0	-161.6	(0, 0, 0)
	DFT	-105.1	-127.9	321.5	29.5	(1, 0, 0)		37.0	38.0	-570.7	-165.2	(0, 0, 0)
$^{14}N_1$, $^{14}N_1$' *(amine)*	Exp.	21.6	22.2	31.9	25.3	(0, 30,0) (0,-30,0)	$^{14}N_1$, $^{14}N_1$' *(amine)*	N.D.	N.D.	N.D.	N.D.	N.D.
	DFT	21.4	21.4	30.5	24.4	(-123, 52, 122) (-55, 48, 75)		-0.3	-1.1	-1.2	-0.9	(178, 13, -170) (2, 13, -10)
$^{14}N_2$, $^{14}N_2$' *(pyridine)*	Exp.	N.D.	N.D.	N.D.	N.D.	N.D.	$^{14}N_2$, $^{14}N_2$' *(pyridine)*	46.3	39.8	35.8	40.6	(166, 21, -153) (12, -4, -11)
	DFT	-1.3	-2.3	-2.5	-2.0	(-125, 44, 142) (55, 44, -38)		39.7	41.4	54.3	45.1	(159, 14, -126) (21, 14, -54)
$^{14}N_3$, $^{14}N_3$' *(phen.)*	Exp.	N.D.	N.D.	N.D.	N.D.	N.D.	$^{14}N_3$, $^{14}N_3$' *(phen.)*	35.9	33.8	32.1	33.9	(-34, 30, 154) (-136, 28, 62)
	DFT	-1.0	-1.7	-2.1	-1.6	(-90, 38, 93) (90, 38, -87)		32.0	33.0	42.8	35.9	(-56, 5, 89) (-123, 5, 92)

Rotations of hyperfine coordinate frame relative to *g*-tensor. Experimental and computational hyperfine values given in MHz. Computations were done using the B3PW91/EPR-II+CP(PPP) level of theory (see the ESI for details).

Table S25 Combined experimental and DFT predicted nuclear quadrupole coupling parameters for **1-Co** and **1-Cu.**

Atom		***e^2Qq/h***	***η***	**Euler Angles (α,β,γ)[a]**
^{59}Co	Exp.	-63.1	0.11	(0, 0, 0)°
	DFT	-96.7	0.11	(-3, 0, 0) °
$^{14}N_1$, $^{14}N_1$’ *(amine)*	Exp.	-4.00	0.15	(0, 30,0) ° (0,-30,0) °
	DFT	-4.70	0.16	(-135, 39, 139) ° (45, 39, -41) °
$^{14}N_2$, $^{14}N_2$’ *(pyridine)*	Exp.	N.D.	N.D.	N.D.
	DFT	-2.71	0.32	(-93, 34, 98) ° (87, 34, -82) °
$^{14}N_3$, $^{14}N_3$’ *(phen.)*	Exp.	N.D.	N.D.	N.D.
	DFT	-2.64	0.20	(85, 36, -95) ° (-95, 36, 85) °
Atom		***e^2Qq/h***	***η***	**Euler Angles[a] (α,β,γ)** °
^{63}Cu	Exp.	63.2	0.1	(0, 0, 0)°
	DFT	102.2	0.08	(0, 0, 0) °
$^{14}N_1$, $^{14}N_1$’ *(amine)*	Exp.	N.D.	N.D.	N.D.
	DFT	-5.14	0.14	(153, 50, -59) ° (2, 13, -10) °
$^{14}N_2$, $^{14}N_2$’ *(pyridine)*	Exp.	-2.61	0.27	(153, 50, -59) ° (11, -16, 90) °
	DFT	-2.95	0.27	(159, 14, -126) ° (21, 14, -54) °
$^{14}N_3$, $^{14}N_3$’ *(phen.)*	Exp.	-2.67	0.32	(-102, 55, 171) ° (-136, 25, 14) °
	DFT	-2.81	0.22	(-103, 5, 89) ° (-123, 5, 92) °

[a] Rotations of nuclear quadrupole interaction coordinate frame relative to *g*-tensor.

Table S26 The first twenty vibrational modes with frequencies (ν, cm^{-1}), intensities (I, km × mol^{-1}), and types of vibrations [bond stretching (BOND), angle bending (ANG), torsion angle vibrations (TOR)] calculated for **1-Co**.

Mode	ν	I	Vibrations
1	38.49	0.82	TOR C53-N4-C45-C48
2	47.40	0.57	ANG N5-Co1-N6 TOR N5-C48-C70-C68 ANG Co1-N7-C79 ANG Co1-N6-C42
3	48.55	0.44	ANG N3-Co1-N5 TOR N5-C12-C29-C68 TOR C33-C31-C66-C49
4	51.17	0.03	TOR C37-C39-C41-C43 TOR C74-C76-C78-C80 TOR N3-Co1-N6-C35 TOR C39-C41-C43-C80
5	71.01	0.01	BOND Co1-N7
6	72.51	0.52	ANG N3-Co1-N7
7	73.44	0.23	ANG N3-Co1-N5
8	81.80	0.27	BOND C12-C13
9	104.88	0.79	TOR C79-N7-C72-C74 TOR C42-N6-C35-C37
10	110.58	0.00	TOR Co1-N2-C16-C17
11	122.63	0.16	ANG Co1-N7-C72
12	137.37	0.02	BOND Co1-N7 ANG N2-Co1-N4 BOND Co1-N6 ANG C72-N7-C79
13	146.31	0.12	BOND Co1-N7 BOND Co1-N6 ANG Co1-N7-C72
14	155.89	0.13	BOND Co1-N7 BOND Co1-N6
15	182.87	0.11	TOR C35-N6-C42-C41
16	187.71	0.00	TOR N2-Co1-N4-C50 TOR C31-C66-C49-C50 TOR N3-C11-C33-C31
17	193.00	0.03	TOR C11-C33-C31-C66 TOR C12-C29-C68-C70 TOR C49-N3-C11-C33
18	204.53	1.01	TOR C50-N4-C45-C48
19	213.26	0.57	ANG N2-Co1-N6
20	222.01	0.12	BOND Co1-N4 TOR C31-C66-C49-C50 BOND Co1-N2

Table S27 The first twenty vibrational modes with frequencies (ν, cm^{-1}), intensities (I, km × mol^{-1}), and types of vibrations [bond stretching (BOND), angle bending (ANG), torsion angle vibrations (TOR)] calculated for **1-Cu**.

Mode	ν	I	Vibrations
1	34.05	0.46	TOR Cu1-N7-C40-C23 TOR C23-C22-C24-C41
2	40.55	0.48	TOR C23-C22-C24-C41 ANG N3-Cu1-N7
3	43.91	0.33	ANG N3-Cu1-N5 TOR N5-C10-C16-C34
4	47.24	0.11	TOR N3-Cu1-N6-C19 TOR N5-Cu1-N7-C36
5	69.37	0.33	ANG N3-Cu1-N7
6	69.79	0.27	ANG N5-Cu1-N6 TOR Cu1-N2-C12-C13
7	71.24	0.17	TOR C24-C41-C39-C40
8	76.44	0.50	BOND Cu1-N6
9	98.56	0.33	TOR C23-N6-C19-C20 TOR Cu1-N7-C36-C37 BOND Cu1-N6 BOND Cu1-N7
10	104.41	0.02	TOR N4-Cu1-N2-C11
11	110.47	1.42	TOR Cu1-N7-C40-C23
12	120.49	0.63	BOND Cu1-N7 BOND Cu1-N6 ANG Cu1-N7-C36
13	126.57	0.00	BOND Cu1-N6 BOND Cu1-N7 TOR N6-Cu1-N4-C25 ANG N2-Cu1-N4
14	139.77	2.21	TOR Cu1-N7-C40-C23 BOND Cu1-N2 BOND Cu1-N4
15	171.12	0.07	TOR C21-C22-C23-C40 TOR C24-C22-C23-C40 TOR C36-C37-C38-C39 TOR N7-C40-C23-C22
16	184.00	0.07	BOND C27-C28
17	186.32	13.11	TOR Cu1-N7-C40-C23
18	189.02	4.07	TOR C16-C34-C35-C26 TOR C18-C17-C33-C27 TOR C9-C18-C17-C33
19	195.39	0.51	BOND Cu1-N6
20	212.43	7.30	TOR Cu1-N3-C27-C28

Table S28 Magnitude of the Zeeman splitting (in cm^{-1}) calculated for **1-Co** and **1-Cu** at different orientations of the magnetic field of 5000 Oe (0.5 T) relative to the principal axes of the molecular g-tensor.

	Sim. 1	Sim. 2	Sim. 3	Sim. 4	Sim. 5	Average
**1-Co**	0.47	0.50	0.52	0.47	0.52	0.50
**1-Cu**	0.52	0.49	0.48	-	-	0.50

3. References

1. Meneghetti, S. P.; Lutz, P. J.; Fischer, J.; Kress, J., Synthesis and X-ray structure of a monoprotonated salt and of three transition-metal complexes of N,N′-ditertiobutyl-2,11-diaza[3.3](2,6)pyridinophane. *Polyhedron* **2001,** *20* (21), 2705-2710.
2. Ghosh, S.; Selvamani, S.; Mehta, S.; Mondal, A., Reversible thermo-induced spin crossover in a mononuclear cis-dicyanamido-cobalt(ii) complex containing a macrocyclic tetradentate ligand. *Dalton Trans.* **2020,** *49* (27), 9208-9212.
3. Sheldrick, G. M., SADABS Version 2.03, Bruker Analytical X-Ray Systems, Madison, WI, USA, 2000.
4. Bain, G. A.; Berry, J. F., Diamagnetic Corrections and Pascal's Constants. *J. Chem. Educ.* **2008,** *85* (4), 532.
5. Topping, C. V.; Blundell, S. J., A.C. susceptibility as a probe of low-frequency magnetic dynamics. *J. Phys.: Condens. Matter* **2019,** *31* (1), 013001.
6. Tesi, L.; Lucaccini, E.; Cimatti, I.; Perfetti, M.; Mannini, M.; Atzori, M.; Morra, E.; Chiesa, M.; Caneschi, A.; Sorace, L.; Sessoli, R., Quantum coherence in a processable vanadyl complex: new tools for the search of molecular spin qubits. *Chem. Sci.* **2016,** *7* (3), 2074-2083.
7. Atzori, M.; Tesi, L.; Morra, E.; Chiesa, M.; Sorace, L.; Sessoli, R., Room-Temperature Quantum Coherence and Rabi Oscillations in Vanadyl Phthalocyanine: Toward Multifunctional Molecular Spin Qubits. *J. Am. Chem. Soc.* **2016,** *138* (7), 2154-2157.
8. Atzori, M.; Morra, E.; Tesi, L.; Albino, A.; Chiesa, M.; Sorace, L.; Sessoli, R., Quantum Coherence Times Enhancement in Vanadium(IV)-based Potential Molecular Qubits: the Key Role of the Vanadyl Moiety. *J. Am. Chem. Soc.* **2016,** *138* (35), 11234-11244.
9. Evans, D. F., 400. The determination of the paramagnetic susceptibility of substances in solution by nuclear magnetic resonance. *J. Chem. Soc.* **1959,** (0), 2003-2005.
10. Stoll, S.; Schweiger, A., EasySpin, a comprehensive software package for spectral simulation and analysis in EPR. *J. Magn. Reson* **2006,** *178* (1), 42-55.
11. Hyde, J. S.; Pasenkiewicz-Gierula, M.; Jesmanowicz, A.; Antholine, W. E., Pseudo field modulation in EPR spectroscopy. *Appl. Magn. Reson.* **1990,** *1* (3), 483-496.
12. Lada, Z. G.; Sanakis, Y.; Raptopoulou, C. P.; Psycharis, V.; Perlepes, S. P.; Mitrikas, G., Probing the electronic structure of a copper(ii) complex by CW- and pulse-EPR spectroscopy. *Dalton Trans.* **2017,** *46* (26), 8458-8475.
13. Neese, F., Software update: The ORCA program system—Version 5.0. *WIREs Computational Molecular Science* **2022,** *12* (5), e1606.
14. Weigend, F.; Ahlrichs, R., Balanced basis sets of split valence, triple zeta valence and quadruple zeta valence quality for H to Rn: Design and assessment of accuracy. *Phys. Chem. Chem. Phys.* **2005,** *7* (18), 3297-3305.
15. Perdew, J. P.; Burke, K.; Ernzerhof, M., Generalized Gradient Approximation Made Simple. *Phys. Rev. Lett.* **1996,** *77* (18), 3865-3868.
16. Grimme, S.; Ehrlich, S.; Goerigk, L., Effect of the damping function in dispersion corrected density functional theory. *J. Comput. Chem.* **2011,** *32* (7), 1456-1465.
17. Neese, F., Efficient and accurate approximations to the molecular spin-orbit coupling operator and their use in molecular g-tensor calculations. *J. Chem. Phys.* **2005,** *122* (3), 034107.
18. Weigend, F., Accurate Coulomb-fitting basis sets for H to Rn. *Phys. Chem. Chem. Phys.* **2006,** *8* (9), 1057-1065.
19. Becke, A. D., Density-functional thermochemistry. III. The role of exact exchange. *J. Chem. Phys.* **1993,** *98* (7), 5648-5652.

20. Perdew, J. P.; Wang, Y., Accurate and simple analytic representation of the electron-gas correlation energy. *Physical Review B* **1992,** *45* (23), 13244-13249.
21. Neese, F., Prediction and interpretation of the 57Fe isomer shift in Mössbauer spectra by density functional theory. *Inorg. Chim. Acta* **2002,** *337*, 181-192.
22. Mardirossian, N.; Head-Gordon, M., ωB97M-V: A combinatorially optimized, range-separated hybrid, meta-GGA density functional with VV10 nonlocal correlation. *J. Chem. Phys.* **2016,** *144* (21), 214110.
23. Teixeira, F.; Cordeiro, M. N. D. S., Improving Vibrational Mode Interpretation Using Bayesian Regression. *J. Chem. Theory Comput.* **2019,** *15* (1), 456-470.
24. Neese, F.; Wennmohs, F.; Hansen, A.; Becker, U., Efficient, approximate and parallel Hartree–Fock and hybrid DFT calculations. A 'chain-of-spheres' algorithm for the Hartree–Fock exchange. *Chem. Phys.* **2009,** *356* (1), 98-109.
25. Stoychev, G. L.; Auer, A. A.; Neese, F., Automatic Generation of Auxiliary Basis Sets. *J. Chem. Theory Comput.* **2017,** *13* (2), 554-562.
26. Gómez-Piñeiro, R. J.; Pantazis, D. A.; Orio, M., Comparison of Density Functional and Correlated Wave Function Methods for the Prediction of Cu(II) Hyperfine Coupling Constants. *ChemPhysChem* **2020,** *21* (24), 2667-2679.
27. Casanova, D.; Cirera, J.; Llunell, M.; Alemany, P.; Avnir, D.; Alvarez, S., Minimal Distortion Pathways in Polyhedral Rearrangements. *J. Am. Chem. Soc.* **2004,** *126* (6), 1755-1763.
28. Pinsky, M.; Avnir, D., Continuous Symmetry Measures. 5. The Classical Polyhedra. *Inorg. Chem.* **1998,** *37* (21), 5575-5582.
29. Ketkaew, R.; Tantirungrotechai, Y.; Harding, P.; Chastanet, G.; Guionneau, P.; Marchivie, M.; Harding, D. J., OctaDist: a tool for calculating distortion parameters in spin crossover and coordination complexes. *Dalton Trans.* **2021,** *50* (3), 1086-1096.
